# Quasi zenith satellite system-reflectometry for sea-level measurement and implication of machine learning methodology

**DOI:** 10.1038/s41598-022-25994-6

**Published:** 2022-12-12

**Authors:** Kutubuddin Ansari, Hong-Woo Seok, Punyawi Jamjareegulgarn

**Affiliations:** 1 Integrated Geoinformation (IntGeo) Solution Private Limited, New Delhi, India; 2 Department of Geoinformatic Engineering, Inha University, Incheon, South Korea; 3 Space Technology Development Center, KMITL, Prince of Chumphon Campus, Chumphon, Thailand

**Keywords:** Engineering, Mathematics and computing

## Abstract

The tide gauge measurements from global navigation satellite system reflectometry (GNSS-R) observables are considered to be a promising alternative to the traditional tide gauges in the present days. In the present paper, we deliver a comparative analysis of tide-gauge (TG) measurements retrieved by quasi-zenith satellite system-reflectometry (QZSS-R) and the legacy TG recordings with additional observables from other constellations viz. GPS-R and GLONASS-R. The signal-to-noise ratio data of QZSS (L1, L2, and L5 signals) retrieved at the P109 site of GNSS Earth Observation Network in Japan (37.815° N; 138.281° E; 44.70 m elevation in ellipsoidal height) during 01 October 2019 to 31 December 2019. The results from QZSS observations at L1, L2, and L5 signals show respective correlation coefficients of 0.8712, 0.6998, and 0.8763 with observed TG measurements whereas the corresponding root means square errors were 4.84 cm, 4.26 cm, and 4.24 cm. The QZSS-R signals revealed almost equivalent precise results to that of GPS-R (L1, L2, and L5 signals) and GLONASS-R (L1 and L2 signals). To reconstruct the tidal variability for QZSS-R measurements, a machine learning technique, i.e., kernel extreme learning machine (KELM) is implemented that is based on variational mode decomposition of the parameters. These KELM reconstructed outcomes from QZSS-R L1, L2, and L5 observables provide the respective correlation coefficients of 0.9252, 0.7895, and 0.9146 with TG measurements. The mean errors between the KELM reconstructed outcomes and observed TG measurements for QZSS-R, GPS-R, and GLONASS-R very often lies close to the zero line, confirming that the KELM-based estimates from GNSS-R observations can provide alternative unbiased estimations to the traditional TG measurement. The proposed method seems to be effective, foreseeing a dense tide gauge estimations with the available QZSS-R along with other GNSS-R observables.

## Introduction

The global navigation satellite system-reflectometry (GNSS-R) technique has been developed to monitor the tide-gauge (TG) measurements which it relies on the satellite signal strength reflected from the nearby surface of the water^1–4^. The application of GNSS for high-precision positioning directly employs the satellite signal data received by GNSS stations^5^. The reflected signal oscillates after interfering with the multipath surface and can be noted in terms of signal-to-noise ratio (SNR) measurements. Later, the vertical height from the surface of the water is evaluated by examining the SNR amplitude oscillations. It has been recognized that GNSS-R can sense larger areas (hundreds of meters) and capture extreme sea-level changes along the coast^6^.

Several researchers have used the GNSS-R algorithm based on multi-constellation GNSS systems and presented many comparative analyses. Hobiger et al.^7^ investigated the GNSS-R technique by using GPS as well as GLONASS constellations and noticed that both the accuracy and the precision of the GLONASS-based GNSS-R solution are comparable to, or even better than those of the conventional GPS-based GNSS-R solution. Zhang et al.^8^ concluded that the monitoring accuracy of geostationary satellites is better compared to that of geosynchronous orbit satellites. Jin et al.^9^ used the BeiDou constellation system with three SNR frequencies (L2, L6, and L7) to estimate the sea-level changes. They first time used BDS-R to estimate the sea level changes based on SNR measurements and triple-frequency combinations of phase and code, which are compared to TG observations. An interpolation method is used to interpolate the co-located TG observations to get the observations at one time corresponding to the given antenna vertical height. The outcomes of the study point out that BDS SNR and phase combination sea level changes have a perfect agreement with the correlation coefficient of 0.83–0.91and RMSE of less than 0.6 m. The BDS code combination TG does not show a good comparison like other measurements. Recently, Wang et al.^10^ evaluated the sea-level changes based on the SNR measurements of four constellation systems such as GPS, GLONASS, Galileo, and BeiDou. They concluded that the combined result of GNSS-R provided improved accuracy of 40–75% compared to individual performance. These kinds of combine results are beneficial in terms of precision and sampling rate.

The QZSS establish a GPS complementary system to improve several aspects of positioning, for example, integrity, accessibility, accuracy, and reliability, over the Asia–Pacific region in conjunction with the particular orbits to increase the high-elevation signals in Japan^11,12^. The complete QZSS constellation includes three satellites in inclined elliptical geosynchronous orbits which are named quasi-zenith orbits (QZO) satellites. Each QZO satellite has a figure of an eight-shaped ground track which is designed particularly to ensure that at least one of them with a separation of 8 h is almost directly overhead over Japan with an elevation of greater or equal to 60°^13^. The QZSS uses a geostationary orbit for one satellite which is known as the QZS-3 satellite. The QZO satellite-broadcast signals are compatible with the GPS L1C/A signals, along with modernized GPS L1C, L2C signals, and L5 signals. The combination of GPS and QZSS systems delivers an enhanced performance of positioning via the data ranging correction method. This positioning performance is provided through the transmissions of submeter-class performance improved signals, submeter-class augmentation with the integrity Function (L1-SAIF), and L-band Experimental (LEX) from the QZSS constellation.

**Figure 1:**
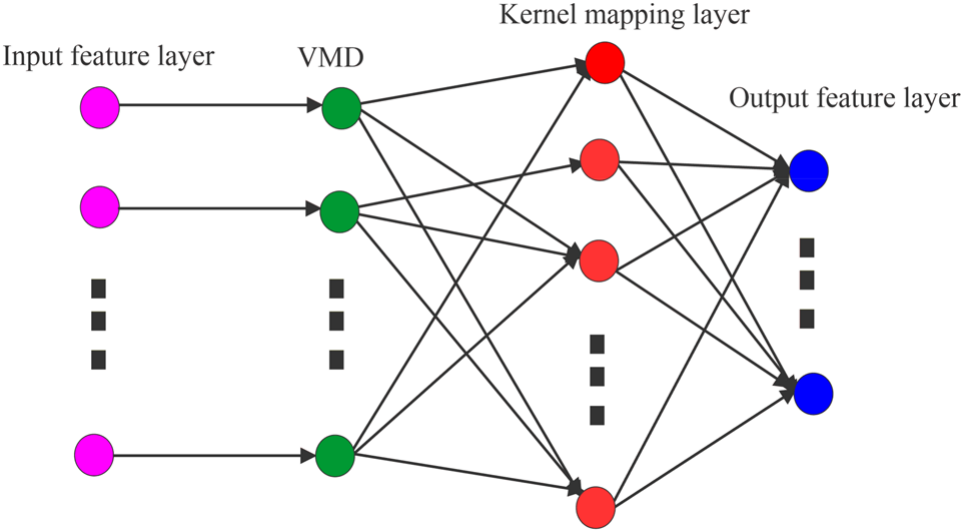
Implementation of VMD-KELM forecasting algorithm with input and output layer.

There are several kinds of methods based on time-series modeling that regular prediction by using statistical methods such as Singular spectrum analysis, Auto–regressive moving average, and machine learning methods^14–17^. These models expect that the rate change in a particular time series is a linear function and may not consider an ability to represent the nonlinear properties. The height of sea level is a kind of dynamic motion in the vertical direction, every time keeps on varying in multiple directions. This means, there is no fixed trend and pattern of water surface. The Artificial Neural Network (ANN) methods can approximate any multivariate function to a desired degree of accuracy by adjusting weightings during online updates and can capture the complex, dynamic and non-linear features of water surface. This kind of computational intelligence method has higher prediction accuracy than statistical approaches^17^. The ANN provides an excellent characteristics approximation that contains a simple structural network by considering the nonlinear nature of the given time series. These methods can achieve impressive results, but usually at the expense of very massive and complicated network architectures. However, it suffers the problem of accurate predictions such as overfitting, local minima, and local maxima. To overcome such kind of modeling and forecasting problem as mentioned above, in the current study, a new approach based on kernel extreme learning machine (KELM) has been utilized. The KELM is an acceptable and meaningful approach to study the prediction of time series with its excellent generalization, universal categorization potential, and rapid training. The hidden neuron of extreme learning machine (ELM) algorithm is very significant among the influencing factors of the ELM learning performance, uses for improving the overview stability and performance of single-hidden layer feedforward neural network. However, choosing the hidden neurons suitable for active combination function is still unresolved^18^. Huang et al.^19^ suggested that when the mapping feature functions of a hidden neuron are unknown, a kernel function can be utilized for the algorithm stability improvement, known as KELM. Li et al.^20^ advised that to improve the performance of the KELM generalization learning algorithm, the kernel parameter should be selected in a proper way. The KELM approach uses kernel functions to deal with situations in which the function of the mapping features of hidden nodes is unknown^19^. Several kinds of kernels such as Exponential kernel, Linear kernel, Polynomial kernel, and Gaussian kernel have been commonly used in combination with extreme machine learning. The KELM algorithm generates random connection weight between input, hidden layers, and threshold of neurons in the hidden layer. In this algorithm, adjustment of the parameters is not needed during the training process. The KELM algorithm provides better results compared to the neural network algorithms because the output matrix, which is obtained by the least square solution process without iteration and reduces the timing of network parameter settling to a great extent. Hence this approach can be considered more suitable for other methods such as the Long short-term memory (LSTM) model and the Holt-Winter method. The KELM approach has been successfully applied in several prediction applications such as selling of property, power wind, prices and etc.^21–24^. In the current study, the approach of variational mode decomposition (VMD) of the KELM technique is used firstly as pre-processing data and original time-series decomposed into a different number of distinct intrinsic mode functions. The method aims to reduce the nonstationary nature of the time-series data (Fig. 1). Initial twelve VMD modes of the time-series are utilized along with KELM for training and validation purposes. The mathematical summary of the KELM approach has been given in “Results and discussions” section.

Numerous kinds of literature have focused on investigating the TG measurements using multi-constellation signals, however, the SNR measurement based on Quasi-Zenith Satellite System (QZSS) constellation signal has never been investigated. The QZSS provides the best service over Asia–Pacific region, and it is very important to consider the performance of QZSS-R over an oceanic area located in this region. Therefore, in the present work, the QZSS-based SNR measurement with triple-frequency signals (L1, L2, and L5) are collected. To prove the validity of QZSS-TG, we also used GPS (L1, L2, and L5) and GLONASS (L1 and L2) constellations frequency signals and estimated GPS-TG and GLONASS-TG measurements, then the reflectometry results are compared with GPS and GLONASS constellation systems. A KELM based on VMD is implemented in this study and the QZSS-TG time series are also modeled. Data collection and method modeling are described in “Data collection and method of modeling” section. Results and discussion are addressed in “Results and discussions” section and finally, the conclusions of the study have been written in “Conclusion” section.

## Data collection and method of modeling

### GNSS data collection

The Geographical Survey Institute (GSI) of Japan established a GNSS network known as GNSS Earth Observation Network (GEONET) is utilized for the current study to examine the oceanic activities in the selected region of Japan. The GEONET consists of more than 1,300 ground stations where their GNSS sites are primarily mounted on the bedrocks for high-precision crustal measurements. Ansari and Bae^25^ discussed the sea-level change around the Japanese coast by using six-tide stations by using wavelet analysis. They noticed that only the P109 site showed relative TG velocity (mm/yr) and other sites showed positive relative TG velocity (mm/yr), leading to a sample of a discrepancy between the estimated values. The negative relative velocity at the P109 site indicates subsidence. Hence, in the current study to check the applicability of QZSS-R, the GEONET data is collected from the P109 site (37.815° N; 138.281° E; 44.70 m elevation of ellipsoidal height) located in Sado Island of Japan during 01 October 2019 to 31 December 2019. Then SNR measurements with QZSS satellites are retrieved to investigate the sea-level changes in the Japanese Sea.

**Figure 2:**
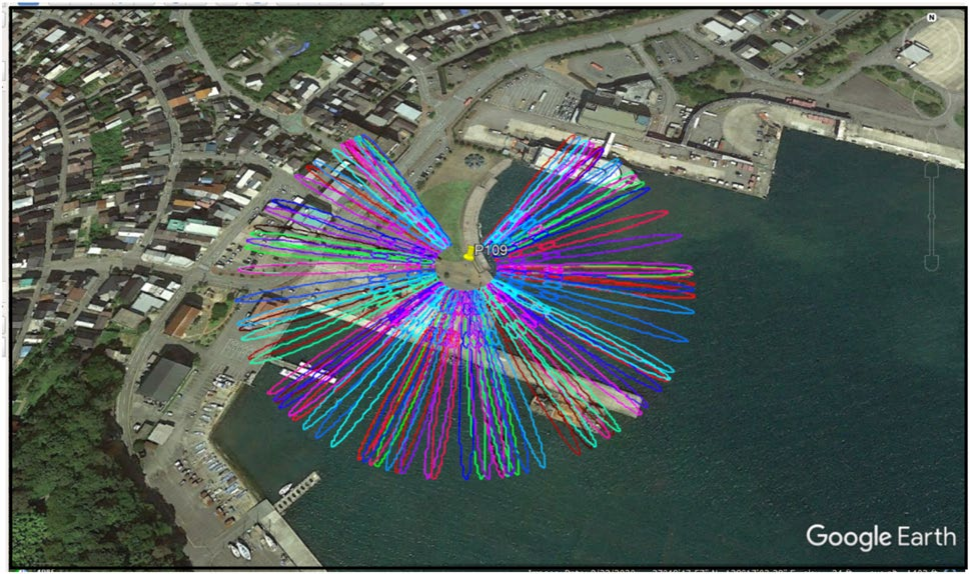
Screenshots of first Fresnel zones for the P109 site with elevation angles of 5° to 25° and azimuth range 30° to 330° projected on a Google Earth map.

According to the theory of the Fresnel zone, the reflected signal received by the GNSS antenna in the first Fresnel zone can obtain the maximum signal energy of the dielectric layer. On one side, the elliptical area of the Fresnel zone is affected by the elevation angle of the satellite and on another side, the oscillation amplitude of SNR data decreases with increasing elevation angle due to the antenna gain pattern i.e., the multipath effect is more apparent at low elevation angles. It is important to choose the range of elevation and azimuth angles to range for the SNR data^26^. Therefore with the spatial domain distribution, we selected, the first Fresnel zones of the P109 site with the azimuth range of 30° to 330° projected on a Google Earth map as shown in Fig. 2. Here, the considered first Fresnel zones are built using the GNSS-R Software as suggested by Roesler and Larson^26^. Further detailed information about the first Fresnel zones can be found in Nievinski et al.^27^. As shown in Fig. 2, the higher elevation angles include the land reflection which will not be useful to determine sea level changes. Hence in this paper, observations are used for the elevation angle within the range of 5°–25° degrees because they are easily affected by the multipath at this station. Furthermore, since the sea level change is focused on in this paper, the signals reflected from the sea surface are used. Through evaluating the map around the station and the reliability of the observations, tracks just between the azimuths of 20° to 80° and 110° to 170° are used to perform the spectral analysis.

The QZSS-TG measurement based on the SNR measurement (*dB-Hz*) and the prior reflector height (*H*_*0*_) can be calculated as follow^28^:

1 $$SNR = A\cos \left( {\frac{4\pi }{\lambda }H_{0} \;\sin \theta + \phi } \right),$$

where *A* is the amplitude that is deviated from zero (unit: m), *ϕ* is the phase offset, *θ* is the elevation angle (unit: radian), and *λ* is the signal wavelength (unit: m). A detailed study of the whole analytical solution can be found in our previous work^2^. Furthermore, the Lomb–Scargle periodogram (LSP) is used to estimate the dominant frequency or *f*_*m,t*_ (no unit) as proposed by Press et al.^29^, and the *f*_*m,t*_ is related to the effective reflector height (*H*_*eff*_) with the following equation:

2 $$f_{m,t} = \frac{{2H_{eff} }}{\lambda }$$

Here, the unit of reflector height (*H*_*eff*_) can be shown in meter (m) using the product between the *f*_*m,t*_ and the *λ.*

### GSI data collection

**Figure 3:**
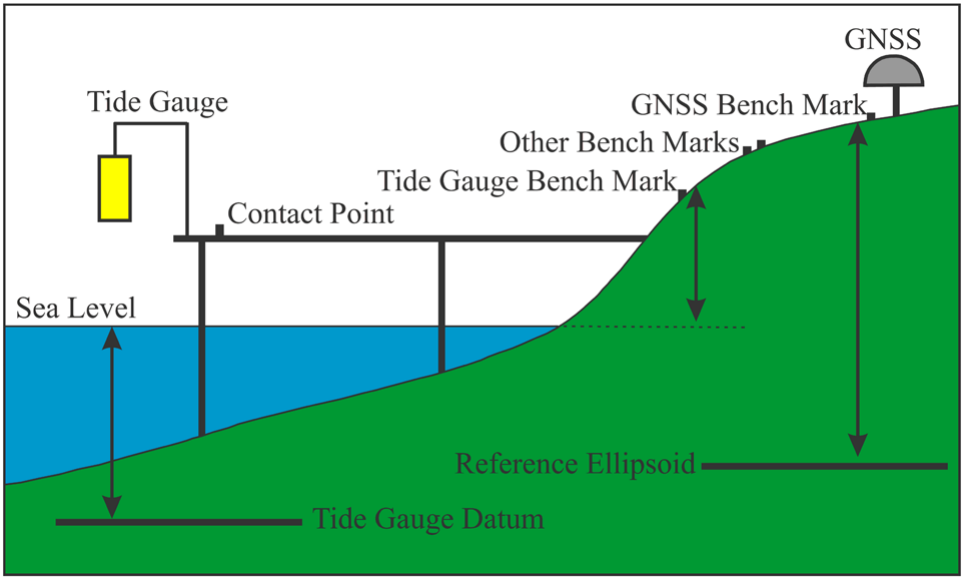
The structure of Japan tide observation station at GSI along the coastal area to continuously monitor the sea level.

There are five common types of tide gauges which are responsible for measuring tide. The floating mechanical tide gauges, floater of an encoder, pressure sensor, acoustic tide gauge, and radar sensor^30^. The gauge employs a mechanical system, working with a floating unit that is placed in a stilling well connected directly to the sea where the sea water enters into it^31^. At the beginning of the twenty-first century, as a result of the increasing interest in high-quality sea level data, tide gauge networks have been upgraded and traditional mechanical float tide gauges have been progressively substituted by electronic gauges provided with a floater of the encoder, acoustic, pressure and recently radar sensors^32^. The GSI of Japan has installed approximately 25 tide observation stations along their coastal regions to regularly monitor the sea-level changes. The GSI web can be reached via https://www.gsi.go.jp/kanshi/tide_furnish.html. The structure of the Japan TG observation station at GSI along the coastal area is shown in Fig. 3, where the sea-level changes are continuously monitored. The GSI-TG measurements are measured in height (unit: m) from the reference plane observations of the value of measured sea levels always have a positive sign. This observed height is called the observed sea level, then they are translated to Tokyo Peil (TP) which is the mean sea level at Tokyo Bay. The GSI-TG measurement is obtained from the same co-located station for the validation objective. The method of calculation of the sea level observation to the TP converted tide level is as follows (https://www.gsi.go.jp/kanshi/tide_comment.html):

3 $${\text{Sea}}\,{\text{Level}}\,{\text{from}}\,{\text{TP}} = {\text{Observed}}\,{\text{Sea}}\,{\text{Level}} + {\text{Height}}\,{\text{of}}\,{\text{the}}\,{\text{fixed}}\,{\text{point}} - {\text{Datums}}\,{\text{constant}}$$

The datum constant value in Eq. (3) depends upon the location of the GSI station.

### KELM modeling

Let us consider *N* arbitrary samples of (x_i_, y_i_) where x_i_ ɛ ℜ^N^ and y_i_ ɛ ℜ^N^ (i = 1, 2, …, *N*), the activation function of the hidden layer is given by *h*(*x*), and *Y* is defined as an output matrix. The single-hidden layer feedforward neural networks (SLFN) can be shown as follows^33^:

4 $$Y = \left[ {\begin{array}{*{20}c} {y_{1j} } \\ {y_{2j} } \\ . \\ . \\ . \\ {y_{mj} } \\ \end{array} } \right]_{m \times N} = \left[ {\begin{array}{*{20}c} {\sum\limits_{i = 1}^{l} {\alpha_{il} h\left( {w_{i} x_{j} + k_{i} } \right)} } \\ {\sum\limits_{i = 1}^{l} {\alpha_{il} h\left( {w_{i} x_{j} + k_{i} } \right)} } \\ . \\ . \\ . \\ {\sum\limits_{i = 1}^{l} {\alpha_{il} h\left( {w_{i} x_{j} + k_{i} } \right)} } \\ \end{array} } \right]_{m \times N}$$

where *α* is the output weights between the output layers and the hidden layers, *w*_*i*_ = [*ω*_*i*1_, *ω*_*i*2_, …, *ω*_*iN*_]^T^ presents the input weights between the input layers and the *i*th hidden layers. The number of the hidden nodes and the threshold of the hidden layer is represented by *l* and *a*, respectively. The k_i_ are the threshold of the hidden layer. Now, Eq. (4) can be written in another form:

5 $$\begin{gathered} H\alpha = Y,\;\;Y \in \Re^{N \times m} ,\;\;\alpha \in \Re^{N \times m} ,\;\; \hfill \\ H = H\left( {w,a} \right) = h\left( {wx + a} \right). \hfill \\ \end{gathered}$$

Here the output matrix of the hidden layer is represented by *H*, and *α* is an unknown parameter estimated by the least square’s method. By taking Moore–Penrose generalized inverse matrix, the solution of Eq. (5) can be written as^34^:

6 $$\alpha = H^{\dag } Y,H^{\dag } = H^{T} \left( {HH^{T} } \right)^{ - 1}$$

where *H*^†^ presents the Moore–Penrose generalized inverse matrix of *H*^35,36^. Different kinds of methods can be applied to estimate the generalized Moore–Penrose inverse of a matrix such as the orthogonalization method, orthogonal projection method, singular value decomposition method, and iterative method. The method of orthogonal projection can be applied in two distinct cases, first when H^T^H is non-singular and $H^{\dag } = \left( {H^{T} H} \right)^{ - 1} H^{T} \;$ or secondly when H^T^H is non-singular and $H^{\dag } = H^{T} \left( {HH^{T} } \right)^{ - 1}$^20,35^.

If we add a decisive penalty factor 1/F in Eq. (6), the resultant solution will be stable and have a tendency for better generalization of performance. The value of α will be given like this:

7 $$\alpha = H^{T} \left( {1/F + HH^{T} } \right)^{ - 1} Y\;$$

The orthogonal projection method α can be estimated by using the Ridge regression theory^37^. The output function expression for the extreme learning machine (ELM) will be converted as:

8 $$f(x) = H\alpha = HH^{T} \left( {1/F + HH^{T} } \right)^{ - 1} Y\;$$

Now, the hidden layer h(x) activation function can be substituted with the kernel function in terms of Mercer's conditions^38^, and the KELM output function can be given in the following equation^33^:

9 $$f(x) = h(x)\hat{\alpha } = \left[ {\begin{array}{*{20}c} {k\left( {x,x_{1} } \right)} \\ {k\left( {x,x_{1} } \right)} \\ . \\ . \\ . \\ {k\left( {x,x_{1} } \right)} \\ \end{array} } \right]\left( {1/F + HH^{T} } \right)^{ - 1} Y$$

In Eq. (9), the random mapping of the ELM is substituted with the kernel function, hence, the output weights become more stable.

The VMD algorithm establishes a constrained optimization problem based on the assumption that all components are narrowband signals concentrated near their respective center frequencies and decomposes the original signal into intrinsic mode functions (IMFs) with a certain number of layers at different frequencies^39^

10 $$X(t) = \sum\limits_{s = 1}^{S} {u_{s} (t) + r(t)}$$

where *X *(*t*) is the original signal, *u*_*s*_(*t*) is the *s*th IMF component, *r*_*n*_(*t*) is the residual term and *S* is the number of decomposition levels. The IMF is amplitude modulation and frequency modulation signal, written as^40^:

11 $$u_{s} (t) = A_{s} (t)\cos (\phi_{s} (t))$$

This is notable, although the superposition of all sub-signals u_k_ is the SNR, the phase φ_s_ of the sth sub-signal is not directly related to φ_γ_ in the SNR. The IMF that is highly consistent with the original SNR is selected as the trend term, and the remaining IMFs can be reconstructed to obtain the oscillation term^41^. The VMD algorithm only replaces polynomial fitting here, and it is still necessary that LSP spectral analysis is used to extract the frequency of the oscillation term. The advantage of the VMD algorithm for fitting the trend term is that it has similar characteristics to the adaptive high-pass filter, which separates high- and low-frequency components to achieve detrend processing^41^.

## Results and discussions

### QZSS-TG measurement analysis

**Figure 4:**
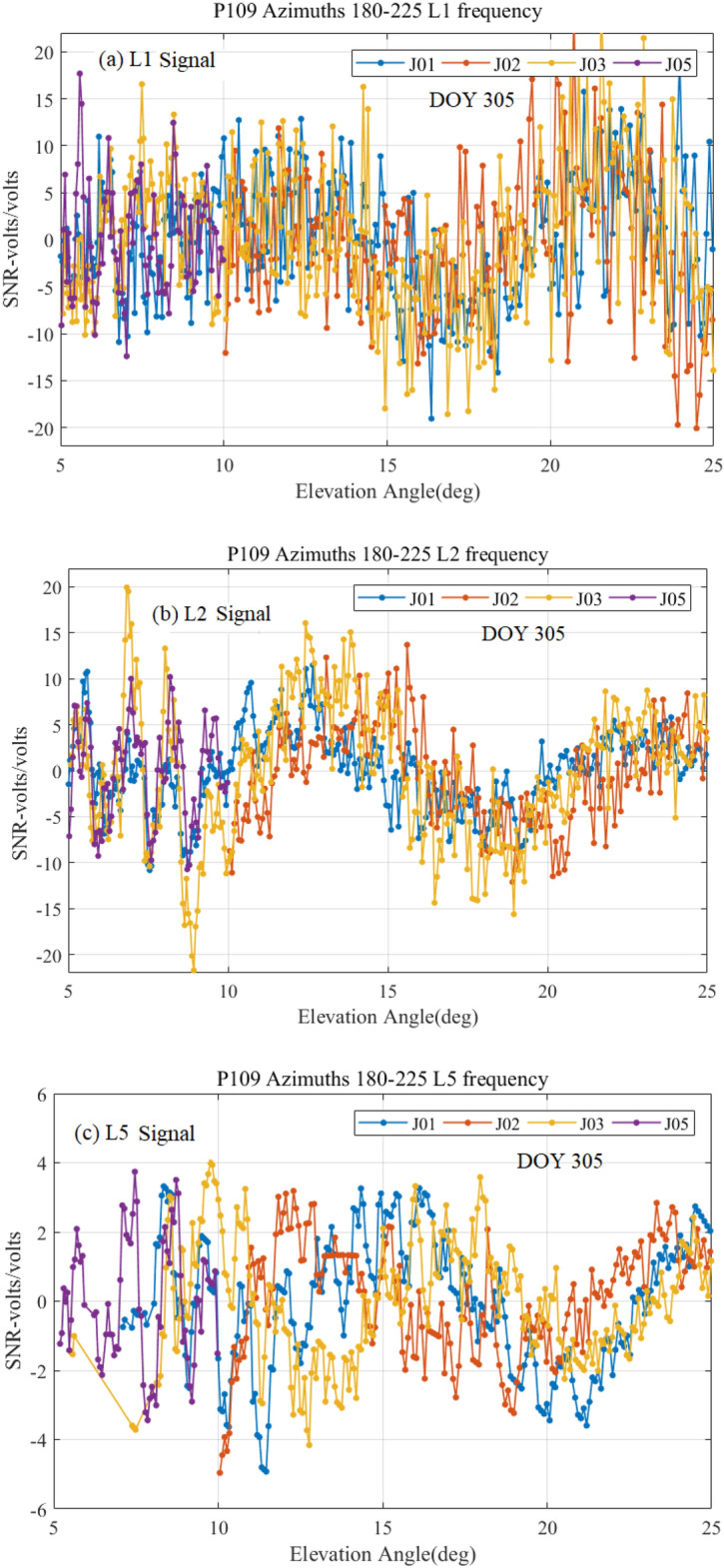
SNR (volts/volts) observation plots with respect to the elevation angles for (**a**) L1 signal, (**b**) L2 signal, and (**c**) L5 signal on the day of November 2019 (DOY 305).

The interferences between the reflected and the directed QZSS signals were recorded by SNR interferograms. In order to obtain the error in multipath, a low-order polynomial (second degree) is utilized to attain SNR detrended time series^2,9^. After removing the SNR trend, SNR plots (linear scale units of volts/volts) against the elevation angles are studied. Hereafter, the unit “linear scale unit of volts/volts” of SNR is shown shortly in terms of “volts/volts” throughout this paper. A typical example of J01, J02, J03, and J05 (this is official PRN notation of QZSS satellites like QZS-1, QZS-2, QZS-3 and QZS-4) satellites with triple frequencies of L1, L2, and L5 signals on the DOY 305 of November 2019 has been shown in Fig. 4. Here the P0109 site of GNSS Earth Observation Network in Japan (37.815° N; 138.281° E; 44.70 m elevation in elevation height with reference to ITRF14) consists of standard geodetic quality choke ring zenith pointing antenna TRM29659.00 (antenna height 0.83 cm) with dual-frequency carrier-phase GNSS receiver TPS NETG5 (version 5.1p2) operating at 30 samples per seconds. It is clear from Fig. 4 that the SNR observations are oscillating with the increase of elevation angle due to the multipath errors. If there are no multipath errors, the SNR measurements will smoothly rise with the increase of elevation angles^42,43^. The variation of the SNR plot shows that the strength of multipath signals varies between − 20 to 20 V/volts for L1 and L2 QZSS signals, while they are very low between − 5 to 5 V/volts for the L5 QZSS signal. The latter is caused by the lowest noise and multipath error of the L5 signal^44^. It can also be verified that before and after multipath corrections, the QZSS L5 signal provides higher precision positioning accuracy as compared to the L1 and L2 signals^45,46^. This happens due to the higher value of chipping rates (10.23 Mbps) on the L5 signal and the transmitted higher power which generates larger SNR and better rejection of multipath^47^.

**Figure 5:**
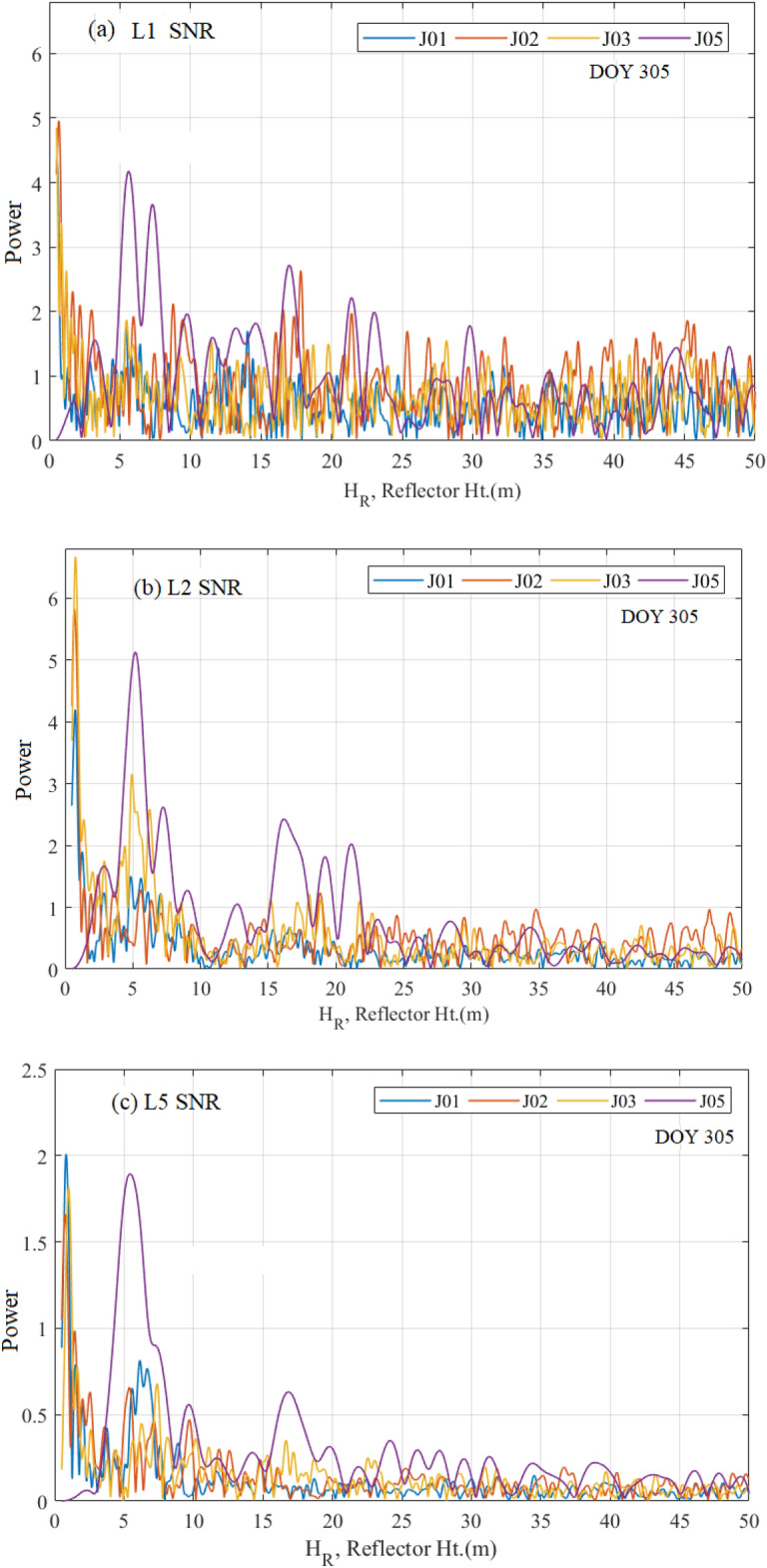
QZSS (**a**) L1 SNR, (**b**) L2 SNR, and (**c**) L5 SNR observations with multipath patterns and LSP for J01, J02, J03 and J05 satellites. Note the *H*_*eff*_ is one point on the x-axis and Ht. stands for Height.

The dominant multipath error frequency can be a result of the SNR data by using the Lomb–Scargle periodogram (LSP) or other spectrum analyses. The LSP method is used to convert the data into the frequency domain as well as to obtain the dominant frequency^25^. The dominant frequency for the SNR measurements used in Fig. 4 is converted to the effective reflector height by applying Eq. (2). Figure 5a–c show the SNR observations of QZSS L1, L2, and L5 signals, multipath patterns, and LSP for J01, J02, J03, and J05 satellites on 01 November 2019 (DOY 305). The frequencies of multipath patterns are affected unequally by multipath errors, so the minute difference in peak height of reflected sea-level changes will be obtained, therefore, the mean or median values of these heights can be considered as the final estimation of reflected height of sea-level changes. It has been observed from Fig. 5a–c that the reflector heights obtained from the L1 signal were 0.67 m, from the L2 signal was 0.59 m, and from the L5 signal was 0.73 m. Hence the mean value of these heights [(0.67 + 0.59 + 0.73)/3 = 0.663 m] will be the final estimation of the reflected height of sea-level changes. More details about the QZSS (L1, L2, and L5) signals including the GPS (L1, L2, and L5) and GLONASS (L1 and L2) will be discussed in the next section (Tables S1, S2, S3).

### Comparison with GSI-TG measurements

**Figure 6:**
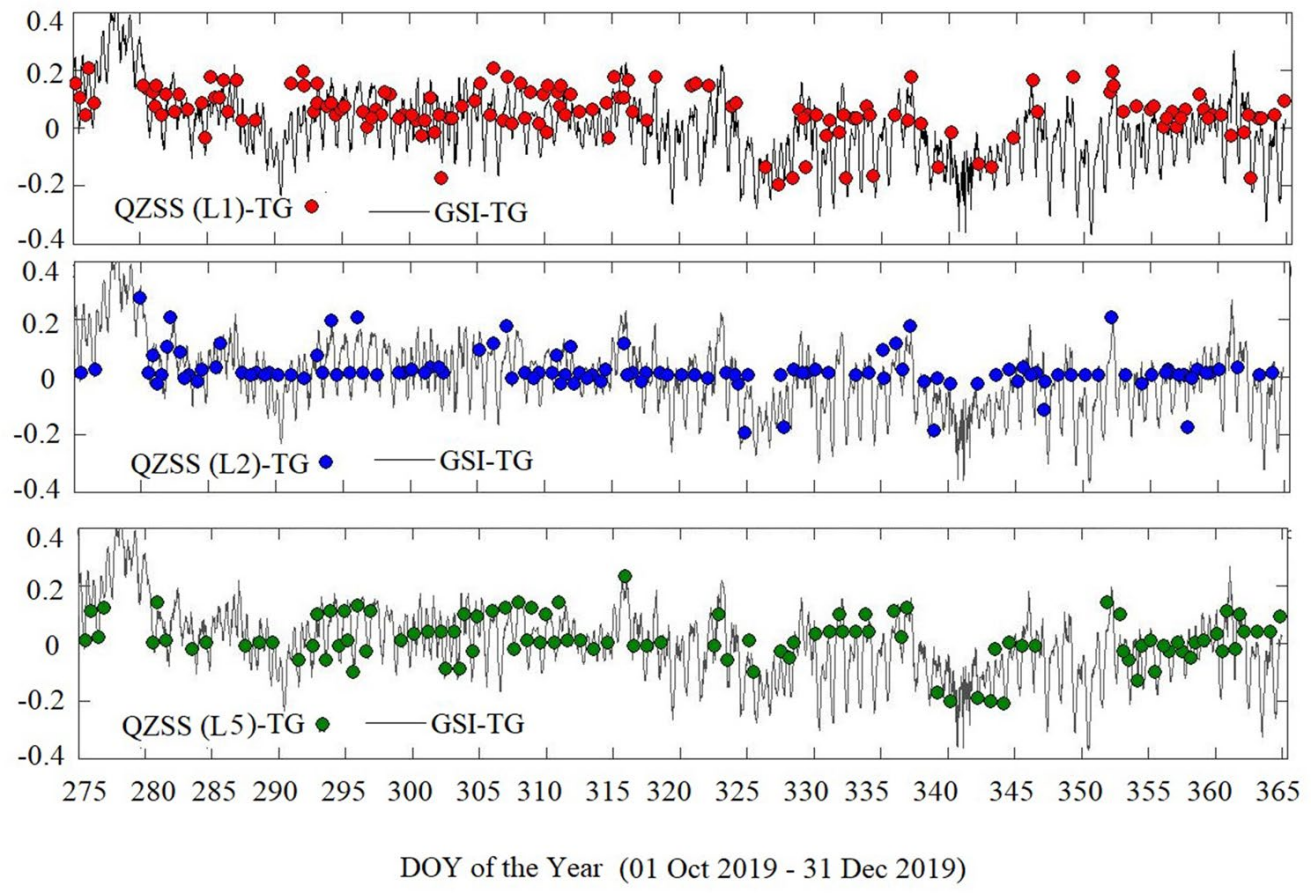
Daily sea-level changes (unit: m) obtained from QZSS L1, L2 and L5 SNR observations (namely, QZSS-TG) as well as GSI tide gauge (namely, GSI-TG) observations during 01 October 2019 to 31 December 2019 (90 days).

Daily sea-level changes (unit: m) obtained from QZSS L1, L2, and L5 SNR observations (namely, QZSS-TG), as well as GSI tide gauge (namely, GSI-TG) observations during the 01 October 2019 to 31 December 2019 (90 days), are plotted in Fig. 6 (Supplemenary file). The QZSS-TG observations for L1, L2, and L5 signals are derived and shown in Fig. 6 with red (L1 signal), blue (L2 signal), and green (L5 signal) color circles, respectively, while the GSI-TG observations are also depicted in Fig. 6 with a black solid line. The observation obtained from TG sea level will be relative to the TG benchmark while derived results of sea level changes from GNSS measurements will be relative to the GNSS stations. Therefore, a mean sea level is achieved by taking the difference between TG results and GNSS-derived observations. Sometimes mean value provided by QZSS-R showed very much different from the mean value provided by TG. This happened because of improper visibility of signals. For example, a signal is visible from 25° to 60°, and we have included elevation only to 30°. It means only data from 25° to 30° will be used for analysis. Such types of signals provide error. Hence, we have ignored them by using some possible limited differences. To care about the above points, first, we roughly estimated the height of the water surface, which lies in the range of 0.5–1.5 m. It is a height filter, and the MATLAB code will print only those values of water surface which lie between this range. Other unexpected heights will be excluded automatically.

The number of reflection points tracked over land will be reflected from the land surface and generated by the code as a zero value. As above we already mentioned the threshold range (0.5 to 1.5 m) hence the reflection on land will be excluded automatically. The number of reflection tracks used for retrieval from the different systems QZSS, GPS, and GLONASS was counted as shown in Table 1. The points which were lies in the range of 0.5–1.5 m were included and the other point beyond of range were excluded (Table 1). As we can see from the table the total number of points observed by the GPS constellation was high compared to GLONASS and QZSS constellations. This happened because more GPS satellites are visible in this region compared to others. Since the QZSS constellation has only four satellites hence their recorded data points are the lowest. Although the QZSS constellation has the lowest number of observed and included points, still the constellation is able to provide good accuracy of sea-level measurements and can be used for QZSS-reflectometry, which is the main aim of the current study. In Fig. 6, the observation dates are shown by the horizontal axis, while both QZSS-TG and GSI-TG observations are presented by the vertical axis. There is a significant role of daily periodicity in sea level (black solid line) can be seen in Fig. 6, which is possibly caused by wind and tide variability, as well as the variability of sea level pressure^48^. We can also see from Fig. 6 that all of the L1, L2, and L5 signals for QZSS-TG observations are not uniformly distributed. The feasibly main reasons are the visibility of QZSS satellites over a certain period of time which becomes a cause of non-availability of observation over a quite long-time interval. The good agreements between GSI-TG and QZSS-TG estimates over a period of 90 days are clearly visible in Fig. 6. The correlation coefficients and root mean square errors (RMSEs) of QZSS-TG, GPS-TG, and GLONASS-TG observations have been listed in Table 2. It is visible from Table 2 that the correlation coefficient of QZSS L1, L2, and L5 signals are equal to 0.8712, 0.6998, and 0.8763, respectively and their RMSEs are equal to 0.0484 m, 0.0426 m, and 0.0424 m, respectively. These values are almost equivalent to GPS-TG and GLONASS-TG values as shown in Table 2. In general, the number of GPS and GLONASS satellites are the largest compared to QZSS so, they should have the average highest temporal resolution estimation for sea-level changes. This means QZSS-Reflectometry is able to provide a new chance to monitor sea-level changes with the available frequencies and the proposed technique.

**Table 1:** Total number of points by different constellation, only those points which has range value 0.5 to 1.5 are included and others are excluded.

Constellation	Total number of pointes	Included points (Nov + Oct + Dec)	Excluded points
QZSS (L1)	369	38 + 47 + 42 = 127	242
QZSS (L2)	333	40 + 35 + 45 = 120	210
QZSS (L5)	264	30 + 34 + 38 = 102	162
GPS (L1)	4074	384 + 326 + 325 = 1035	3039
GPS (L2)	3276	237 + 249 + 233 = 719	2557
GPS (L5)	927	93 + 94 + 81 = 268	659
GLONASS (L1)	2373	236 + 239 + 247 = 722	1751
GLONASS (L2)	1956	170 + 155 + 175 = 500	1456

**Table 2:** Correlation coefficient and root mean square (RMSE) between observed GSI-TG and GNSS (QZSS, GPS and GLONASS) estimated TG.

Constellation	Tested signals	Correlation	RMSE (m)
QZSS-TG	L1	0.8711	0.0484
	L2	0.6996	0.0426
	L5	0.8762	0.0424
GPS-TG	L1	0.8842	0.0491
	L2	0.8970	0.0484
	L5	0.7472	0.0450
GLONASS-TG	L1	0.8371	0.0475
	L2	0.9012	0.0494

### Implication of KELM modeling

**Figure 7:**
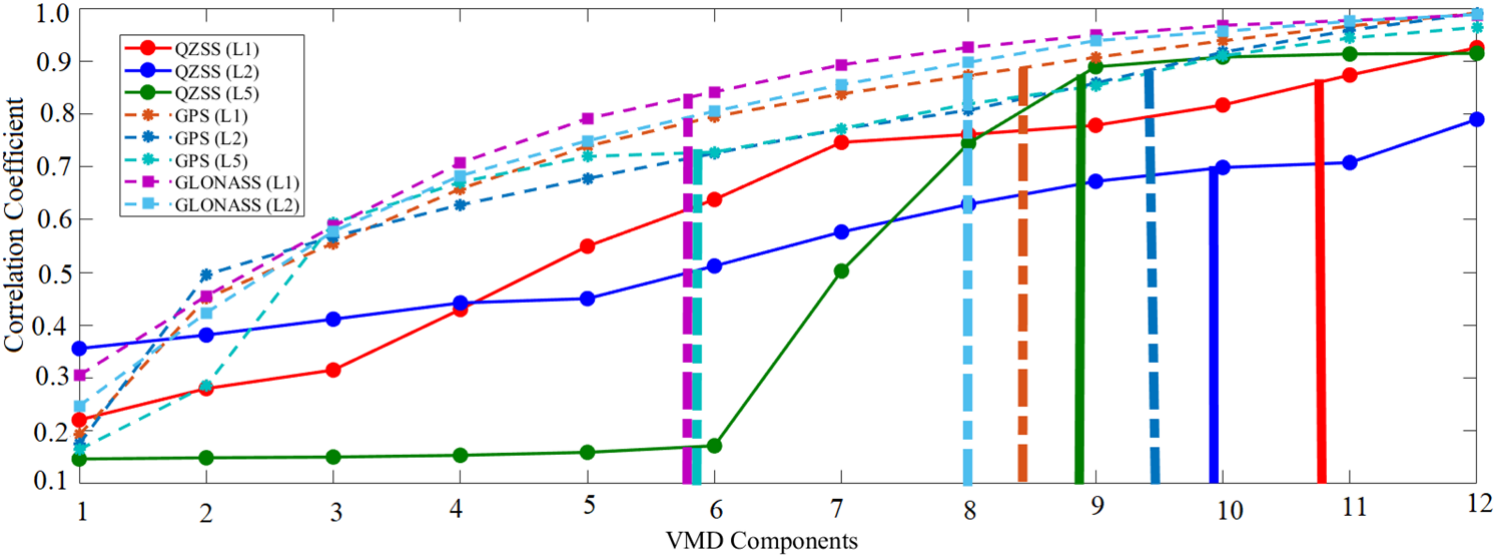
Correlation coefficients of QZSS (L1, L2 and L5), GPS (L1, L2 and L5) and GLONASS (L1 and L2) signals after implementing KELM modeling with 1–12 VMD components.

To improve the QZSS-R-based measurements and reconstruct the tidal variability, we implicate the KELM modeling method. The number of VMD components varying from 1 to 12 are used to predict the best possible results. Figure 7 shows the correlation coefficients for QZSS (L1, L2, and L5 signals), GPS (L1, L2, and L5 signals), and GLONASS (L1 and L2 signals) after implementing KELM from 1 to 12 VMD components. The vertical lines in Fig. 7 correspond to the correlation coefficients taken from Table 2. It can be seen obviously that the performance of the correlation coefficient is enhanced significantly at a particular high VMD component as compared to the low VMD component. This indicates that before implementing KELM, we need to take care of a proper VMD component. The 12th VMD component showing the best performance of correlation coefficient and RMSE have been presented in Tables 3 and 4, respectively. Moreover, it can be seen from Fig. 7 that KELM modeling for QZSS signals provides a nice improvement in terms of the correlation coefficient, for example, QZSS L1 of 0.9252, QZSS L2 of 0.7895, and QZSS L5 of 0.9146. However, the correlation improvements are not higher than GPS with an average of 0.9816 as well as GLONASS with an average of 0.9877. This indicates that the KELM model works in a proper way, but the nice improvements in QZSS measurement are less because of its high variability. Although the correlation coefficients of QZSS measurement are slightly degraded as compared to those of GPS and GLONASS measurements, the correlation coefficients are increased significantly.

**Table 3:** GNSS measurement correlation coefficient and their improvement after KELM implication.

Constellation	Tested signals	Correlation		Improvement in correlation
		GNSS (without KELM) vs GSI	GNSS (without KELM) vs GNSS (with KELM)	
QZSS-TG	L1	0.8711	0.9252	0.0541
	L2	0.6996	0.7895	0.0899
	L5	0.8762	0.9146	0.0384
GPS-TG	L1	0.8842	0.9908	0.1066
	L2	0.8970	0.9904	0.0924
	L5	0.7472	0.9637	0.2165
GLONASS-TG	L1	0.8371	0.9874	0.1503
	L2	0.9012	0.9879	0.0867

**Table 4:** GNSS measurement RMSE and their improvement after KELM implication.

Constellation	Tested signals	RMSE (m)		Improvement in RMSE (m)
		GNSS (without KELM) vs GSI	GNSS (without KELM) vs GNSS (with KELM)	
QZSS-TG	L1	0.0484	0.0404	0.0080
	L2	0.0426	0.0385	0.0041
	L5	0.0424	0.0392	0.0032
GPS-TG	L1	0.0491	0.0410	0.0081
	L2	0.0484	0.0400	0.0044
	L5	0.0450	0.0390	0.0060
GLONASS-TG	L1	0.0475	0.0414	0.0061
	L2	0.0494	0.0413	0.0081

**Figure 8:**
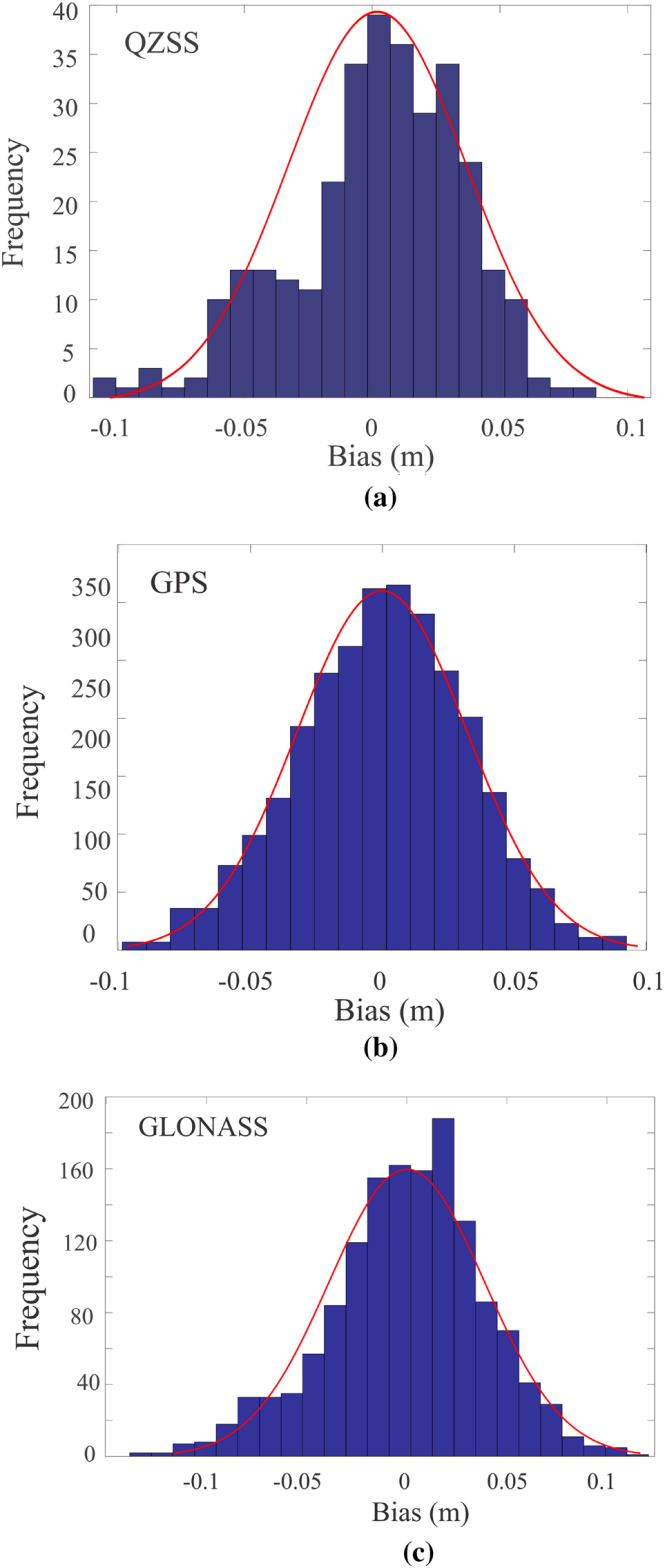
The error histograms between the satellite systems (**a**) QZSS-R, (**b**) GPS-R and (**c**) GLONASS-R and KELM reconstructed Tide gauge measurements.

For more analysis, we plotted the error histograms between the satellite systems (i.e., QZSS-R, GPS-R, and GLONASS-R) and KELM reconstructed TG results as shown in Fig. 8. It can be seen manifestly that the errors behave approximately normal distributions. The mean errors for QZSS-R, GPS-R, and GLONASS-R are estimated at − 2.0 mm, − 0.13 mm, and 0.02 mm, respectively, and the respective standard deviations are 0.0781 m, 0.0323 m, and 0.0389 m, respectively. Here, the mean errors are very close to zero or equivalent to zero mean, therefore according to the standardized normal distribution the estimated results can be considered as unbiased estimations. Finally, we can infer that KELM reconstructed TG errors are significantly reduced, and their distributions are more reasonable. Because the KELM modeling shows good agreements between the reconstructed and measured tidal variability, KELM is thus suitable to reconstruct the tidal variability for QZSS-R measurements with enhanced correlation coefficients.

## Conclusion

In this paper, QZSS SNR data with triple frequencies of L1, L2, and L5 signals were firstly used to estimate sea-level changes at the P109 site of the Japanese GNSS Earth Observation Network (37.815° N; 138.281° E; 44.70 m elevation height) located in Sado Island of Japan. To compare with the tide gauge measurement of QZSS-R, other GNSS-R data with different frequency bands were also used to estimate the TG such as GPS (L1, L2, L5 signals) and GLONASS (L1 and L2 signals). Initial sea-level estimates were obtained through Lomb–Scargle periodogram (LSP) spectral analysis on the detrended SNR time series. A KELM technique based on VMD was applied to the QZSS, GPS, and GLONASS observations to reconstruct their tidal variability. The results show good agreements between observed and KELM-estimated TG obtained from QZSS, GPS, and GLONASS data in terms of correlation coefficients and root mean square errors. The research findings demonstrate that the refined sea-level estimates based on KELM achieve significant accuracies in terms of correlation coefficients and histogram plots of errors. The estimated tide gauges obtained from QZSS measurements are comparable to those of GPS and GLONASS measurements. Furthermore, since wave dynamics of water level near the coastal areas in storm situations are very important to make research and discuss additionally, the future study will focus on proving the KELM modeling with other constellations such as Galileo and BeiDou, as well as including more stations and longer data.

## Supplementary Information

Supplementary Information.

## Data Availability

All data generated or analyzed during this study are included in this published article [and its supplementary information files].

## Abbreviations

QZSS-R: Quasi zenith satellite system-reflectometry
GPS-R: Global positioning system-reflectometry
GNSS-R: Global navigation satellite system-reflectometry
GLONASS-R: Globalnaya navigatsionnaya sputnikovaya sistema-reflectometry
KELM: Kernel extreme learning machine
GEONET: GNSS Earth Observation Network
QZO: Quasi-zenith orbits
LSTM: Long short-term memory
VMD: Variational mode decomposition
SLFN: Single-hidden layer feedforward neural networks
LSP: Lomb–Scargle periodogram
TP: Tokyo Peil
