# Publisher Correction: Quasi zenith satellite system-reflectometry for sea-level measurement and implication of machine learning methodology

**DOI:** 10.1038/s41598-023-27691-4

**Published:** 2023-01-11

**Authors:** Kutubuddin Ansari, Hong‑Woo Seok, Punyawi Jamjareegulgarn

**Affiliations:** 1 Integrated Geoinformation (IntGeo) Solution Private Limited, New Delhi, India; 2 Department of Geoinformatic Engineering, Inha University, Incheon, South Korea; 3 Space Technology Development Center, KMITL, Prince of Chumphon Campus, Chumphon, Thailand

Correction to: *Scientific Reports* 10.1038/s41598-022-25994-6, published online 12 December 2022

In the original version of this Article a previous rendition of Figure 6 was published. The original Figure 6 and accompanying legend appear below.

**Figure 6:**
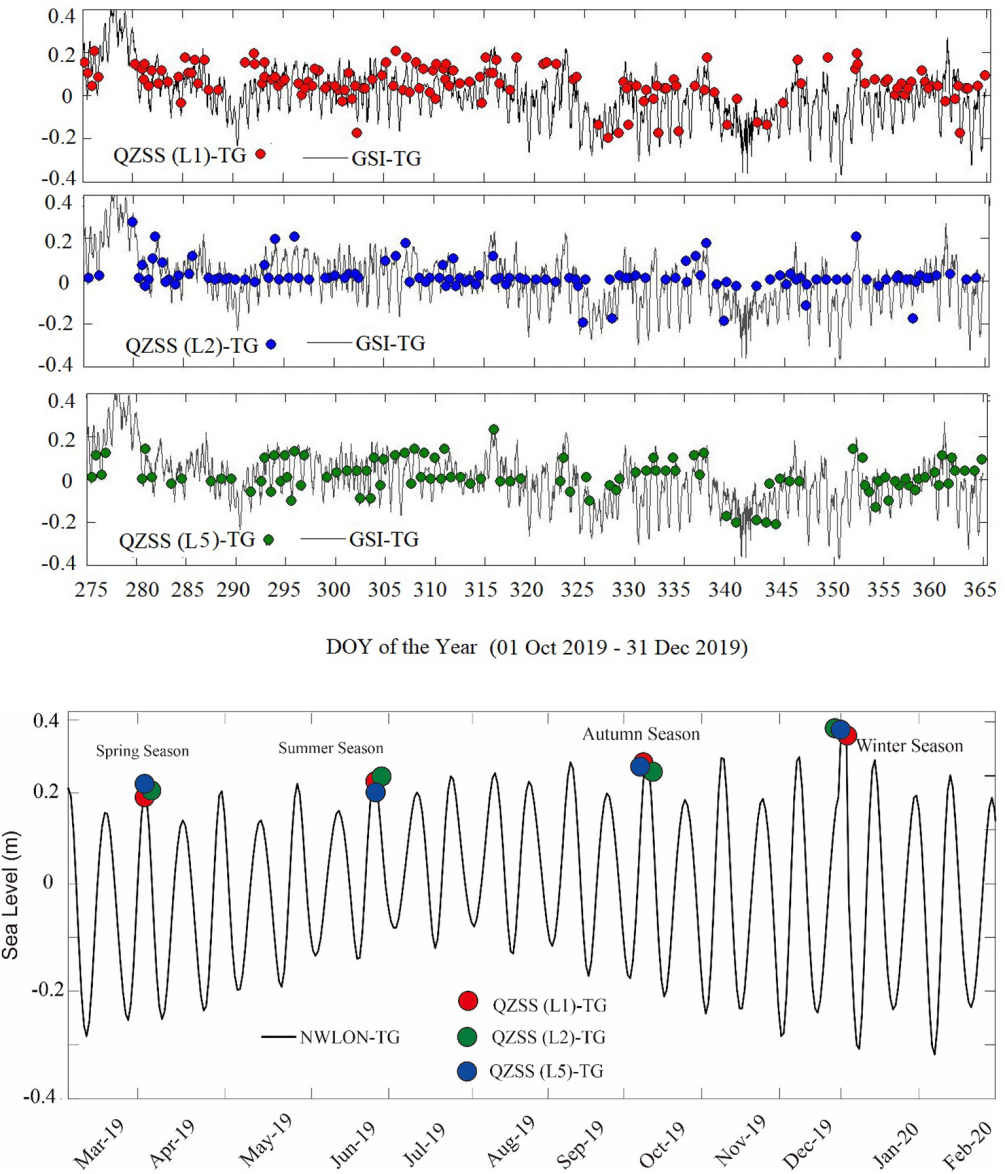
Daily sea-level changes (unit: m) obtained from QZSS L1, L2 and L5 SNR observations (namely, QZSS-TG) as well as GSI tide gauge (namely, GSI-TG) observations during 01 October 2019 to 31 December 2019 (90 days).

The original Article has been corrected.

