# Supplementary material for: Quasi zenith satellite system-reflectometry for sea-level measurement and implication of machine learning methodology
Source: Sci Rep. 2022 Dec 12;12:21445. doi: 10.1038/s41598-022-25994-6 (PMC9744832; doi:10.1038/s41598-022-25994-6)
Supplement: Supplementary file 1 — Supplementary Information. [file 41598_2022_25994_MOESM1_ESM.docx]

| Table S1: We studied QZSS (L1, L2 and L5) derived tide gauge (TG) measurements for the current study. In this table, column 1 indicates time in hours from 01 October 2019 to 31 December 2019. Column 2, the QZSS (L1) signal derived results which is subtracted from its mean. Column 3 indicates corresponding GSI-TG values, from which its mean value is subtracted. Similarly, column 4 time in hours, column 5 is QZSS (L2) signal derived results and column 6 is GSI-TG. In the same way, column 7 is again time in hours, column 8 is QZSS (L5) signal derived results and column 6 is GSI-TG. | | | | | | | | | | | |
| --- | --- | --- | --- | --- | --- | --- | --- | --- | --- | --- | --- |
| Time in Hours from 01 October 2019 | QZSS-TG  (L1)  (Mean-Derived) | GSI-TG  (Derived -Mean) |  | Time in Hours from 01 October 2019 | QZSS-TG  (L1)  (Mean-Derived) | GSI-TG  (Derived -Mean) |  | Time in Hours from 01 October 2019 | QZSS-TG  (L5)  (Mean-Derived) | | GSI-TG  (Derived -Mean) |
| 13.3 | 0.1055 | 0.1059 |  | 37.2 | 0.0239 | 0.0569 |  | 21.9 | | 0.1123 | 0.1039 |
| 21.9 | 0.0455 | 0.0949 |  | 117.6 | 0.2739 | 0.3109 |  | 37.1 | | 0.0223 | 0.0439 |
| 4.6 | 0.1555 | 0.2259 |  | 133 | 0.0139 | 0.0559 |  | 45.8 | | 0.1223 | 0.1539 |
| 37.2 | 0.0855 | 0.0569 |  | 141.6 | 0.0739 | 0.0879 |  | 132.8 | | 0.0023 | 0.0609 |
| 28.5 | 0.2055 | 0.2219 |  | 156.9 | 0.0039 | 0.0459 |  | 141.5 | | 0.1423 | 0.0849 |
| 141.6 | 0.1255 | 0.0879 |  | 165.5 | 0.1039 | 0.0189 |  | 156.7 | | 0.0123 | 0.0179 |
| 147 | 0.0755 | 0.0879 |  | 170.9 | 0.2039 | 0.1509 |  | 228.5 | | 0.0023 | 0.0419 |
| 156.9 | 0.0455 | 0.0459 |  | 189.4 | 0.0839 | 0.0589 |  | 300.3 | | -0.0077 | 0.0529 |
| 189.4 | 0.1155 | 0.0589 |  | 204.8 | 0.0039 | 0.0459 |  | 324.2 | | 0.0023 | -0.0011 |
| 204.8 | 0.0655 | 0.0459 |  | 196.1 | -0.0061 | 0.0439 |  | 396 | | -0.0577 | -0.1041 |
| 234.3 | -0.0345 | -0.0881 |  | 228.7 | 0.0239 | 0.0299 |  | 428.7 | | 0.1023 | 0.0629 |
| 228.7 | 0.0855 | 0.0299 |  | 220 | -0.0161 | 0.0379 |  | 443.9 | | -0.0577 | -0.0321 |
| 252.6 | 0.1055 | 0.1209 |  | 300.5 | 0.0139 | 0.0259 |  | 452.6 | | 0.1123 | 0.1149 |
| 267.9 | 0.1655 | 0.1139 |  | 324.4 | 0.0139 | 0.0079 |  | 467.8 | | -0.0077 | -0.0371 |
| 300.5 | 0.0255 | 0.0259 |  | 315.7 | 0.0039 | -0.0021 |  | 476.6 | | 0.1123 | 0.0879 |
| 291.8 | 0.1655 | 0.1409 |  | 348.4 | 0.0139 | -0.0621 |  | 500.5 | | 0.1323 | 0.0959 |
| 324.4 | 0.0255 | 0.0079 |  | 339.7 | 0.0039 | 0.0479 |  | 524.4 | | 0.1123 | 0.0569 |
| 428.8 | 0.0555 | 0.0559 |  | 363.6 | 0.0039 | -0.0611 |  | 578.9 | | 0.0123 | 0.0459 |
| 411.5 | 0.1455 | 0.0869 |  | 387.5 | 0.0039 | 0.0569 |  | 602.8 | | 0.0323 | 0.0439 |
| 434.2 | 0.0855 | 0.0969 |  | 434.2 | 0.0739 | 0.0969 |  | 659.3 | | -0.0877 | -0.0301 |
| 452.7 | 0.0755 | 0.1279 |  | 444.1 | 0.0139 | -0.0681 |  | 650.7 | | 0.0423 | 0.0679 |
| 435.4 | 0.1555 | 0.1299 |  | 458.1 | 0.1939 | 0.1179 |  | 683.3 | | -0.0877 | -0.0331 |
| 476.6 | 0.0655 | 0.1059 |  | 468 | 0.0039 | -0.0431 |  | 692 | | 0.1023 | 0.0479 |
| 483.3 | 0.0755 | 0.1239 |  | 491.9 | 0.0139 | -0.0461 |  | 674.6 | | 0.0423 | 0.0799 |
| 515.9 | 0.0555 | 0.0469 |  | 506 | 0.2039 | 0.1199 |  | 715.9 | | 0.0923 | 0.0529 |
| 524.5 | 0.0055 | 0.0389 |  | 515.9 | 0.0139 | 0.0469 |  | 741.9 | | 0.1123 | 0.0909 |
| 539.8 | 0.0655 | 0.0739 |  | 539.8 | 0.0039 | 0.0739 |  | 781 | | -0.0177 | -0.0401 |
| 548.4 | 0.0455 | 0.0099 |  | 587.7 | 0.0139 | 0.0929 |  | 789.7 | | 0.1423 | 0.1749 |
| 563.7 | 0.1155 | 0.1239 |  | 579 | 0.0139 | 0.0379 |  | 828.9 | | 0.0023 | -0.0481 |
| 555.1 | 0.1255 | 0.1009 |  | 603 | 0.0239 | 0.0729 |  | 852.8 | | 0.0023 | 0.0109 |
| 587.7 | 0.0455 | 0.0929 |  | 635.5 | 0.0339 | 0.0879 |  | 900.6 | | 0.0123 | 0.0149 |
| 579 | 0.0355 | 0.0379 |  | 659.5 | 0.0139 | -0.0141 |  | 948.5 | | 0.0023 | 0.0289 |
| 620.2 | -0.0245 | -0.0211 |  | 650.8 | 0.0339 | 0.0709 |  | 1020.3 | | -0.0077 | -0.0461 |
| 603 | 0.0455 | 0.0729 |  | 723.4 | 0.0939 | 0.1609 |  | 1148.7 | | 0.1023 | 0.1119 |
| 635.5 | 0.1055 | 0.0879 |  | 747.3 | 0.1139 | 0.1159 |  | 1211.8 | | -0.0977 | -0.1531 |
| 644.2 | -0.0145 | -0.0531 |  | 771.2 | 0.1739 | 0.1049 |  | 1259.6 | | -0.0277 | -0.0411 |
| 655.7 | -0.1745 | -0.1091 |  | 781.2 | -0.0061 | -0.0271 |  | 1283.5 | | 0.0023 | -0.0021 |
| 650.8 | 0.0455 | 0.0709 |  | 805.1 | 0.0139 | -0.0481 |  | 1274.9 | | -0.0477 | -0.0871 |
| 692 | 0.0755 | 0.0589 |  | 829 | 0.0139 | -0.0521 |  | 1322.8 | | 0.0323 | 0.0189 |
| 674.8 | 0.0355 | 0.1069 |  | 820.3 | -0.0061 | 0.0379 |  | 1346.7 | | 0.0423 | -0.0161 |
| 715.9 | 0.0955 | 0.0589 |  | 853 | 0.0139 | 0.0039 |  | 1394.6 | | 0.0423 | 0.0529 |
| 741.9 | 0.0455 | 0.0679 |  | 861.6 | 0.0739 | 0.0599 |  | 1418.5 | | 0.0423 | 0.0409 |
| 724.6 | 0.1555 | 0.1479 |  | 885.5 | 0.1039 | 0.0619 |  | 1485.8 | | 0.1223 | 0.1209 |
| 765.8 | 0.0255 | 0.0439 |  | 900.8 | 0.0139 | -0.0061 |  | 1564.1 | | -0.1977 | -0.1971 |
| 781.2 | 0.0155 | -0.0271 |  | 892.1 | -0.0261 | 0.0089 |  | 1644.6 | | -0.0177 | -0.0781 |
| 772.5 | 0.1755 | 0.1239 |  | 924.8 | 0.0039 | 0.0369 |  | 1635.9 | | -0.1977 | -0.1481 |
| 796.4 | 0.1555 | 0.1299 |  | 916.1 | -0.0061 | 0.0099 |  | 1668.5 | | 0.0023 | -0.0371 |
| 829 | 0.0155 | -0.0521 |  | 948.7 | 0.0239 | 0.0279 |  | 1692.5 | | -0.0077 | 0.0079 |
| 861.6 | 0.1255 | 0.0599 |  | 940 | -0.0161 | 0.0209 |  | 1716.4 | | -0.0077 | -0.0651 |
| 844.2 | 0.1455 | 0.0899 |  | 981.3 | 0.1139 | 0.1939 |  | 1875.2 | | -0.0277 | -0.0491 |
| 867 | 0.0755 | 0.0689 |  | 996.6 | 0.0139 | 0.0859 |  | 1899.2 | | -0.1277 | -0.0811 |
| 876.9 | 0.0455 | 0.1009 |  | 1020.5 | 0.0139 | -0.0521 |  | 1931.8 | | -0.0977 | -0.0861 |
| 885.5 | 0.1155 | 0.0619 |  | 1011.8 | -0.0161 | 0.0529 |  | 1923.1 | | 0.0123 | 0.0189 |
| 900.8 | 0.0555 | -0.0061 |  | 1059.7 | 0.0039 | -0.0021 |  | 1955.7 | | -0.0277 | -0.0216 |
| 924.8 | 0.0655 | 0.0369 |  | 1083.6 | 0.0039 | 0.0229 |  | 1947 | | -0.0077 | -0.0231 |
| 954.3 | -0.0345 | -0.0061 |  | 1107.5 | 0.0039 | 0.0709 |  | 1979.6 | | -0.0277 | 0.0174 |
| 948.7 | 0.0855 | 0.0279 |  | 1131.5 | -0.0061 | 0.0619 |  | 1971 | | 0.0023 | -0.0146 |
| 972.6 | 0.1055 | 0.1729 |  | 1164.1 | 0.0139 | -0.0411 |  | 2003.5 | | 0.0023 | 0.0479 |
| 996.6 | 0.0555 | 0.0859 |  | 1184.3 | -0.0261 | -0.0051 |  | 1994.9 | | -0.0477 | 0.0044 |
| 987.9 | 0.1655 | 0.0949 |  | 1179.3 | 0.0039 | 0.0469 |  | 2018.9 | | 0.0123 | 0.0664 |
| 1035.7 | 0.1755 | 0.1319 |  | 1203.3 | 0.0039 | -0.0681 |  | 2060.2 | | 0.1123 | 0.0809 |
| 1172.7 | 0.0755 | 0.0799 |  | 1265.6 | -0.1761 | -0.1581 |  | 2042.8 | | 0.0323 | 0.0749 |
| 1179.3 | 0.0855 | 0.0469 |  | 1259.8 | 0.0039 | -0.0371 |  | 2075.3 | | -0.0177 | -0.0661 |
| 1232.1 | -0.1345 | -0.1561 |  | 1283.7 | 0.0239 | 0.0009 |  | 2084.1 | | 0.1023 | 0.0459 |
| 1256.1 | -0.1945 | -0.1391 |  | 1307.7 | 0.0139 | 0.0419 |  | 2114.6 | | 0.0423 | -0.0041 |
| 1280 | -0.1745 | -0.1651 |  | 1299 | 0.0139 | 0.0589 |  | 2138.5 | | 0.0423 | -0.0001 |
| 1292.3 | 0.0655 | 0.0409 |  | 1323 | 0.0239 | -0.0171 |  |  | |  |  |
| 1307.7 | 0.0455 | 0.0419 |  | 1346.9 | 0.0139 | -0.0351 |  |  | |  |  |
| 1299 | 0.0355 | 0.0589 |  | 1394.8 | 0.0039 | 0.0569 |  |  | |  |  |
| 1323 | 0.0455 | -0.0171 |  | 1418.7 | 0.0139 | 0.0569 |  |  | |  |  |
| 1364.2 | -0.0145 | 0.0489 |  | 1443.4 | 0.0939 | 0.0589 |  |  | |  |  |
| 1346.9 | 0.0255 | -0.0351 |  | 1444.6 | -0.0061 | 0.0369 |  |  | |  |  |
| 1375.7 | -0.1745 | -0.1581 |  | 1467.3 | 0.1139 | 0.0909 |  |  | |  |  |
| 1388.1 | 0.0355 | -0.0111 |  | 1477.2 | 0.0239 | -0.0451 |  |  | |  |  |
| 1412 | 0.0755 | 0.0059 |  | 1491.2 | 0.1739 | 0.0879 |  |  | |  |  |
| 1394.8 | 0.0355 | 0.0569 |  | 1516.4 | -0.0161 | -0.0101 |  |  | |  |  |
| 1418.7 | 0.0455 | 0.0569 |  | 1540.3 | -0.0061 | -0.0661 |  |  | |  |  |
| 1461.9 | 0.0455 | 0.0549 |  | 1612.1 | -0.0261 | -0.0931 |  |  | |  |  |
| 1485.8 | 0.0255 | 0.0729 |  | 1644.8 | 0.0039 | -0.0751 |  |  | |  |  |
| 1509.8 | 0.0155 | -0.0141 |  | 1668.7 | 0.0239 | -0.0461 |  |  | |  |  |
| 1492.5 | 0.1755 | 0.1519 |  | 1692.6 | 0.0339 | -0.0111 |  |  | |  |  |
| 1539.1 | -0.1345 | -0.0921 |  | 1683.9 | -0.0161 | -0.0401 |  |  | |  |  |
| 1610.9 | -0.1245 | -0.1121 |  | 1716.6 | 0.0139 | -0.0091 |  |  | |  |  |
| 1634.9 | -0.1345 | -0.0991 |  | 1707.9 | 0.0039 | 0.0799 |  |  | |  |  |
| 1674.3 | -0.0345 | -0.0611 |  | 1731.8 | -0.0161 | -0.0121 |  |  | |  |  |
| 1716.6 | 0.0555 | -0.0091 |  | 1755.7 | 0.0039 | 0.0199 |  |  | |  |  |
| 1868.8 | 0.0555 | 0.0369 |  | 1779.7 | 0.0039 | 0.0889 |  |  | |  |  |
| 1851.5 | 0.1455 | 0.1279 |  | 1803.6 | 0.0039 | -0.0341 |  |  | |  |  |
| 1916.6 | 0.0655 | 0.0044 |  | 1827.5 | 0.0039 | 0.0429 |  |  | |  |  |
| 1940.6 | 0.0055 | -0.0451 |  | 1875.4 | 0.0039 | -0.0261 |  |  | |  |  |
| 1923.3 | 0.0755 | 0.0279 |  | 1904.3 | -0.0261 | 0.0459 |  |  | |  |  |
| 1964.5 | 0.0055 | -0.0666 |  | 1923.3 | 0.0039 | 0.0279 |  |  | |  |  |
| 1947.2 | 0.0355 | -0.0071 |  | 1952.1 | 0.0239 | -0.0006 |  |  | |  |  |
| 1979.8 | 0.0655 | 0.0019 |  | 1955.9 | 0.0139 | -0.0376 |  |  | |  |  |
| 1971.2 | 0.0355 | -0.0141 |  | 1947.2 | 0.0139 | -0.0071 |  |  | |  |  |
| 2003.7 | 0.1155 | 0.0664 |  | 1985.6 | -0.1761 | -0.1056 |  |  | |  |  |
| 2027.7 | 0.0455 | 0.0719 |  | 1979.8 | 0.0039 | 0.0019 |  |  | |  |  |
| 2019 | 0.0355 | 0.0779 |  | 1971.2 | 0.0039 | -0.0141 |  |  | |  |  |
| 2043 | 0.0455 | 0.0249 |  | 2003.7 | 0.0239 | 0.0664 |  |  | |  |  |
| 2108.1 | 0.0355 | -0.0231 |  | 1995.1 | -0.0061 | -0.0001 |  |  | |  |  |
| 2114.8 | 0.0355 | 0.0109 |  | 2027.7 | 0.0139 | 0.0719 |  |  | |  |  |
| 2138.7 | 0.0455 | 0.0169 |  | 2019 | 0.0139 | 0.0779 |  |  | |  |  |
|  |  |  |  | 2043 | 0.0239 | 0.0249 |  |  | |  |  |
|  |  |  |  | 2075.6 | 0.0339 | -0.0491 |  |  | |  |  |
|  |  |  |  | 2114.8 | 0.0039 | 0.0109 |  |  | |  |  |
|  |  |  |  | 2138.7 | 0.0139 | 0.0169 |  |  | |  |  |

| Table S2: We studied GPS (L1, L2 and L5) derived tide gauge (TG) measurements for the current study. In this table column 1 indicates time in hours from 01 October 2019 to 31 December 2019. Column 2, the GPS (L1) signal derived results which is subtracted from its mean. Column 3 indicates corresponding GSI-TG values, from which its mean value is subtracted. Similarly, column 4 time in hours, column 5 is GPS (L2) signal derived results and column 6 is GSI-TG. In the same way, column 7 is again time in hours, column 8 is QZSS (L5) signal derived results and column 6 is GSI-TG. | | | | | | | | | | |
| --- | --- | --- | --- | --- | --- | --- | --- | --- | --- | --- |
| Time in Hours from 01 October 2019 | GPS-TG  (L1)  (Mean-Derived) | GSI-TG  (Derived -Mean) |  | Time in Hours from 01 October 2019 | GPS-TG  (L2)  (Mean-Derived) | GSI-TG  (Derived -Mean) |  | Time in Hours from 01 October 2019 | GPS-TG  (L5)  (Mean-Derived) | GSI-TG  (Derived -Mean) |
| 20.8 | 0.22 | 0.1479 |  | 22.4 | 0.0216 | 0.1089 |  | 20.8 | 0.1899 | 0.1639 |
| 9.9 | 0.06 | -0.0041 |  | 1.2 | 0.2316 | 0.1719 |  | 16.7 | 0.1899 | 0.2589 |
| 10.9 | -0.01 | -0.0121 |  | 9.9 | 0.0516 | -0.0041 |  | 13.1 | 0.0499 | 0.0939 |
| 4.5 | 0.17 | 0.2429 |  | 16.7 | 0.2116 | 0.2569 |  | 10.7 | 0.0299 | 0.0059 |
| 7.6 | 0.08 | 0.0889 |  | 4.5 | 0.2016 | 0.2429 |  | 44.7 | 0.1999 | 0.1789 |
| 20.9 | 0.09 | 0.1219 |  | 4.5 | 0.3216 | 0.2429 |  | 40.6 | 0.1999 | 0.2229 |
| 15.8 | 0.28 | 0.2339 |  | 7.6 | 0.1116 | 0.0889 |  | 34.8 | 0.1099 | -0.0071 |
| 9.2 | 0.08 | 0.0259 |  | 4.6 | 0.2716 | 0.2149 |  | 37 | 0.0499 | 0.0449 |
| 9.2 | 0.08 | 0.0259 |  | 9.2 | 0.0816 | 0.0259 |  | 34.6 | 0.0099 | 0.0159 |
| 44.8 | 0.23 | 0.1729 |  | 41 | 0.1216 | 0.2209 |  | 58.7 | 0.1199 | 0.1719 |
| 42.1 | 0.29 | 0.2199 |  | 45.8 | 0.2516 | 0.1559 |  | 61 | 0.0399 | 0.1499 |
| 45.8 | 0.2 | 0.1559 |  | 25.7 | 0.1716 | 0.1819 |  | 92.6 | 0.1899 | 0.2599 |
| 30.4 | 0.25 | 0.1749 |  | 33.8 | 0.0916 | 0.0239 |  | 116.5 | 0.1999 | 0.3199 |
| 25.7 | 0.15 | 0.1819 |  | 38 | 0.0216 | 0.0809 |  | 112.4 | 0.1999 | 0.1659 |
| 33.8 | 0.1 | 0.0239 |  | 28.5 | 0.1616 | 0.2149 |  | 106.6 | 0.1199 | 0.2109 |
| 34.8 | -0.02 | -0.0071 |  | 33.1 | 0.0116 | 0.0489 |  | 140.4 | 0.1699 | 0.0699 |
| 25.4 | 0.08 | 0.1599 |  | 58.1 | 0.1416 | 0.1679 |  | 136.6 | 0.0099 | -0.0181 |
| 38 | 0.05 | 0.0809 |  | 68.7 | 0.2916 | 0.3839 |  | 130.5 | 0.1199 | 0.1229 |
| 38 | 0.03 | 0.0809 |  | 57 | 0.3016 | 0.2219 |  | 124.2 | 0.1499 | 0.2059 |
| 28.5 | 0.13 | 0.2129 |  | 64.9 | 0.4216 | 0.3349 |  | 132.8 | 0.0699 | 0.0789 |
| 28.5 | 0.15 | 0.2149 |  | 57.8 | 0.1016 | 0.1989 |  | 130.4 | 0.0299 | 0.1229 |
| 47 | 0.23 | 0.1579 |  | 57.9 | 0.1816 | 0.1939 |  | 139.6 | 0.0799 | 0.0319 |
| 47 | 0.22 | 0.1579 |  | 52.4 | 0.2316 | 0.3289 |  | 146.9 | 0.0599 | 0.0969 |
| 30.7 | 0.12 | 0.1689 |  | 52.4 | 0.3116 | 0.3289 |  | 154.5 | 0.1199 | 0.0359 |
| 30.7 | 0.24 | 0.1689 |  | 61 | 0.1316 | 0.1499 |  | 148.1 | 0.1699 | 0.0799 |
| 44.9 | 0.09 | 0.1649 |  | 55.4 | 0.2616 | 0.2739 |  | 156.7 | 0.0599 | 0.0379 |
| 39.7 | 0.27 | 0.1909 |  | 92.6 | 0.2816 | 0.2679 |  | 154.3 | 0.0199 | 0.0849 |
| 33.1 | 0.05 | 0.0489 |  | 80.9 | 0.3116 | 0.3649 |  | 178.4 | 0.1199 | 0.1629 |
| 58.1 | 0.12 | 0.1679 |  | 88.8 | 0.4216 | 0.3269 |  | 172.1 | 0.1399 | 0.1229 |
| 57 | 0.25 | 0.2219 |  | 93.6 | 0.2516 | 0.2949 |  | 180.6 | 0.0499 | 0.1579 |
| 57.9 | 0.17 | 0.1939 |  | 94.8 | 0.2616 | 0.2999 |  | 187.4 | 0.0599 | 0.0479 |
| 55.4 | 0.22 | 0.2759 |  | 116.6 | 0.2716 | 0.3179 |  | 194.7 | 0.0799 | 0.0529 |
| 56.7 | 0.17 | 0.2369 |  | 104.8 | 0.2916 | 0.3329 |  | 202.3 | 0.1199 | 0.0859 |
| 92.6 | 0.27 | 0.2679 |  | 112.8 | 0.1316 | 0.1809 |  | 196 | 0.1699 | 0.0509 |
| 92.6 | 0.23 | 0.2679 |  | 117.6 | 0.3116 | 0.3219 |  | 204.6 | 0.0599 | 0.0659 |
| 93.6 | 0.22 | 0.2949 |  | 102.2 | 0.4216 | 0.3519 |  | 225.7 | -0.0101 | 0.0239 |
| 81.8 | 0.28 | 0.3239 |  | 109.8 | 0.0716 | 0.1469 |  | 228.5 | 0.0899 | 0.0509 |
| 94.8 | 0.22 | 0.2999 |  | 105.7 | 0.2016 | 0.2729 |  | 226.3 | 0.1299 | 0.0389 |
| 94.8 | 0.22 | 0.2999 |  | 100.3 | 0.3216 | 0.3669 |  | 228.5 | 0.0499 | 0.0349 |
| 87.6 | 0.27 | 0.2729 |  | 118.8 | 0.2916 | 0.2729 |  | 226.1 | 0.0299 | 0.0599 |
| 116.6 | 0.25 | 0.3179 |  | 135.1 | -0.0684 | 0.0019 |  | 242.6 | 0.0699 | 0.0959 |
| 104.8 | 0.25 | 0.3329 |  | 135.9 | -0.0784 | -0.0261 |  | 250.2 | 0.1299 | 0.0899 |
| 113.9 | 0.29 | 0.2109 |  | 133.7 | 0.0616 | 0.0339 |  | 252.4 | 0.0499 | 0.1319 |
| 109.8 | 0.08 | 0.1469 |  | 129.7 | 0.2016 | 0.1449 |  | 250 | 0.0099 | 0.1119 |
| 109.8 | 0.08 | 0.1469 |  | 124.2 | 0.2016 | 0.2059 |  | 259.2 | 0.0999 | 0.0429 |
| 105.7 | 0.27 | 0.2729 |  | 132.8 | 0.1216 | 0.0789 |  | 273.5 | -0.0001 | 0.0179 |
| 105.7 | 0.31 | 0.2729 |  | 140.6 | -0.0484 | 0.0349 |  | 284 | 0.1899 | 0.0749 |
| 118.8 | 0.23 | 0.2729 |  | 140.6 | 0.0516 | 0.0349 |  | 266.5 | 0.0999 | 0.1099 |
| 118.8 | 0.24 | 0.2729 |  | 127.2 | 0.2616 | 0.1939 |  | 280 | 0.2099 | 0.1369 |
| 108.3 | 0.25 | 0.2039 |  | 162.4 | -0.1284 | -0.0551 |  | 274.1 | 0.1199 | 0.0749 |
| 118.6 | 0.24 | 0.3139 |  | 150.1 | 0.0516 | 0.0799 |  | 276.3 | 0.0599 | 0.1339 |
| 128.8 | 0.25 | 0.1689 |  | 153.5 | 0.0916 | 0.0539 |  | 273.9 | 0.0099 | 0.0479 |
| 135.9 | -0.11 | -0.0261 |  | 159.1 | 0.0216 | -0.0241 |  | 297.4 | -0.0101 | -0.0241 |
| 135.9 | -0.04 | -0.0261 |  | 159.8 | -0.1184 | -0.0591 |  | 290.5 | 0.0699 | 0.1439 |
| 133.7 | 0.08 | 0.0339 |  | 157.6 | 0.0916 | 0.0119 |  | 298.1 | 0.1099 | 0.0009 |
| 133.7 | 0.06 | 0.0339 |  | 164.6 | 0.0416 | 0.0309 |  | 291.7 | 0.0199 | 0.1159 |
| 129.7 | 0.17 | 0.1449 |  | 146.3 | 0.0216 | 0.0619 |  | 300.3 | -0.0301 | 0.0439 |
| 124.2 | 0.14 | 0.2059 |  | 147.8 | 0.0716 | 0.0579 |  | 297.9 | -0.0001 | 0.0429 |
| 124.2 | 0.17 | 0.2059 |  | 163.5 | -0.0984 | -0.0441 |  | 328.1 | 0.0099 | 0.0819 |
| 134.1 | 0.07 | 0.0229 |  | 189.9 | -0.0184 | 0.0549 |  | 327.8 | 0.2099 | 0.0939 |
| 140.6 | 0.06 | 0.0349 |  | 184.6 | 0.0616 | 0.0419 |  | 324.2 | 0.0399 | 0.0049 |
| 140.6 | -0.03 | 0.0349 |  | 169.3 | 0.1416 | 0.1059 |  | 321.8 | 0.0099 | -0.0801 |
| 127.2 | 0.21 | 0.1939 |  | 183 | 0.1616 | 0.0799 |  | 338.3 | 0.0799 | 0.0869 |
| 128.5 | 0.15 | 0.1879 |  | 181.6 | 0.0916 | 0.1109 |  | 401.7 | -0.0501 | 0.0609 |
| 128.5 | 0.23 | 0.1879 |  | 177.6 | 0.1716 | 0.1599 |  | 387.4 | 0.0499 | 0.0779 |
| 146.3 | 0.07 | 0.0539 |  | 172.1 | 0.1816 | 0.1229 |  | 396 | -0.0001 | -0.1041 |
| 145.4 | 0.11 | 0.1199 |  | 185.8 | -0.0084 | 0.0089 |  | 410.1 | 0.0799 | 0.0579 |
| 154.5 | 0.01 | 0.0359 |  | 170.3 | 0.0416 | 0.1339 |  | 411.4 | 0.0499 | 0.0629 |
| 159.8 | -0.09 | -0.0591 |  | 171.8 | 0.0716 | 0.1179 |  | 419.9 | 0.0099 | -0.0791 |
| 157.6 | 0.05 | 0.0119 |  | 210.3 | -0.1284 | -0.0751 |  | 417.6 | -0.0101 | -0.0551 |
| 157.6 | 0.08 | 0.0119 |  | 213.9 | -0.0084 | -0.0401 |  | 426.8 | 0.0699 | 0.0459 |
| 164.6 | 0.07 | 0.0309 |  | 192.7 | 0.1216 | 0.0939 |  | 451.5 | 0.1099 | 0.0959 |
| 146.3 | 0.04 | 0.0619 |  | 193.3 | 0.1516 | 0.0719 |  | 434.1 | 0.0599 | 0.1429 |
| 159.3 | -0.14 | -0.0581 |  | 205.5 | 0.0816 | 0.0389 |  | 435.3 | 0.0599 | 0.1559 |
| 163.5 | -0.07 | -0.0441 |  | 212.4 | -0.0684 | -0.0511 |  | 443.9 | 0.0499 | -0.0191 |
| 152.8 | 0.08 | 0.0349 |  | 195.7 | 0.0716 | 0.0599 |  | 441.5 | 0.0099 | 0.0219 |
| 152.5 | 0.12 | 0.0649 |  | 211.4 | -0.1184 | -0.0881 |  | 475.5 | 0.1599 | 0.0639 |
| 171.4 | 0.08 | 0.1189 |  | 222.6 | -0.0384 | 0.0199 |  | 471.7 | 0.0099 | -0.0121 |
| 189.9 | 0 | 0.0549 |  | 234.2 | -0.0884 | -0.0801 |  | 458 | 0.0999 | 0.1549 |
| 189.9 | 0 | 0.0549 |  | 237.8 | -0.0084 | -0.0291 |  | 465.6 | 0.1199 | 0.0479 |
| 176.6 | 0.24 | 0.1789 |  | 225.3 | 0.0816 | 0.0579 |  | 459.2 | 0.0999 | 0.1459 |
| 169.3 | 0.06 | 0.1059 |  | 230.9 | -0.0084 | 0.0079 |  | 467.8 | 0.0499 | -0.0311 |
| 169.3 | 0.14 | 0.1059 |  | 231.6 | -0.1184 | -0.0381 |  | 465.4 | 0.0099 | 0.0399 |
| 183 | 0.12 | 0.0799 |  | 229.4 | 0.0716 | 0.0289 |  | 495.6 | 0.0199 | -0.0831 |
| 181.6 | 0.06 | 0.1109 |  | 218.1 | 0.0616 | 0.0389 |  | 481.9 | 0.0999 | 0.1099 |
| 181.6 | 0.07 | 0.1109 |  | 219.7 | 0.0616 | 0.0549 |  | 489.5 | 0.1199 | 0.0049 |
| 172.1 | 0.1 | 0.1229 |  | 235.3 | -0.0884 | -0.0941 |  | 483.1 | 0.0999 | 0.1229 |
| 172.1 | 0.16 | 0.1229 |  | 246.6 | -0.0384 | 0.0519 |  | 491.7 | 0.0499 | -0.0291 |
| 188.5 | 0.08 | 0.0539 |  | 261.7 | -0.0084 | 0.0189 |  | 489.3 | 0.0099 | 0.0499 |
| 170.3 | 0.05 | 0.1339 |  | 256.4 | 0.1216 | 0.0919 |  | 519.5 | 0.0499 | -0.0431 |
| 175 | 0.2 | 0.1289 |  | 249.2 | 0.1016 | 0.0659 |  | 505.9 | 0.0599 | 0.1249 |
| 210.3 | -0.14 | -0.0751 |  | 253.4 | 0.0816 | 0.1279 |  | 515.7 | 0.0999 | 0.0429 |
| 213.9 | 0.01 | -0.0401 |  | 252.4 | 0.1216 | 0.1319 |  | 513.5 | 0.1299 | 0.1369 |
| 213.9 | 0 | -0.0401 |  | 242.1 | 0.0316 | 0.0879 |  | 507.1 | 0.1099 | 0.1169 |
| 193.3 | 0.07 | 0.0719 |  | 243.6 | 0.0316 | 0.0879 |  | 515.7 | 0.0599 | 0.0629 |
| 193.3 | 0.13 | 0.0719 |  | 270.5 | -0.0484 | 0.0369 |  | 513.3 | 0.0199 | 0.1339 |
| 201.4 | 0.07 | 0.0899 |  | 285.7 | 0.0116 | 0.0719 |  | 545.3 | -0.0101 | -0.0401 |
| 201.4 | 0.09 | 0.0899 |  | 280.3 | 0.1216 | 0.1299 |  | 529.8 | 0.0699 | 0.1339 |
| 207.7 | -0.08 | -0.0081 |  | 273.2 | 0.1116 | 0.0199 |  | 537.4 | 0.1199 | 0.1609 |
| 205.5 | 0.05 | 0.0389 |  | 277.3 | 0.0516 | 0.1509 |  | 531 | 0.1099 | 0.1179 |
| 205.5 | 0.07 | 0.0389 |  | 267.8 | 0.1716 | 0.1299 |  | 539.6 | 0.0699 | 0.0929 |
| 211.7 | -0.06 | -0.0921 |  | 266 | 0.0616 | 0.1419 |  | 553.7 | 0.0799 | 0.1319 |
| 212.4 | 0.03 | -0.0511 |  | 267.5 | 0.0616 | 0.1419 |  | 563.6 | 0.0899 | 0.0919 |
| 212.4 | -0.04 | -0.0511 |  | 309.6 | 0.0516 | -0.0451 |  | 561.3 | 0.1199 | 0.1039 |
| 194.2 | 0.03 | 0.0709 |  | 294.4 | -0.0184 | 0.0529 |  | 555 | 0.1099 | 0.1049 |
| 211.4 | -0.08 | -0.0881 |  | 306 | -0.0984 | -0.0011 |  | 563.5 | 0.0599 | 0.1079 |
| 200.7 | 0.06 | 0.0709 |  | 309.6 | 0.0316 | -0.0611 |  | 561.1 | 0.0099 | 0.1109 |
| 222.6 | -0.02 | 0.0199 |  | 304.2 | 0.1116 | 0.0379 |  | 570.4 | -0.2201 | -0.1121 |
| 234.2 | -0.15 | -0.0801 |  | 302.7 | 0.0016 | 0.0689 |  | 591.3 | -0.0001 | 0.0289 |
| 237.8 | 0.02 | -0.0291 |  | 301.2 | 0.0616 | 0.0609 |  | 587.5 | 0.0599 | 0.0989 |
| 237.8 | 0.01 | -0.0291 |  | 291.7 | 0.2016 | 0.1159 |  | 585.2 | 0.1199 | 0.0709 |
| 216.6 | 0.08 | 0.0289 |  | 308.2 | -0.0484 | -0.0881 |  | 578.9 | 0.0999 | 0.0399 |
| 216.6 | 0.08 | 0.0289 |  | 291.4 | 0.0616 | 0.1459 |  | 587.5 | 0.0599 | 0.0849 |
| 217.2 | 0.09 | 0.0479 |  | 307.1 | -0.0984 | -0.0611 |  | 585.1 | 0.0099 | 0.0469 |
| 225.3 | 0.07 | 0.0579 |  | 318.4 | -0.0284 | -0.0531 |  | 611.4 | 0.0599 | 0.1339 |
| 225.3 | 0.07 | 0.0579 |  | 322 | -0.0884 | -0.0631 |  | 615 | 0.2099 | 0.1019 |
| 226.3 | -0.02 | 0.0389 |  | 333.5 | -0.0184 | -0.0151 |  | 609.2 | 0.1099 | 0.0399 |
| 231.6 | -0.08 | -0.0381 |  | 328.1 | 0.1116 | 0.0709 |  | 602.8 | 0.1099 | 0.0439 |
| 229.4 | 0.03 | 0.0289 |  | 326.6 | 0.0316 | 0.0739 |  | 605.9 | -0.0401 | -0.0311 |
| 229.4 | 0.09 | 0.0289 |  | 327.4 | -0.0284 | 0.0549 |  | 611.4 | 0.0699 | 0.1219 |
| 219.9 | 0.1 | 0.0249 |  | 325.2 | 0.0416 | 0.0479 |  | 609 | 0.0099 | 0.0329 |
| 218.1 | 0.06 | 0.0389 |  | 332.1 | -0.0184 | -0.0201 |  | 635.4 | 0.0399 | 0.0949 |
| 235.3 | -0.05 | -0.0941 |  | 313.9 | 0.0416 | 0.0079 |  | 638.9 | 0.2099 | 0.1129 |
| 224.6 | 0.07 | 0.0539 |  | 315.4 | 0.0716 | 0.0139 |  | 626.8 | 0.0899 | 0.1269 |
| 261.8 | -0.02 | 0.0269 |  | 331.1 | -0.0884 | -0.0041 |  | 635.3 | 0.0399 | 0.0729 |
| 246.6 | -0.02 | 0.0519 |  | 342.3 | -0.0184 | -0.0581 |  | 632.9 | 0.0099 | -0.0281 |
| 246.7 | 0.02 | 0.0519 |  | 353.9 | -0.0884 | -0.0601 |  | 663.1 | -0.0001 | 0.0859 |
| 261.7 | 0 | 0.0189 |  | 357.5 | -0.0384 | -0.1331 |  | 649.5 | 0.1799 | 0.0979 |
| 261.7 | -0.01 | 0.0189 |  | 344.1 | -0.2084 | -0.1271 |  | 662.9 | 0.1999 | 0.0999 |
| 240.5 | 0.03 | 0.0719 |  | 336.9 | 0.1316 | 0.0529 |  | 650.7 | 0.0999 | 0.0639 |
| 241.1 | 0.09 | 0.0899 |  | 350.6 | 0.0316 | 0.0189 |  | 659.3 | 0.0299 | -0.0201 |
| 241.1 | 0.15 | 0.0899 |  | 356 | -0.0384 | -0.0991 |  | 656.9 | -0.0301 | -0.1171 |
| 249.2 | 0.03 | 0.0659 |  | 356 | -0.0184 | -0.0991 |  | 690.8 | 0.1599 | 0.1179 |
| 249.2 | 0.1 | 0.0659 |  | 345.8 | -0.2084 | -0.1271 |  | 673.4 | 0.1799 | 0.0729 |
| 254.8 | 0.13 | 0.1079 |  | 339.3 | 0.0716 | 0.0739 |  | 686.8 | 0.2099 | 0.1189 |
| 253.4 | 0.06 | 0.1279 |  | 355 | -0.0884 | -0.0841 |  | 674.6 | 0.0999 | 0.0799 |
| 253.4 | 0.09 | 0.1279 |  | 377.8 | -0.0784 | 0.0049 |  | 677.7 | -0.1001 | -0.0021 |
| 249.3 | 0.16 | 0.0779 |  | 381.4 | -0.0084 | -0.0491 |  | 714.8 | 0.1699 | 0.1149 |
| 260.3 | 0.07 | 0.0179 |  | 368 | -0.2084 | -0.1971 |  | 711 | 0.0299 | 0.0739 |
| 242.1 | 0.03 | 0.0879 |  | 363.7 | -0.0284 | -0.0681 |  | 697.3 | 0.1899 | 0.1489 |
| 248.5 | 0.07 | 0.0669 |  | 375.2 | -0.1084 | -0.0571 |  | 710.7 | 0.1999 | 0.0869 |
| 270.5 | -0.02 | 0.0369 |  | 380 | -0.0584 | -0.0471 |  | 698.5 | 0.0899 | 0.2039 |
| 271.8 | 0.04 | 0.0089 |  | 380 | -0.0284 | -0.0471 |  | 707.1 | -0.0001 | -0.1001 |
| 285.7 | 0.02 | 0.0719 |  | 369.7 | -0.1984 | -0.2201 |  | 704.7 | -0.0301 | -0.0751 |
| 285.7 | 0.02 | 0.0719 |  | 378.9 | -0.0884 | -0.0271 |  | 740.8 | 0.1899 | 0.0859 |
| 265.1 | 0.1 | 0.1359 |  | 366.7 | -0.1884 | -0.1541 |  | 723.3 | 0.0999 | 0.1419 |
| 265.1 | 0.13 | 0.1359 |  | 390.1 | -0.0684 | 0.0089 |  | 724.5 | 0.1199 | 0.1429 |
| 274.1 | 0.01 | 0.0749 |  | 405.3 | 0.0116 | -0.0061 |  | 730.7 | 0.0299 | -0.0691 |
| 277.3 | 0.07 | 0.1509 |  | 392 | -0.1784 | -0.0851 |  | 739.9 | 0.0499 | 0.0739 |
| 267.8 | 0.12 | 0.1299 |  | 399.9 | 0.1316 | 0.0369 |  | 747.2 | 0.0799 | 0.1179 |
| 267.8 | 0.14 | 0.1299 |  | 398.4 | 0.0216 | -0.0171 |  | 763.8 | 0.0699 | 0.0479 |
| 284.2 | 0.06 | 0.1149 |  | 387.4 | 0.1516 | 0.0779 |  | 788.6 | 0.1899 | 0.1509 |
| 266 | 0.06 | 0.1419 |  | 403.9 | -0.0484 | 0.0159 |  | 786.6 | 0.0999 | 0.0679 |
| 294.4 | 0 | 0.0529 |  | 403.9 | -0.0184 | 0.0159 |  | 771.1 | 0.0799 | 0.1129 |
| 302.7 | 0.08 | 0.0689 |  | 387.2 | 0.0616 | 0.0439 |  | 778.8 | 0.1199 | 0.0149 |
| 298.1 | 0.01 | -0.0131 |  | 402.9 | -0.0684 | 0.0209 |  | 772.4 | 0.1299 | 0.1399 |
| 301.2 | 0.07 | 0.0609 |  | 429.3 | 0.0616 | 0.0349 |  | 781 | 0.0399 | -0.0411 |
| 301.2 | 0.07 | 0.0609 |  | 414.1 | -0.0284 | 0.0649 |  | 787.8 | 0.0699 | 0.1329 |
| 291.7 | 0.17 | 0.1159 |  | 422.4 | -0.0984 | -0.0611 |  | 802.7 | 0.1199 | 0.0059 |
| 308.2 | -0.05 | -0.0881 |  | 429.3 | -0.0084 | 0.0379 |  | 804.9 | 0.0399 | -0.0561 |
| 307.1 | -0.06 | -0.0761 |  | 423.9 | 0.1016 | 0.0479 |  | 802.5 | 0.0099 | 0.0239 |
| 296.4 | 0.09 | 0.0349 |  | 422.4 | 0.0416 | -0.0581 |  | 811.7 | 0.0999 | 0.0009 |
| 318.4 | -0.01 | -0.0531 |  | 411.2 | -0.0084 | 0.0689 |  | 819 | 0.0899 | 0.0429 |
| 322 | -0.03 | -0.0631 |  | 418.6 | -0.0784 | -0.1091 |  | 826.6 | 0.1199 | 0.0139 |
| 322 | -0.05 | -0.0631 |  | 411.1 | 0.0716 | 0.0609 |  | 820.3 | 0.1399 | 0.0419 |
| 333.5 | 0.03 | -0.0151 |  | 447.8 | 0.1316 | 0.0329 |  | 828.8 | 0.0399 | -0.0491 |
| 333.5 | -0.01 | -0.0151 |  | 446.3 | 0.0116 | -0.0591 |  | 826.4 | 0.0099 | 0.0139 |
| 322 | -0.03 | -0.0641 |  | 435.3 | 0.1716 | 0.1559 |  | 856.6 | 0.0099 | -0.0431 |
| 327.4 | 0 | 0.0549 |  | 435.3 | 0.1916 | 0.1559 |  | 850.5 | 0.1199 | 0.0749 |
| 325.2 | 0.05 | 0.0479 |  | 442.6 | -0.0484 | -0.0391 |  | 844.2 | 0.1499 | 0.0919 |
| 325.2 | 0.04 | 0.0479 |  | 435 | 0.0816 | 0.1019 |  | 852.8 | 0.0699 | 0.0049 |
| 315.6 | 0.11 | 0.0359 |  | 471 | -0.0684 | -0.0201 |  | 850.4 | 0.0299 | 0.0859 |
| 332.1 | 0.04 | -0.0201 |  | 463.9 | 0.0516 | 0.1029 |  | 859.6 | 0.0799 | -0.0291 |
| 332.1 | -0.02 | -0.0201 |  | 464.7 | 0.1716 | 0.0719 |  | 866.9 | 0.0599 | 0.0969 |
| 313.9 | 0.06 | 0.0079 |  | 459.2 | 0.1916 | 0.1459 |  | 874.5 | 0.1199 | 0.1299 |
| 331.1 | -0.05 | -0.0041 |  | 459.2 | 0.2316 | 0.1459 |  | 868.1 | 0.1699 | 0.0789 |
| 342.3 | 0 | -0.0581 |  | 475.7 | 0.0016 | 0.0639 |  | 876.7 | 0.0599 | 0.1059 |
| 353.9 | -0.14 | -0.0601 |  | 462.2 | 0.2416 | 0.1479 |  | 874.3 | 0.0199 | 0.1069 |
| 353.8 | 0 | -0.0551 |  | 459 | 0.0716 | 0.1439 |  | 883.5 | 0.1099 | 0.0319 |
| 341.6 | 0.04 | -0.0041 |  | 497.5 | -0.0784 | -0.0121 |  | 898.4 | 0.1199 | 0.0039 |
| 336.9 | 0.12 | 0.0529 |  | 480.7 | 0.1416 | 0.1119 |  | 892.1 | 0.1399 | 0.0389 |
| 350.6 | 0.09 | 0.0189 |  | 494.1 | 0.0116 | -0.0711 |  | 895.1 | -0.0601 | -0.0201 |
| 356 | -0.02 | -0.0991 |  | 494.9 | -0.0684 | -0.0731 |  | 900.6 | 0.0499 | 0.0089 |
| 337.8 | 0.06 | 0.0659 |  | 483.1 | 0.2016 | 0.1229 |  | 898.2 | 0.0399 | 0.0069 |
| 345.8 | -0.13 | -0.1271 |  | 499.6 | -0.0084 | 0.0479 |  | 907.4 | 0.0599 | -0.0531 |
| 355 | -0.06 | -0.0841 |  | 482.9 | 0.0716 | 0.1239 |  | 914.7 | 0.0799 | 0.0199 |
| 366.2 | -0.21 | -0.1341 |  | 498.6 | -0.0684 | 0.0099 |  | 922.3 | 0.1199 | 0.0239 |
| 381.4 | 0.01 | -0.0491 |  | 525 | 0.0516 | 0.0829 |  | 924.5 | 0.0599 | 0.0519 |
| 381.4 | 0 | -0.0491 |  | 521.4 | -0.1184 | -0.0231 |  | 945.6 | -0.0101 | -0.0351 |
| 363.7 | -0.06 | -0.0681 |  | 525 | 0.0116 | 0.0819 |  | 954.1 | 0.0599 | -0.0321 |
| 375.2 | -0.06 | -0.0571 |  | 524.4 | 0.1716 | 0.0799 |  | 948.5 | 0.0899 | 0.0319 |
| 380 | -0.04 | -0.0471 |  | 506.5 | 0.1616 | 0.1399 |  | 948.5 | 0.0499 | 0.0279 |
| 378.9 | -0.05 | -0.0271 |  | 518.1 | 0.0916 | -0.0031 |  | 946.1 | 0.0299 | -0.0231 |
| 366.7 | -0.11 | -0.1541 |  | 518.1 | 0.0016 | -0.0031 |  | 955.3 | 0.0999 | -0.0021 |
| 406.6 | 0.09 | 0.0109 |  | 516 | 0.0216 | 0.0439 |  | 969.6 | -0.0301 | 0.0429 |
| 390.1 | -0.04 | 0.0089 |  | 516.6 | 0.0916 | 0.0159 |  | 980.1 | 0.1899 | 0.1479 |
| 405.3 | 0.04 | -0.0061 |  | 515.7 | 0.1416 | 0.0629 |  | 962.6 | 0.0699 | 0.1289 |
| 405.3 | 0.01 | -0.0061 |  | 523.5 | 0.0216 | 0.0239 |  | 976 | 0.1999 | 0.1799 |
| 395.8 | -0.09 | -0.1641 |  | 510.1 | 0.2516 | 0.1779 |  | 970.2 | 0.1299 | 0.0409 |
| 401.6 | 0.01 | 0.0719 |  | 506.8 | 0.0616 | 0.1339 |  | 970 | 0.0099 | 0.0539 |
| 384.7 | 0.09 | 0.0219 |  | 522.5 | -0.0684 | -0.0091 |  | 979.2 | 0.0999 | 0.1579 |
| 387.4 | 0.11 | 0.0779 |  | 545.3 | -0.1184 | -0.0431 |  | 993.5 | -0.0001 | -0.0391 |
| 387.4 | 0.13 | 0.0779 |  | 548.9 | 0.0016 | 0.0279 |  | 986.5 | 0.0999 | 0.1659 |
| 396 | -0.11 | -0.1121 |  | 532.5 | 0.2116 | 0.1339 |  | 1000 | 0.2099 | 0.1189 |
| 403.9 | 0 | 0.0159 |  | 542 | -0.0084 | -0.0001 |  | 996.3 | 0.0599 | 0.0529 |
| 393.6 | -0.19 | -0.1141 |  | 542.8 | -0.0684 | -0.0171 |  | 994 | 0.0099 | -0.0261 |
| 402.9 | -0.05 | 0.0209 |  | 540.6 | 0.0416 | 0.0589 |  | 1017.4 | -0.0101 | -0.1271 |
| 414.1 | -0.02 | 0.0649 |  | 539.6 | 0.1116 | 0.0929 |  | 1010.5 | 0.0699 | 0.0689 |
| 422.4 | -0.02 | -0.0611 |  | 529.2 | 0.0416 | 0.1379 |  | 1011.7 | 0.0199 | 0.0549 |
| 422.4 | -0.07 | -0.0611 |  | 530.8 | 0.0616 | 0.1179 |  | 1020.3 | -0.0301 | -0.0441 |
| 429.3 | 0.02 | 0.0379 |  | 546.4 | -0.0784 | -0.0241 |  | 1017.9 | -0.0001 | -0.1121 |
| 429.3 | 0 | 0.0379 |  | 569.3 | -0.1684 | -0.0881 |  | 1048.1 | 0.0099 | 0.0019 |
| 408.6 | 0.07 | 0.0019 |  | 569.3 | -0.1084 | -0.0881 |  | 1041.8 | 0.0099 | -0.0441 |
| 423.1 | -0.09 | -0.0181 |  | 572.9 | 0.0116 | -0.0471 |  | 1058.3 | 0.0799 | 0.0349 |
| 411.2 | 0.01 | 0.0689 |  | 568.6 | -0.1384 | -0.0911 |  | 1083.5 | 0.0799 | 0.0269 |
| 411.4 | 0.14 | 0.0629 |  | 558.3 | 0.1516 | 0.1149 |  | 1121.7 | -0.0501 | -0.0881 |
| 419.9 | -0.08 | -0.0781 |  | 565.9 | 0.1216 | 0.0319 |  | 1107.4 | 0.0499 | 0.0909 |
| 427.8 | 0.09 | 0.0829 |  | 565.9 | 0.0116 | 0.0319 |  | 1130.1 | 0.0799 | 0.0589 |
| 418.6 | -0.05 | -0.1091 |  | 564.5 | 0.0616 | 0.0769 |  | 1137.7 | 0.1199 | 0.0549 |
| 447.5 | 0.02 | 0.0079 |  | 563.5 | 0.1216 | 0.1079 |  | 1131.4 | 0.0499 | 0.0959 |
| 447.5 | -0.06 | 0.0079 |  | 554.7 | 0.0716 | 0.0969 |  | 1140 | 0.0099 | -0.0701 |
| 445.4 | -0.06 | -0.0741 |  | 570.4 | -0.0384 | -0.1071 |  | 1137.6 | -0.0101 | 0.0569 |
| 432.6 | 0.1 | 0.0479 |  | 581.6 | -0.0184 | 0.0079 |  | 1146.8 | 0.0699 | 0.1739 |
| 441.6 | -0.02 | -0.0061 |  | 593.2 | -0.0884 | -0.0501 |  | 1171.5 | 0.1099 | 0.0179 |
| 447 | -0.09 | -0.0231 |  | 598.1 | -0.0784 | 0.0029 |  | 1155.3 | 0.0599 | 0.1289 |
| 437.6 | 0.12 | 0.1399 |  | 596.8 | -0.0084 | -0.0551 |  | 1161.5 | 0.0099 | 0.0659 |
| 435.3 | 0.14 | 0.1559 |  | 589.9 | 0.0916 | 0.0789 |  | 1193.5 | -0.2301 | -0.1921 |
| 451.7 | 0.06 | 0.1049 |  | 589.9 | 0.0116 | 0.0789 |  | 1178 | 0.0999 | -0.0001 |
| 442.6 | -0.02 | -0.0391 |  | 590.7 | 0.0316 | 0.0629 |  | 1179.2 | 0.0999 | -0.0011 |
| 440 | 0.04 | 0.0699 |  | 588.4 | 0.0416 | 0.1009 |  | 1185.4 | 0.0099 | -0.0641 |
| 472.6 | -0.03 | 0.0049 |  | 595.3 | -0.0184 | -0.0721 |  | 1194.6 | -0.1901 | -0.1541 |
| 472.6 | -0.07 | 0.0049 |  | 577.1 | 0.0716 | 0.0669 |  | 1217.5 | -0.2801 | -0.2791 |
| 477.1 | 0.03 | 0.0849 |  | 578.6 | 0.0816 | 0.0479 |  | 1219.6 | -0.2001 | -0.2221 |
| 477.1 | 0.01 | 0.0849 |  | 594.3 | -0.0484 | -0.0651 |  | 1209.3 | 0.0099 | -0.0951 |
| 471 | -0.05 | -0.0201 |  | 605.5 | -0.0284 | -0.0051 |  | 1218.6 | -0.1701 | -0.2421 |
| 463.9 | 0.05 | 0.1029 |  | 620.7 | -0.0384 | -0.0241 |  | 1241.4 | -0.2601 | -0.2071 |
| 459.2 | 0.12 | 0.1459 |  | 615.3 | 0.1516 | 0.0979 |  | 1242.5 | -0.1701 | -0.2821 |
| 459.2 | 0.16 | 0.1459 |  | 609.9 | 0.1716 | 0.0979 |  | 1259.6 | 0.0699 | -0.0311 |
| 475.7 | 0.02 | 0.0639 |  | 613.8 | 0.0816 | 0.1399 |  | 1257.2 | -0.0101 | -0.1091 |
| 462.2 | 0.21 | 0.1479 |  | 612.4 | 0.0616 | 0.1389 |  | 1266.4 | -0.1801 | -0.1641 |
| 470.5 | -0.13 | -0.0511 |  | 611.4 | 0.1416 | 0.1219 |  | 1289.2 | 0.0799 | -0.0061 |
| 463.9 | 0.08 | 0.1089 |  | 619.3 | -0.0884 | -0.0361 |  | 1283.6 | 0.0899 | -0.0101 |
| 502.3 | 0.11 | 0.0989 |  | 619.3 | -0.0284 | -0.0361 |  | 1283.5 | 0.0599 | 0.0049 |
| 480.7 | 0.05 | 0.1119 |  | 602.6 | 0.0716 | 0.0979 |  | 1311.3 | -0.0001 | 0.0629 |
| 480.7 | 0.13 | 0.1119 |  | 629.5 | -0.0184 | 0.0069 |  | 1307.5 | 0.0599 | 0.0319 |
| 488.5 | 0.15 | 0.0799 |  | 647.2 | -0.0684 | 0.0099 |  | 1311.1 | 0.2199 | 0.1049 |
| 489.5 | -0.04 | 0.0049 |  | 644.7 | -0.0184 | -0.0481 |  | 1298.9 | 0.0999 | 0.1119 |
| 494.9 | -0.03 | -0.0731 |  | 639.3 | 0.1316 | 0.0869 |  | 1307.5 | 0.0599 | 0.0579 |
| 483.1 | 0.14 | 0.1229 |  | 628.7 | 0.0016 | 0.0109 |  | 1305.1 | 0.0099 | -0.0421 |
| 483.1 | 0.18 | 0.1229 |  | 635.8 | 0.1516 | 0.1019 |  | 1335.3 | -0.0501 | -0.0701 |
| 491.7 | -0.07 | -0.0291 |  | 637.7 | 0.1016 | 0.1619 |  | 1322.8 | 0.1099 | 0.0189 |
| 502.8 | 0.13 | 0.1219 |  | 646.1 | 0.0116 | 0.0059 |  | 1359.2 | 0.0099 | 0.0379 |
| 499.6 | 0.09 | 0.0479 |  | 636.3 | 0.0216 | 0.1099 |  | 1346.7 | 0.0899 | -0.0061 |
| 494.4 | -0.14 | -0.0771 |  | 626.8 | 0.2116 | 0.1269 |  | 1349.8 | -0.1201 | -0.1391 |
| 498.6 | -0.04 | 0.0099 |  | 643.2 | 0.0016 | -0.0291 |  | 1383.1 | -0.0001 | -0.0401 |
| 487.8 | 0.09 | 0.0739 |  | 624.9 | 0.1216 | 0.1299 |  | 1369.5 | 0.1799 | 0.1539 |
| 526.2 | 0.13 | 0.1089 |  | 626.5 | 0.0616 | 0.1219 |  | 1370.7 | 0.0999 | 0.1269 |
| 522.8 | 0.06 | 0.0009 |  | 642.2 | -0.0784 | 0.0109 |  | 1410.8 | 0.1599 | 0.0469 |
| 525 | 0.05 | 0.0819 |  | 653.4 | -0.0284 | -0.0221 |  | 1394.6 | 0.0999 | 0.0529 |
| 525 | 0.01 | 0.0819 |  | 668.6 | -0.0284 | -0.0561 |  | 1397.7 | -0.1001 | -0.0241 |
| 511.6 | 0.22 | 0.1369 |  | 663.2 | 0.1316 | 0.0929 |  | 1418.5 | 0.0899 | 0.0579 |
| 524.4 | 0.16 | 0.0799 |  | 661.7 | 0.1316 | 0.0819 |  | 1424.7 | -0.0301 | -0.1381 |
| 506.5 | 0.07 | 0.1399 |  | 661.7 | 0.0316 | 0.0819 |  | 1443.3 | 0.0999 | 0.0319 |
| 518.8 | -0.1 | -0.0541 |  | 660.2 | 0.0616 | 0.0229 |  | 1444.5 | 0.1199 | 0.0389 |
| 516.1 | 0.07 | 0.0369 |  | 656.9 | -0.1684 | -0.1171 |  | 1459.9 | 0.0499 | 0.0399 |
| 516 | 0.01 | 0.0439 |  | 650.4 | 0.0816 | 0.0979 |  | 1484.7 | 0.1999 | 0.1279 |
| 516.6 | 0.1 | 0.0159 |  | 666.1 | -0.0884 | 0.0059 |  | 1467.2 | 0.0799 | 0.0719 |
| 507.1 | 0.2 | 0.1169 |  | 677.3 | -0.0184 | 0.0099 |  | 1474.8 | 0.1099 | -0.0031 |
| 523.5 | 0.06 | 0.0209 |  | 692.5 | -0.0184 | 0.0589 |  | 1477 | 0.0499 | -0.0571 |
| 510.1 | 0.21 | 0.1779 |  | 679.1 | -0.2084 | -0.1141 |  | 1474.6 | 0.0099 | 0.0149 |
| 522.5 | -0.05 | -0.0071 |  | 687.1 | 0.1316 | 0.0729 |  | 1483.8 | 0.0699 | 0.1069 |
| 511.4 | 0.17 | 0.1759 |  | 687.5 | 0.1716 | 0.1229 |  | 1491.1 | 0.0799 | 0.1179 |
| 549 | -0.03 | 0.0279 |  | 685.6 | 0.0216 | 0.0129 |  | 1492.4 | 0.1299 | 0.1249 |
| 528.8 | 0.16 | 0.1329 |  | 695.5 | 0.0316 | 0.0769 |  | 1507.8 | 0.0699 | -0.0491 |
| 547.9 | -0.06 | 0.0029 |  | 691.1 | 0.0116 | 0.0539 |  | 1554.5 | -0.2501 | -0.1381 |
| 548.9 | 0.03 | 0.0279 |  | 681.9 | -0.0684 | -0.1551 |  | 1546.4 | 0.0099 | -0.0701 |
| 548.9 | 0 | 0.0279 |  | 680.8 | -0.1484 | -0.1381 |  | 1578.5 | -0.2801 | -0.1761 |
| 535.6 | 0.23 | 0.1559 |  | 674.4 | 0.0816 | 0.0989 |  | 1580.6 | -0.2801 | -0.2591 |
| 549.2 | -0.03 | 0.0409 |  | 689.5 | 0.2316 | 0.1519 |  | 1602.4 | -0.2801 | -0.1991 |
| 532.5 | 0.09 | 0.1339 |  | 716.5 | 0.0616 | 0.0319 |  | 1604.6 | -0.2601 | -0.1991 |
| 532.5 | 0.19 | 0.1339 |  | 716.5 | -0.0084 | 0.0289 |  | 1594.3 | 0.0199 | -0.0871 |
| 542.8 | -0.03 | -0.0171 |  | 711.1 | 0.1016 | 0.1179 |  | 1615.1 | -0.0601 | -0.1741 |
| 540.6 | 0.08 | 0.0589 |  | 709.5 | 0.0216 | 0.0019 |  | 1674.2 | 0.0599 | -0.0281 |
| 540.6 | 0.07 | 0.0589 |  | 698.5 | 0.2216 | 0.2039 |  | 1668.5 | 0.0499 | -0.0431 |
| 531 | 0.15 | 0.1179 |  | 715 | 0.0516 | 0.0979 |  | 1682.6 | 0.0699 | 0.0079 |
| 531 | 0.2 | 0.1179 |  | 742.4 | 0.0216 | 0.0749 |  | 1692.4 | 0.0499 | -0.0351 |
| 547.5 | 0.1 | 0.0259 |  | 737 | 0.0516 | 0.0389 |  | 1699.2 | 0.0999 | 0.0599 |
| 534 | 0.21 | 0.1309 |  | 735.5 | -0.0084 | -0.0181 |  | 1713.5 | -0.0001 | -0.1131 |
| 546.4 | -0.04 | -0.0241 |  | 736.2 | -0.0784 | 0.0129 |  | 1706.5 | 0.0999 | 0.1819 |
| 535.4 | 0.2 | 0.1509 |  | 724.5 | 0.2016 | 0.1429 |  | 1716.3 | 0.0599 | -0.0501 |
| 569.3 | -0.11 | -0.0881 |  | 727.6 | 0.1116 | 0.0599 |  | 1730.5 | 0.0699 | -0.0151 |
| 573 | 0.04 | -0.0041 |  | 724.3 | 0.0816 | 0.1349 |  | 1731.7 | 0.0199 | -0.0371 |
| 572.9 | 0.01 | -0.0471 |  | 751.2 | -0.0384 | 0.0589 |  | 1768.1 | 0.0099 | 0.0269 |
| 572.9 | 0.01 | -0.0471 |  | 766.3 | -0.0384 | 0.0549 |  | 1778.3 | 0.0799 | 0.0899 |
| 558.3 | 0.11 | 0.1149 |  | 745.8 | 0.1716 | 0.0839 |  | 1817.8 | -0.2301 | -0.1181 |
| 558.3 | 0.14 | 0.1149 |  | 760.2 | -0.1084 | -0.0711 |  | 1803.5 | 0.0799 | -0.0091 |
| 560.3 | 0.1 | 0.1059 |  | 748.5 | 0.1616 | 0.1149 |  | 1841.7 | -0.0501 | -0.0221 |
| 565.9 | 0.1 | 0.0319 |  | 764.9 | 0.0016 | 0.0409 |  | 1827.4 | 0.0499 | 0.0239 |
| 564.5 | 0.06 | 0.0769 |  | 748.2 | 0.0716 | 0.1319 |  | 1850.1 | 0.0799 | 0.1089 |
| 555 | 0.14 | 0.1049 |  | 753.1 | 0.0116 | -0.0661 |  | 1857.7 | 0.1199 | 0.0559 |
| 571.4 | -0.07 | -0.1041 |  | 789.7 | 0.2616 | 0.1749 |  | 1851.4 | 0.0499 | 0.1529 |
| 559.6 | 0.06 | 0.1179 |  | 777.8 | 0.1016 | 0.0379 |  | 1859.9 | 0.0099 | -0.1091 |
| 581.6 | -0.01 | 0.0079 |  | 783.4 | 0.0216 | -0.0021 |  | 1857.6 | -0.0101 | 0.0499 |
| 593.2 | -0.13 | -0.0501 |  | 781.9 | 0.0416 | -0.0211 |  | 1866.8 | 0.0699 | -0.0001 |
| 598.1 | 0.05 | 0.0029 |  | 772.4 | 0.2316 | 0.1399 |  | 1874.1 | 0.0599 | -0.0251 |
| 598.1 | -0.04 | 0.0029 |  | 776.4 | 0.0316 | 0.0979 |  | 1875.3 | 0.0599 | -0.0351 |
| 596.8 | -0.05 | -0.0551 |  | 770.6 | 0.0316 | 0.0829 |  | 1881.5 | 0.0099 | -0.0311 |
| 596.8 | -0.01 | -0.0551 |  | 772.1 | 0.0716 | 0.1279 |  | 1890.7 | -0.1401 | -0.0541 |
| 584.1 | 0.09 | 0.0249 |  | 777.1 | 0.0316 | 0.0669 |  | 1898 | 0.0999 | -0.0121 |
| 589.9 | 0.11 | 0.0789 |  | 810.6 | -0.1284 | -0.0381 |  | 1902.3 | -0.1001 | -0.1051 |
| 589.9 | 0.08 | 0.0789 |  | 812.6 | 0.1216 | 0.0299 |  | 1905.4 | 0.0099 | -0.0121 |
| 585.3 | -0.01 | 0.0699 |  | 814.2 | -0.0084 | 0.0299 |  | 1921.9 | 0.0999 | 0.0139 |
| 590.7 | 0.03 | 0.0629 |  | 810 | -0.1284 | -0.0381 |  | 1923.2 | 0.0999 | 0.0274 |
| 588.4 | 0.07 | 0.1009 |  | 793 | 0.0816 | 0.1399 |  | 1939.6 | -0.2001 | -0.0871 |
| 588.4 | 0.05 | 0.1009 |  | 807.3 | -0.0084 | -0.0811 |  | 1929.3 | 0.0099 | -0.0226 |
| 594.3 | -0.03 | -0.0651 |  | 796.3 | 0.1316 | 0.1249 |  | 1938.6 | -0.1701 | -0.1176 |
| 583.6 | 0.07 | 0.0289 |  | 802.3 | 0.0816 | 0.0209 |  | 1961.4 | -0.2601 | -0.1406 |
| 605.5 | -0.01 | -0.0051 |  | 794.5 | 0.0516 | 0.1419 |  | 1945.9 | 0.0599 | -0.0111 |
| 623.1 | 0.05 | 0.0759 |  | 796 | 0.0716 | 0.1229 |  | 1955.7 | 0.0999 | -0.0196 |
| 620.7 | -0.06 | -0.0241 |  | 823 | -0.0284 | 0.0579 |  | 1953.5 | 0.1299 | 0.0134 |
| 620.7 | -0.02 | -0.0241 |  | 834.5 | -0.1084 | -0.0621 |  | 1955.7 | 0.0599 | -0.0316 |
| 609.9 | 0.12 | 0.0979 |  | 838.1 | -0.0184 | 0.0309 |  | 1953.3 | 0.0199 | 0.0044 |
| 609.9 | 0.16 | 0.0979 |  | 832 | -0.0984 | -0.1051 |  | 1962.5 | -0.1701 | -0.1196 |
| 613.8 | 0.12 | 0.1399 |  | 828.2 | 0.0416 | -0.0341 |  | 1985.3 | -0.0101 | -0.1001 |
| 613.8 | 0.08 | 0.1399 |  | 836.7 | 0.0016 | -0.0071 |  | 1969.8 | 0.0699 | 0.0159 |
| 612.4 | 0.06 | 0.1389 |  | 818.5 | 0.0416 | 0.0489 |  | 1977.4 | 0.1199 | 0.0334 |
| 612.4 | 0.06 | 0.1389 |  | 835.7 | -0.0984 | -0.0411 |  | 1979.6 | 0.0699 | 0.0114 |
| 619.3 | -0.07 | -0.0361 |  | 824.9 | -0.0384 | 0.0479 |  | 1977.2 | -0.0101 | 0.0344 |
| 618.2 | -0.03 | 0.0449 |  | 858.5 | -0.0984 | -0.0421 |  | 1986.4 | -0.1801 | -0.1061 |
| 647.2 | -0.04 | 0.0099 |  | 862.1 | 0.0016 | 0.0489 |  | 2009.2 | 0.0799 | -0.0396 |
| 644.7 | 0 | -0.0481 |  | 840.9 | 0.0716 | 0.0689 |  | 1993.7 | 0.0799 | 0.0399 |
| 644.7 | -0.01 | -0.0481 |  | 855.1 | -0.0684 | -0.0511 |  | 2003.6 | 0.0899 | 0.0434 |
| 641 | 0 | 0.0429 |  | 855.9 | -0.0784 | -0.0571 |  | 1995 | 0.1099 | 0.0039 |
| 635.8 | 0.11 | 0.1019 |  | 853.7 | 0.0616 | -0.0081 |  | 2003.5 | 0.0599 | 0.0479 |
| 635.8 | 0.14 | 0.1019 |  | 860.6 | -0.0484 | 0.0119 |  | 2001.1 | 0.0099 | 0.0089 |
| 637.7 | 0.11 | 0.1619 |  | 860.6 | 0.0516 | 0.0119 |  | 2031.3 | -0.0001 | -0.0151 |
| 637.7 | 0.09 | 0.1619 |  | 842.4 | 0.0316 | 0.0859 |  | 2027.5 | 0.0599 | 0.0804 |
| 646.1 | 0.02 | 0.0059 |  | 843.9 | 0.0716 | 0.0939 |  | 2025.2 | 0.1199 | 0.0119 |
| 636.3 | 0.07 | 0.1099 |  | 859.6 | -0.0984 | -0.0231 |  | 2018.9 | 0.0999 | 0.0674 |
| 636.3 | 0.06 | 0.1099 |  | 886 | -0.0184 | 0.0409 |  | 2027.5 | 0.0599 | 0.0679 |
| 626.8 | 0.08 | 0.1269 |  | 880.6 | 0.0916 | 0.0179 |  | 2025.1 | 0.0099 | 0.0079 |
| 626.8 | 0.18 | 0.1269 |  | 870.1 | 0.0516 | 0.0749 |  | 2059 | 0.1799 | 0.0889 |
| 624.9 | 0.1 | 0.1299 |  | 873.5 | 0.0916 | 0.1239 |  | 2055.3 | -0.0501 | 0.0609 |
| 642.2 | -0.05 | 0.0109 |  | 879.1 | 0.0216 | 0.0719 |  | 2042.8 | 0.1099 | 0.0749 |
| 653.4 | -0.01 | -0.0221 |  | 874.5 | 0.2016 | 0.1299 |  | 2045.9 | -0.0401 | -0.0861 |
| 671.1 | -0.04 | -0.0311 |  | 877.6 | 0.0916 | 0.1119 |  | 2079.2 | 0.0099 | 0.0569 |
| 668.6 | 0 | -0.0561 |  | 879.9 | 0.0416 | 0.0249 |  | 2075.4 | 0.0399 | -0.0601 |
| 668.6 | -0.01 | -0.0561 |  | 884.5 | 0.0416 | 0.0679 |  | 2078.9 | 0.2099 | 0.1219 |
| 664.9 | 0.08 | 0.0569 |  | 866.3 | 0.0216 | 0.0669 |  | 2075.3 | 0.0399 | -0.0751 |
| 661.7 | 0.06 | 0.0819 |  | 867.9 | 0.0716 | 0.0879 |  | 2072.9 | 0.0099 | -0.0551 |
| 661.7 | 0.11 | 0.0819 |  | 894.8 | -0.0484 | -0.0111 |  | 2103.1 | -0.0001 | -0.0731 |
| 661.7 | 0.1 | 0.0819 |  | 906.3 | -0.1284 | -0.0881 |  | 2089.4 | 0.1799 | 0.1269 |
| 660.2 | 0.06 | 0.0229 |  | 909.9 | -0.0184 | -0.0091 |  | 2090.7 | 0.0999 | 0.1319 |
| 660.2 | 0.06 | 0.0229 |  | 889.3 | 0.1416 | 0.0839 |  | 2114.6 | 0.0999 | -0.0041 |
| 659.3 | -0.09 | -0.0181 |  | 901.6 | 0.0916 | 0.0059 |  | 2117.7 | -0.1001 | -0.0471 |
| 667.1 | 0.05 | -0.0251 |  | 905.8 | -0.0084 | -0.0731 |  | 2138.5 | 0.0899 | 0.0089 |
| 656.9 | -0.12 | -0.1171 |  | 890.3 | 0.0416 | 0.0589 |  | 2144.7 | -0.0301 | -0.0881 |
| 666.1 | -0.06 | 0.0059 |  | 891.8 | 0.0716 | 0.0529 |  |  |  |  |
| 677.3 | -0.01 | 0.0099 |  | 907.5 | -0.1184 | -0.0521 |  |  |  |  |
| 690.9 | 0.21 | 0.1319 |  | 918.7 | -0.0384 | -0.0321 |  |  |  |  |
| 692.5 | 0.03 | 0.0589 |  | 930.3 | -0.1284 | -0.0581 |  |  |  |  |
| 692.5 | 0 | 0.0589 |  | 933.9 | -0.0084 | -0.0061 |  |  |  |  |
| 683 | -0.02 | -0.0491 |  | 912.7 | 0.1216 | 0.0279 |  |  |  |  |
| 687.5 | 0.11 | 0.1229 |  | 925.5 | 0.0816 | 0.0239 |  |  |  |  |
| 687.5 | 0.14 | 0.1229 |  | 932.4 | -0.0684 | -0.0391 |  |  |  |  |
| 674.6 | 0.13 | 0.0799 |  | 915.7 | 0.0716 | 0.0089 |  |  |  |  |
| 691.1 | 0.08 | 0.0539 |  | 931.4 | -0.1184 | -0.0531 |  |  |  |  |
| 680.8 | -0.13 | -0.1381 |  | 942.6 | -0.0384 | -0.0621 |  |  |  |  |
| 719 | 0.04 | 0.0199 |  | 954.2 | -0.0884 | -0.0041 |  |  |  |  |
| 716.5 | 0 | 0.0289 |  | 957.8 | -0.0084 | 0.0229 |  |  |  |  |
| 716.5 | 0 | 0.0289 |  | 950.9 | -0.0084 | 0.0259 |  |  |  |  |
| 719.1 | 0.1 | 0.0389 |  | 949.4 | 0.0716 | 0.0499 |  |  |  |  |
| 704.9 | -0.03 | -0.0841 |  | 938.1 | 0.0616 | 0.0539 |  |  |  |  |
| 698.5 | 0.19 | 0.2039 |  | 939.6 | 0.0616 | 0.0289 |  |  |  |  |
| 707.1 | -0.09 | -0.1001 |  | 955.3 | -0.0884 | -0.0041 |  |  |  |  |
| 715 | 0.04 | 0.0979 |  | 966.5 | -0.0384 | -0.0021 |  |  |  |  |
| 704.7 | -0.15 | -0.0751 |  | 976.4 | 0.1216 | 0.1879 |  |  |  |  |
| 703.2 | 0.07 | -0.0081 |  | 981.1 | 0.2516 | 0.1719 |  |  |  |  |
| 721 | -0.02 | 0.0519 |  | 969.2 | 0.1016 | 0.0139 |  |  |  |  |
| 742.4 | 0 | 0.0749 |  | 972.5 | 0.2416 | 0.1619 |  |  |  |  |
| 742.4 | 0.02 | 0.0749 |  | 973.4 | 0.0816 | 0.1759 |  |  |  |  |
| 721.2 | 0.04 | 0.0609 |  | 972.4 | 0.1216 | 0.1799 |  |  |  |  |
| 736.2 | -0.05 | 0.0129 |  | 982.4 | 0.2716 | 0.1759 |  |  |  |  |
| 724.5 | 0.17 | 0.1429 |  | 962.1 | 0.0316 | 0.1229 |  |  |  |  |
| 727.6 | 0.08 | 0.0599 |  | 963.6 | 0.0316 | 0.0729 |  |  |  |  |
| 733.1 | -0.16 | -0.1041 |  | 978.8 | 0.2216 | 0.1789 |  |  |  |  |
| 740.9 | 0.09 | 0.1089 |  | 990.5 | -0.0484 | 0.0429 |  |  |  |  |
| 751.2 | -0.01 | 0.0589 |  | 1005.7 | 0.0116 | 0.0259 |  |  |  |  |
| 766.3 | -0.03 | 0.0549 |  | 1000.3 | 0.1216 | 0.0909 |  |  |  |  |
| 762.7 | 0.02 | 0.0059 |  | 997.3 | 0.0516 | 0.0809 |  |  |  |  |
| 745.2 | 0.03 | 0.0919 |  | 987.8 | 0.1716 | 0.1659 |  |  |  |  |
| 745.8 | 0.08 | 0.0839 |  | 1004.2 | 0.0116 | 0.0249 |  |  |  |  |
| 745.8 | 0.15 | 0.0839 |  | 987.5 | 0.0616 | 0.1419 |  |  |  |  |
| 760.2 | -0.1 | -0.0711 |  | 1029.6 | 0.0516 | -0.0101 |  |  |  |  |
| 760.2 | -0.05 | -0.0711 |  | 1014.4 | -0.0184 | -0.0801 |  |  |  |  |
| 745.4 | 0.08 | 0.0819 |  | 1026 | -0.0984 | -0.0181 |  |  |  |  |
| 748.5 | 0.13 | 0.1119 |  | 1029.6 | 0.0316 | -0.0221 |  |  |  |  |
| 748.5 | 0.15 | 0.1149 |  | 1016.3 | -0.2084 | -0.1121 |  |  |  |  |
| 750.7 | 0.12 | 0.0879 |  | 1024.2 | 0.1116 | 0.0199 |  |  |  |  |
| 764.9 | 0.09 | 0.0409 |  | 1022.7 | 0.0016 | -0.0201 |  |  |  |  |
| 746.7 | 0.05 | 0.1039 |  | 1023.4 | -0.0984 | -0.0031 |  |  |  |  |
| 759.7 | -0.13 | -0.0801 |  | 1021.2 | 0.0616 | -0.0221 |  |  |  |  |
| 754.6 | -0.17 | -0.1101 |  | 1014.8 | -0.0884 | -0.0751 |  |  |  |  |
| 788.7 | 0.24 | 0.1679 |  | 1028.2 | -0.0484 | -0.0141 |  |  |  |  |
| 786.6 | 0.02 | 0.0759 |  | 1028.2 | 0.0216 | -0.0141 |  |  |  |  |
| 789.7 | 0.2 | 0.1749 |  | 1009.9 | 0.0516 | 0.0879 |  |  |  |  |
| 769.1 | 0.05 | 0.0699 |  | 1011.4 | 0.0616 | 0.0649 |  |  |  |  |
| 769.7 | 0.1 | 0.0879 |  | 1027.1 | -0.0984 | -0.0361 |  |  |  |  |
| 777.8 | 0.09 | 0.0379 |  | 1042 | -0.0884 | -0.1141 |  |  |  |  |
| 778.8 | -0.01 | 0.0149 |  | 1053.5 | -0.0184 | -0.0541 |  |  |  |  |
| 781.9 | 0.06 | -0.0211 |  | 1048.2 | 0.1116 | 0.0279 |  |  |  |  |
| 781.9 | 0.04 | -0.0211 |  | 1046.6 | 0.0316 | -0.0001 |  |  |  |  |
| 772.4 | 0.1 | 0.1419 |  | 1047.4 | -0.0284 | 0.0339 |  |  |  |  |
| 772.4 | 0.19 | 0.1399 |  | 1035.6 | 0.1916 | 0.1889 |  |  |  |  |
| 790.9 | 0.2 | 0.1369 |  | 1052.1 | -0.0184 | 0.0159 |  |  |  |  |
| 790.9 | 0.21 | 0.1369 |  | 1035.4 | 0.0716 | 0.1699 |  |  |  |  |
| 776.4 | 0.06 | 0.0979 |  | 1062.3 | -0.0184 | -0.0791 |  |  |  |  |
| 770.6 | 0.04 | 0.0829 |  | 1077.5 | -0.0384 | -0.0101 |  |  |  |  |
| 814.2 | 0.03 | 0.0299 |  | 1064.1 | -0.2084 | -0.1981 |  |  |  |  |
| 814.2 | 0.01 | 0.0299 |  | 1071.3 | -0.1284 | -0.0361 |  |  |  |  |
| 810 | -0.1 | -0.0381 |  | 1076 | -0.0384 | -0.0411 |  |  |  |  |
| 793 | 0.1 | 0.1399 |  | 1076 | -0.0184 | -0.0411 |  |  |  |  |
| 793.6 | 0.09 | 0.1519 |  | 1065.7 | -0.2084 | -0.2111 |  |  |  |  |
| 801.7 | 0.09 | 0.0289 |  | 1059.3 | 0.0716 | -0.0181 |  |  |  |  |
| 802.7 | -0.01 | 0.0059 |  | 1075 | -0.0884 | -0.0591 |  |  |  |  |
| 796.3 | 0.13 | 0.1239 |  | 1086.2 | -0.0184 | -0.0441 |  |  |  |  |
| 796.3 | 0.11 | 0.1249 |  | 1101.4 | -0.0084 | 0.0379 |  |  |  |  |
| 802.4 | 0.05 | 0.0229 |  | 1088 | -0.2084 | -0.1201 |  |  |  |  |
| 812.7 | 0.1 | 0.0289 |  | 1083.7 | -0.0284 | -0.0031 |  |  |  |  |
| 807.5 | -0.15 | -0.0801 |  | 1095.2 | -0.1084 | -0.0431 |  |  |  |  |
| 811.7 | -0.07 | 0.0039 |  | 1099.9 | -0.0284 | 0.0639 |  |  |  |  |
| 801 | 0.09 | 0.0689 |  | 1089.7 | -0.1984 | -0.1521 |  |  |  |  |
| 823 | -0.01 | 0.0579 |  | 1083.2 | 0.0716 | 0.0159 |  |  |  |  |
| 834.5 | -0.13 | -0.0621 |  | 1110.1 | -0.0684 | 0.0229 |  |  |  |  |
| 838.1 | 0 | 0.0309 |  | 1121.7 | -0.1084 | -0.0761 |  |  |  |  |
| 838.1 | -0.01 | 0.0309 |  | 1125.3 | 0.0116 | -0.0561 |  |  |  |  |
| 817 | 0.03 | 0.0489 |  | 1121.1 | -0.1384 | -0.1341 |  |  |  |  |
| 817.5 | 0.09 | 0.0459 |  | 1119.2 | -0.1684 | -0.1651 |  |  |  |  |
| 826.6 | -0.04 | 0.0139 |  | 1107.4 | 0.1516 | 0.0909 |  |  |  |  |
| 832 | -0.13 | -0.1051 |  | 1123.9 | -0.0484 | -0.0751 |  |  |  |  |
| 832 | -0.07 | -0.1051 |  | 1123.9 | -0.0184 | -0.0751 |  |  |  |  |
| 820.3 | 0.11 | 0.0419 |  | 1118.7 | -0.2084 | -0.1581 |  |  |  |  |
| 828.3 | 0.05 | -0.0331 |  | 1107.2 | 0.0616 | 0.0739 |  |  |  |  |
| 836.7 | 0.02 | -0.0071 |  | 1122.8 | -0.0684 | -0.0731 |  |  |  |  |
| 818.5 | 0.04 | 0.0489 |  | 1122.8 | -0.1684 | -0.0731 |  |  |  |  |
| 831.5 | -0.15 | -0.0981 |  | 1149.3 | 0.0616 | 0.1079 |  |  |  |  |
| 835.7 | -0.07 | -0.0411 |  | 1143.9 | 0.1016 | 0.0259 |  |  |  |  |
| 824.9 | 0.07 | 0.0479 |  | 1148.7 | 0.2216 | 0.1339 |  |  |  |  |
| 841.3 | 0.04 | 0.0809 |  | 1136.7 | 0.0816 | 0.0739 |  |  |  |  |
| 862.1 | 0.02 | 0.0489 |  | 1142.3 | 0.0416 | -0.0171 |  |  |  |  |
| 862.1 | 0.01 | 0.0489 |  | 1143.1 | -0.1184 | -0.0291 |  |  |  |  |
| 857.8 | -0.11 | -0.0461 |  | 1131.2 | -0.0084 | 0.0739 |  |  |  |  |
| 840.9 | 0.03 | 0.0689 |  | 1140.9 | 0.0416 | -0.0441 |  |  |  |  |
| 850.5 | 0.01 | 0.0749 |  | 1131.4 | 0.1716 | 0.0959 |  |  |  |  |
| 855.9 | -0.11 | -0.0571 |  | 1138.6 | -0.0784 | -0.0371 |  |  |  |  |
| 855.9 | -0.04 | -0.0571 |  | 1131.1 | 0.0716 | 0.0929 |  |  |  |  |
| 841.1 | 0.15 | 0.0769 |  | 1169.6 | -0.1184 | -0.0851 |  |  |  |  |
| 853.7 | 0.06 | -0.0081 |  | 1173.2 | -0.0084 | 0.0459 |  |  |  |  |
| 844.2 | 0.14 | 0.0919 |  | 1168.9 | -0.1484 | -0.0771 |  |  |  |  |
| 844.2 | 0.17 | 0.0919 |  | 1152.6 | 0.1616 | 0.2039 |  |  |  |  |
| 860.6 | 0.06 | 0.0119 |  | 1167 | -0.1384 | -0.1751 |  |  |  |  |
| 860.6 | -0.03 | 0.0119 |  | 1155.3 | 0.1716 | 0.1289 |  |  |  |  |
| 842.4 | 0.04 | 0.0859 |  | 1155.3 | 0.1916 | 0.1289 |  |  |  |  |
| 859.6 | -0.08 | -0.0231 |  | 1162.6 | -0.0484 | 0.0079 |  |  |  |  |
| 848.9 | 0.09 | 0.0889 |  | 1166.5 | -0.1884 | -0.1481 |  |  |  |  |
| 848.5 | 0.15 | 0.0839 |  | 1170.7 | -0.1084 | -0.0211 |  |  |  |  |
| 866.3 | 0.07 | 0.0749 |  | 1181.9 | -0.0484 | -0.0011 |  |  |  |  |
| 886 | 0 | 0.0409 |  | 1193.5 | -0.1584 | -0.1921 |  |  |  |  |
| 886 | 0 | 0.0409 |  | 1197.1 | -0.0184 | -0.0781 |  |  |  |  |
| 865.4 | 0.11 | 0.0599 |  | 1193.5 | -0.2284 | -0.2251 |  |  |  |  |
| 877.6 | 0.05 | 0.1119 |  | 1183.9 | 0.0516 | -0.0071 |  |  |  |  |
| 877.6 | 0.08 | 0.1119 |  | 1195.7 | -0.0584 | -0.1321 |  |  |  |  |
| 884.5 | 0.07 | 0.0679 |  | 1190.5 | -0.1884 | -0.2481 |  |  |  |  |
| 866.3 | 0.04 | 0.0669 |  | 1179 | 0.0716 | 0.0049 |  |  |  |  |
| 872.8 | 0.08 | 0.1109 |  | 1194.6 | -0.0784 | -0.1591 |  |  |  |  |
| 872.5 | 0.12 | 0.1109 |  | 1192.1 | -0.2584 | -0.2581 |  |  |  |  |
| 894.8 | -0.03 | -0.0111 |  | 1205.9 | -0.0284 | -0.0361 |  |  |  |  |
| 906.3 | -0.14 | -0.0881 |  | 1217.5 | -0.1884 | -0.2791 |  |  |  |  |
| 891.4 | 0.08 | 0.0589 |  | 1219.4 | -0.1684 | -0.2251 |  |  |  |  |
| 909.9 | 0 | -0.0091 |  | 1211.7 | -0.1984 | -0.1421 |  |  |  |  |
| 909.9 | 0 | -0.0091 |  | 1214.4 | -0.1984 | -0.2581 |  |  |  |  |
| 889.3 | 0.06 | 0.0839 |  | 1218.6 | -0.3084 | -0.2371 |  |  |  |  |
| 889.3 | 0.14 | 0.0839 |  | 1216 | -0.3584 | -0.2871 |  |  |  |  |
| 903.8 | -0.1 | -0.0531 |  | 1241.4 | -0.1884 | -0.2071 |  |  |  |  |
| 901.6 | 0.06 | 0.0059 |  | 1241.4 | -0.1184 | -0.2071 |  |  |  |  |
| 901.6 | 0.07 | 0.0059 |  | 1231.6 | -0.2084 | -0.1571 |  |  |  |  |
| 892.1 | 0.1 | 0.0389 |  | 1238.8 | -0.1884 | -0.1941 |  |  |  |  |
| 890.3 | 0.05 | 0.0589 |  | 1238.3 | -0.2084 | -0.1981 |  |  |  |  |
| 907.5 | -0.07 | -0.0521 |  | 1242.5 | -0.3384 | -0.2721 |  |  |  |  |
| 918.7 | -0.02 | -0.0321 |  | 1265.3 | -0.1784 | -0.1501 |  |  |  |  |
| 930.3 | -0.14 | -0.0581 |  | 1265.3 | -0.1184 | -0.1501 |  |  |  |  |
| 933.9 | 0.01 | -0.0061 |  | 1255.6 | -0.2084 | -0.1631 |  |  |  |  |
| 933.9 | 0 | -0.0061 |  | 1262 | -0.0084 | -0.0761 |  |  |  |  |
| 913.3 | 0.07 | 0.0209 |  | 1262.8 | -0.0684 | -0.1001 |  |  |  |  |
| 921.4 | 0.07 | 0.0039 |  | 1266.4 | -0.0784 | -0.1671 |  |  |  |  |
| 922.3 | -0.02 | 0.0239 |  | 1292.9 | 0.0116 | -0.0001 |  |  |  |  |
| 927.7 | -0.08 | -0.0031 |  | 1279.5 | -0.2184 | -0.1971 |  |  |  |  |
| 925.5 | 0.05 | 0.0239 |  | 1285.9 | 0.1216 | 0.0229 |  |  |  |  |
| 925.5 | 0.07 | 0.0239 |  | 1285.9 | 0.0116 | 0.0229 |  |  |  |  |
| 931.7 | -0.06 | -0.0501 |  | 1284.5 | 0.0616 | 0.0299 |  |  |  |  |
| 932.4 | 0.03 | -0.0391 |  | 1291.4 | 0.0116 | -0.0051 |  |  |  |  |
| 932.4 | -0.04 | -0.0391 |  | 1290.4 | -0.0384 | -0.0271 |  |  |  |  |
| 914.2 | 0.03 | 0.0259 |  | 1301.6 | -0.0184 | -0.0171 |  |  |  |  |
| 931.4 | -0.08 | -0.0531 |  | 1313.2 | -0.0884 | -0.0181 |  |  |  |  |
| 920.7 | 0.06 | -0.0071 |  | 1318.1 | -0.0784 | -0.0421 |  |  |  |  |
| 942.6 | -0.02 | -0.0621 |  | 1316.8 | -0.0084 | -0.0481 |  |  |  |  |
| 957.8 | 0.02 | 0.0229 |  | 1311.4 | 0.1416 | 0.0619 |  |  |  |  |
| 957.8 | 0.01 | 0.0229 |  | 1309.9 | 0.0916 | 0.1079 |  |  |  |  |
| 936.6 | 0.08 | 0.0779 |  | 1309.9 | 0.0116 | 0.1079 |  |  |  |  |
| 936.6 | 0.08 | 0.0779 |  | 1310.7 | 0.0316 | 0.0639 |  |  |  |  |
| 937.2 | 0.09 | 0.0649 |  | 1308.4 | 0.0416 | 0.0929 |  |  |  |  |
| 946.3 | -0.02 | -0.0111 |  | 1315.3 | -0.0184 | -0.0631 |  |  |  |  |
| 949.4 | 0.03 | 0.0499 |  | 1297.1 | 0.0716 | 0.1309 |  |  |  |  |
| 949.4 | 0.09 | 0.0499 |  | 1298.6 | 0.0816 | 0.0929 |  |  |  |  |
| 956.4 | 0.06 | -0.0231 |  | 1314.3 | -0.0484 | -0.0611 |  |  |  |  |
| 938.1 | 0.06 | 0.0539 |  | 1337.1 | -0.1784 | -0.0821 |  |  |  |  |
| 955.3 | -0.05 | -0.0041 |  | 1337.1 | -0.1284 | -0.0821 |  |  |  |  |
| 966.5 | -0.02 | -0.0021 |  | 1343.1 | -0.0484 | -0.0451 |  |  |  |  |
| 966.7 | 0.02 | 0.0069 |  | 1340.7 | -0.0384 | -0.1321 |  |  |  |  |
| 980.1 | 0.22 | 0.1509 |  | 1327.3 | -0.2084 | -0.2751 |  |  |  |  |
| 981.1 | 0.21 | 0.1719 |  | 1333.8 | -0.0084 | -0.0711 |  |  |  |  |
| 960.5 | 0.03 | 0.1129 |  | 1334.6 | -0.0584 | -0.0741 |  |  |  |  |
| 961.1 | 0.09 | 0.1019 |  | 1339.3 | -0.0884 | -0.1281 |  |  |  |  |
| 961.1 | 0.15 | 0.1019 |  | 1339.3 | -0.0284 | -0.1281 |  |  |  |  |
| 969.2 | 0.03 | 0.0139 |  | 1329 | -0.2384 | -0.2681 |  |  |  |  |
| 974.8 | 0.13 | 0.1989 |  | 1322.6 | 0.0716 | -0.0001 |  |  |  |  |
| 972.5 | 0.23 | 0.1619 |  | 1338.2 | -0.0684 | -0.1171 |  |  |  |  |
| 970.2 | -0.01 | 0.0409 |  | 1325.9 | -0.2884 | -0.2061 |  |  |  |  |
| 982.4 | 0.22 | 0.1759 |  | 1349.5 | -0.0184 | -0.1141 |  |  |  |  |
| 982.4 | 0.23 | 0.1759 |  | 1364.7 | -0.0184 | 0.0459 |  |  |  |  |
| 968.5 | 0.07 | 0.0259 |  | 1359.3 | 0.1316 | 0.0559 |  |  |  |  |
| 982.2 | 0.22 | 0.1619 |  | 1348.7 | 0.0016 | -0.0731 |  |  |  |  |
| 990.5 | -0.02 | 0.0429 |  | 1357.7 | -0.0284 | -0.0031 |  |  |  |  |
| 991.8 | 0.04 | -0.0261 |  | 1366.1 | 0.0116 | 0.0679 |  |  |  |  |
| 1005.7 | 0.02 | 0.0259 |  | 1363.2 | 0.0016 | 0.0409 |  |  |  |  |
| 1005.7 | 0.02 | 0.0259 |  | 1344.9 | 0.1216 | 0.0309 |  |  |  |  |
| 985.1 | 0.13 | 0.1999 |  | 1346.5 | 0.0616 | -0.0211 |  |  |  |  |
| 994.1 | 0.01 | -0.0501 |  | 1373.4 | -0.0284 | 0.0099 |  |  |  |  |
| 997.3 | 0.05 | 0.0809 |  | 1385 | -0.0884 | -0.0041 |  |  |  |  |
| 997.3 | 0.07 | 0.0809 |  | 1388.6 | -0.0284 | -0.0701 |  |  |  |  |
| 987.8 | 0.12 | 0.1659 |  | 1375.2 | -0.2184 | -0.1511 |  |  |  |  |
| 987.8 | 0.14 | 0.1659 |  | 1370.7 | 0.2216 | 0.1269 |  |  |  |  |
| 1004.2 | 0.06 | 0.0249 |  | 1373.8 | 0.0516 | -0.0011 |  |  |  |  |
| 1014.4 | 0 | -0.0801 |  | 1379.3 | -0.1884 | -0.2231 |  |  |  |  |
| 1029.6 | 0.05 | -0.0221 |  | 1376.9 | -0.1684 | -0.2251 |  |  |  |  |
| 1029.6 | 0.03 | -0.0221 |  | 1370.4 | 0.0816 | 0.1599 |  |  |  |  |
| 1025.3 | -0.1 | -0.0231 |  | 1386.1 | -0.0884 | -0.0161 |  |  |  |  |
| 1009 | 0.06 | 0.0989 |  | 1397.3 | -0.0184 | -0.0101 |  |  |  |  |
| 1009 | 0.13 | 0.0989 |  | 1412.5 | -0.0184 | 0.0239 |  |  |  |  |
| 1023.4 | -0.06 | -0.0031 |  | 1399.1 | -0.2084 | -0.1191 |  |  |  |  |
| 1020.3 | -0.1 | -0.0441 |  | 1406.4 | -0.0384 | -0.0571 |  |  |  |  |
| 1028.2 | 0.06 | -0.0141 |  | 1415.5 | 0.0316 | 0.0089 |  |  |  |  |
| 1028.2 | -0.05 | -0.0141 |  | 1411.1 | 0.0116 | 0.0119 |  |  |  |  |
| 1009.9 | 0.04 | 0.0879 |  | 1405.8 | -0.1984 | -0.1061 |  |  |  |  |
| 1017.9 | -0.17 | -0.1121 |  | 1400.8 | -0.1484 | -0.1821 |  |  |  |  |
| 1027.1 | -0.06 | -0.0341 |  | 1394.3 | 0.0816 | 0.0389 |  |  |  |  |
| 1033.4 | 0.17 | 0.1159 |  | 1421.2 | -0.0384 | 0.0539 |  |  |  |  |
| 1042 | -0.03 | -0.1141 |  | 1432.8 | -0.1184 | -0.0711 |  |  |  |  |
| 1042 | -0.05 | -0.1141 |  | 1439 | -0.0884 | -0.0131 |  |  |  |  |
| 1053.5 | 0.03 | -0.0541 |  | 1436.5 | -0.0084 | -0.0291 |  |  |  |  |
| 1053.5 | -0.01 | -0.0541 |  | 1430.3 | -0.0884 | -0.1601 |  |  |  |  |
| 1032.9 | 0.08 | 0.0389 |  | 1435 | 0.0516 | -0.0251 |  |  |  |  |
| 1042 | -0.03 | -0.0781 |  | 1429.8 | -0.1984 | -0.1741 |  |  |  |  |
| 1047.4 | 0 | 0.0339 |  | 1424.7 | -0.2284 | -0.1381 |  |  |  |  |
| 1035.6 | 0.11 | 0.1889 |  | 1418.3 | 0.0716 | 0.0699 |  |  |  |  |
| 1052.1 | 0.04 | 0.0159 |  | 1434 | -0.0884 | -0.0171 |  |  |  |  |
| 1052.1 | -0.02 | 0.0159 |  | 1447.2 | -0.0384 | -0.0261 |  |  |  |  |
| 1051.1 | -0.05 | 0.0259 |  | 1462.4 | 0.0216 | 0.0369 |  |  |  |  |
| 1040.3 | 0.06 | -0.0201 |  | 1449.1 | -0.2184 | -0.1401 |  |  |  |  |
| 1077.5 | -0.01 | -0.0031 |  | 1457 | 0.0516 | 0.0119 |  |  |  |  |
| 1062.3 | 0 | -0.0791 |  | 1455.5 | -0.0084 | -0.0911 |  |  |  |  |
| 1067.1 | -0.3 | -0.2391 |  | 1456.2 | -0.0784 | -0.0481 |  |  |  |  |
| 1077.5 | 0.01 | -0.0101 |  | 1442.8 | 0.0116 | 0.0399 |  |  |  |  |
| 1077.5 | 0 | -0.0101 |  | 1444.3 | 0.0816 | 0.0259 |  |  |  |  |
| 1073.8 | 0 | 0.0139 |  | 1473.8 | 0.0916 | 0.0499 |  |  |  |  |
| 1071.3 | -0.09 | -0.0361 |  | 1480.2 | -0.1084 | -0.0461 |  |  |  |  |
| 1071.3 | -0.09 | -0.0361 |  | 1478 | 0.0216 | -0.0761 |  |  |  |  |
| 1076 | 0.03 | -0.0411 |  | 1468.5 | 0.1616 | 0.1059 |  |  |  |  |
| 1076 | -0.02 | -0.0411 |  | 1466.7 | -0.0084 | 0.0729 |  |  |  |  |
| 1057.8 | 0.06 | 0.0369 |  | 1468.2 | 0.0716 | 0.1089 |  |  |  |  |
| 1070.8 | -0.13 | -0.0721 |  | 1473.1 | 0.0116 | 0.0209 |  |  |  |  |
| 1065.7 | -0.13 | -0.2111 |  | 1506.7 | -0.1284 | -0.0821 |  |  |  |  |
| 1075 | -0.06 | -0.0591 |  | 1510.3 | -0.0284 | -0.0411 |  |  |  |  |
| 1086.2 | 0 | -0.0441 |  | 1506 | -0.1384 | -0.0721 |  |  |  |  |
| 1101.4 | 0.01 | 0.0379 |  | 1504.1 | -0.1384 | -0.1291 |  |  |  |  |
| 1101.4 | 0 | 0.0379 |  | 1496.4 | 0.0316 | 0.0729 |  |  |  |  |
| 1083.7 | -0.06 | -0.0031 |  | 1508.8 | -0.0484 | 0.0089 |  |  |  |  |
| 1095.2 | -0.06 | -0.0431 |  | 1508.8 | -0.0184 | 0.0089 |  |  |  |  |
| 1092.1 | -0.09 | -0.1671 |  | 1490.6 | 0.0316 | 0.1029 |  |  |  |  |
| 1103.2 | 0.11 | 0.0359 |  | 1503.6 | -0.1984 | -0.1181 |  |  |  |  |
| 1099.9 | 0.06 | 0.0639 |  | 1492.1 | 0.0716 | 0.1509 |  |  |  |  |
| 1081.7 | 0.07 | -0.0101 |  | 1507.8 | -0.0884 | -0.0491 |  |  |  |  |
| 1089.7 | -0.13 | -0.1521 |  | 1497.1 | 0.0316 | 0.0029 |  |  |  |  |
| 1086.7 | -0.11 | -0.0761 |  | 1497.1 | -0.0384 | 0.0029 |  |  |  |  |
| 1110.1 | -0.04 | 0.0229 |  | 1519 | -0.0484 | -0.0411 |  |  |  |  |
| 1121.7 | -0.13 | -0.0761 |  | 1530.6 | -0.1984 | -0.1271 |  |  |  |  |
| 1125.3 | 0.01 | -0.0561 |  | 1530.6 | -0.1284 | -0.1271 |  |  |  |  |
| 1104.7 | 0.09 | 0.0239 |  | 1534.2 | -0.0084 | -0.1061 |  |  |  |  |
| 1119.2 | -0.1 | -0.1611 |  | 1530 | -0.1284 | -0.1491 |  |  |  |  |
| 1119.2 | -0.1 | -0.1651 |  | 1514.6 | 0.0516 | -0.0271 |  |  |  |  |
| 1107.4 | 0.11 | 0.0909 |  | 1527.5 | -0.1984 | -0.1931 |  |  |  |  |
| 1107.4 | 0.13 | 0.0909 |  | 1522.5 | -0.1984 | -0.1401 |  |  |  |  |
| 1116 | -0.11 | -0.1871 |  | 1531.7 | -0.0984 | -0.1011 |  |  |  |  |
| 1123.9 | 0 | -0.0751 |  | 1521 | -0.0384 | -0.0941 |  |  |  |  |
| 1118.7 | -0.15 | -0.1581 |  | 1543 | -0.0284 | -0.0781 |  |  |  |  |
| 1113.6 | -0.19 | -0.1391 |  | 1554.5 | -0.1984 | -0.1381 |  |  |  |  |
| 1122.8 | -0.05 | -0.0731 |  | 1554.5 | -0.1084 | -0.1381 |  |  |  |  |
| 1142.4 | -0.02 | 0.0039 |  | 1558.1 | -0.0184 | -0.0421 |  |  |  |  |
| 1142.4 | -0.07 | 0.0039 |  | 1552 | -0.0984 | -0.1481 |  |  |  |  |
| 1128.7 | 0.07 | 0.0139 |  | 1556.7 | 0.0016 | -0.0661 |  |  |  |  |
| 1136.7 | 0.1 | 0.0739 |  | 1551.5 | -0.1984 | -0.1701 |  |  |  |  |
| 1143.1 | -0.09 | -0.0291 |  | 1555.7 | -0.0984 | -0.1191 |  |  |  |  |
| 1131.2 | 0.01 | 0.0739 |  | 1544.9 | -0.0384 | -0.1061 |  |  |  |  |
| 1131.4 | 0.14 | 0.0959 |  | 1566.9 | -0.0284 | -0.1101 |  |  |  |  |
| 1139.9 | -0.08 | -0.0761 |  | 1578.5 | -0.1884 | -0.1761 |  |  |  |  |
| 1149.9 | 0.23 | 0.1729 |  | 1578.5 | -0.0984 | -0.1761 |  |  |  |  |
| 1149.9 | 0.23 | 0.1729 |  | 1568.8 | -0.2084 | -0.1121 |  |  |  |  |
| 1147.8 | 0.09 | 0.1219 |  | 1575.9 | -0.0784 | -0.1431 |  |  |  |  |
| 1138.6 | -0.05 | -0.0371 |  | 1575.4 | -0.1984 | -0.1961 |  |  |  |  |
| 1136.1 | 0.07 | 0.0829 |  | 1579.6 | -0.2884 | -0.2511 |  |  |  |  |
| 1163.6 | -0.18 | -0.1121 |  | 1577 | -0.3884 | -0.4301 |  |  |  |  |
| 1169.6 | -0.13 | -0.0851 |  | 1602.4 | -0.2184 | -0.1991 |  |  |  |  |
| 1167.5 | -0.06 | -0.1341 |  | 1602.4 | -0.1284 | -0.1991 |  |  |  |  |
| 1173.2 | -0.01 | 0.0459 |  | 1586.3 | -0.3684 | -0.3251 |  |  |  |  |
| 1173.2 | 0 | 0.0459 |  | 1606 | -0.0184 | -0.0421 |  |  |  |  |
| 1168.9 | -0.09 | -0.0771 |  | 1592.7 | -0.1984 | -0.1501 |  |  |  |  |
| 1165.4 | -0.06 | -0.0851 |  | 1599.8 | -0.1184 | -0.1561 |  |  |  |  |
| 1152.6 | 0.15 | 0.2039 |  | 1599.3 | -0.1884 | -0.2271 |  |  |  |  |
| 1161.7 | -0.02 | -0.0071 |  | 1587.8 | 0.0716 | -0.0171 |  |  |  |  |
| 1157.6 | 0.12 | 0.1329 |  | 1603.5 | -0.0984 | -0.1611 |  |  |  |  |
| 1160.8 | 0.16 | 0.0829 |  | 1614.7 | -0.0484 | -0.1461 |  |  |  |  |
| 1155.3 | 0.14 | 0.1289 |  | 1626.3 | -0.2184 | -0.1381 |  |  |  |  |
| 1171.7 | 0.06 | 0.0379 |  | 1626.3 | -0.1284 | -0.1381 |  |  |  |  |
| 1162.6 | -0.02 | 0.0079 |  | 1629.9 | -0.0184 | -0.0881 |  |  |  |  |
| 1166.5 | -0.14 | -0.1481 |  | 1616.6 | -0.1984 | -0.1531 |  |  |  |  |
| 1170.7 | -0.07 | -0.0211 |  | 1623.8 | -0.1584 | -0.1191 |  |  |  |  |
| 1160 | 0.04 | 0.0939 |  | 1623.3 | -0.1984 | -0.1461 |  |  |  |  |
| 1158.4 | 0.25 | 0.1689 |  | 1627.5 | -0.1184 | -0.1141 |  |  |  |  |
| 1181.9 | -0.02 | -0.0011 |  | 1650.3 | -0.2084 | -0.1271 |  |  |  |  |
| 1193.5 | -0.11 | -0.1921 |  | 1650.3 | -0.1284 | -0.1271 |  |  |  |  |
| 1185.6 | -0.08 | -0.0921 |  | 1653.9 | -0.0084 | -0.0701 |  |  |  |  |
| 1183.9 | 0.05 | -0.0071 |  | 1640.5 | -0.1784 | -0.2011 |  |  |  |  |
| 1183.9 | 0.08 | 0.0169 |  | 1647.7 | -0.1184 | -0.0861 |  |  |  |  |
| 1194.1 | -0.23 | -0.1881 |  | 1652.4 | -0.0684 | -0.0941 |  |  |  |  |
| 1192.1 | -0.26 | -0.2581 |  | 1642.2 | -0.2484 | -0.1531 |  |  |  |  |
| 1205.9 | -0.01 | -0.0361 |  | 1651.4 | -0.1184 | -0.1151 |  |  |  |  |
| 1217.5 | -0.2 | -0.2791 |  | 1674.2 | -0.0884 | -0.0601 |  |  |  |  |
| 1217.7 | -0.26 | -0.2671 |  | 1677.8 | -0.0084 | -0.0211 |  |  |  |  |
| 1217.7 | -0.31 | -0.2671 |  | 1664.5 | -0.1884 | -0.1911 |  |  |  |  |
| 1209.5 | -0.04 | -0.0941 |  | 1670.9 | -0.0084 | -0.0221 |  |  |  |  |
| 1211.7 | -0.07 | -0.1421 |  | 1669.4 | 0.0716 | -0.0271 |  |  |  |  |
| 1216 | -0.27 | -0.2871 |  | 1675.3 | -0.0884 | -0.0701 |  |  |  |  |
| 1226 | -0.23 | -0.1631 |  | 1698.1 | -0.0784 | 0.0059 |  |  |  |  |
| 1229.8 | -0.25 | -0.1761 |  | 1701.7 | -0.0084 | 0.0819 |  |  |  |  |
| 1241.4 | -0.13 | -0.2071 |  | 1688.4 | -0.2084 | -0.1461 |  |  |  |  |
| 1238.8 | -0.16 | -0.1941 |  | 1693.4 | 0.0816 | -0.0041 |  |  |  |  |
| 1238.3 | -0.15 | -0.1891 |  | 1682.1 | 0.0316 | 0.0139 |  |  |  |  |
| 1233.3 | -0.19 | -0.1391 |  | 1690 | -0.2184 | -0.1691 |  |  |  |  |
| 1240 | -0.25 | -0.1881 |  | 1683.6 | 0.0316 | -0.0401 |  |  |  |  |
| 1253.7 | -0.19 | -0.1721 |  | 1710.5 | -0.0484 | -0.0271 |  |  |  |  |
| 1265.3 | -0.13 | -0.1501 |  | 1722.1 | -0.0984 | -0.0031 |  |  |  |  |
| 1267.9 | -0.06 | -0.1411 |  | 1725.7 | 0.0116 | -0.0421 |  |  |  |  |
| 1269.2 | -0.03 | -0.0951 |  | 1712.3 | -0.2084 | -0.1301 |  |  |  |  |
| 1257.4 | -0.01 | -0.0831 |  | 1720.3 | 0.1216 | 0.0459 |  |  |  |  |
| 1262.8 | -0.03 | -0.1001 |  | 1719.5 | -0.0384 | 0.0219 |  |  |  |  |
| 1262.3 | -0.14 | -0.0811 |  | 1717.3 | 0.0516 | 0.0369 |  |  |  |  |
| 1257.2 | -0.17 | -0.1091 |  | 1707.8 | 0.1716 | 0.1079 |  |  |  |  |
| 1293 | 0.04 | 0.0289 |  | 1716.3 | -0.0884 | -0.0501 |  |  |  |  |
| 1292.9 | 0.01 | -0.0001 |  | 1724.2 | 0.0116 | -0.0351 |  |  |  |  |
| 1292.9 | 0.01 | -0.0001 |  | 1706 | 0.0616 | 0.0999 |  |  |  |  |
| 1289.2 | 0.03 | 0.0019 |  | 1714 | -0.2284 | -0.1801 |  |  |  |  |
| 1285.9 | 0.1 | 0.0229 |  | 1707.5 | 0.0616 | 0.1319 |  |  |  |  |
| 1284.5 | 0.06 | 0.0299 |  | 1723.2 | -0.0984 | -0.0351 |  |  |  |  |
| 1283.5 | -0.08 | 0.0049 |  | 1749.6 | 0.0516 | -0.0361 |  |  |  |  |
| 1291.4 | 0.06 | -0.0051 |  | 1746 | -0.0984 | -0.0161 |  |  |  |  |
| 1291.4 | -0.07 | -0.0051 |  | 1749.6 | 0.0316 | -0.0271 |  |  |  |  |
| 1281.1 | -0.17 | -0.1331 |  | 1736.3 | -0.2084 | -0.2651 |  |  |  |  |
| 1290.4 | -0.02 | -0.0271 |  | 1742.7 | 0.0016 | -0.0791 |  |  |  |  |
| 1301.6 | -0.01 | -0.0171 |  | 1738.1 | -0.3684 | -0.2921 |  |  |  |  |
| 1318.1 | -0.04 | -0.0421 |  | 1743.4 | -0.0984 | -0.0561 |  |  |  |  |
| 1316.8 | -0.05 | -0.0481 |  | 1748.2 | -0.0484 | -0.0321 |  |  |  |  |
| 1316.8 | -0.01 | -0.0481 |  | 1748.2 | 0.0216 | -0.0321 |  |  |  |  |
| 1309.9 | 0.11 | 0.1079 |  | 1729.9 | 0.0516 | -0.0081 |  |  |  |  |
| 1309.9 | 0.08 | 0.1079 |  | 1737.9 | -0.2284 | -0.3101 |  |  |  |  |
| 1305.3 | -0.01 | -0.0261 |  | 1731.5 | 0.0616 | 0.0039 |  |  |  |  |
| 1310.7 | 0.03 | 0.0639 |  | 1747.1 | -0.0984 | -0.0351 |  |  |  |  |
| 1308.4 | 0.07 | 0.0929 |  | 1758.3 | -0.0284 | -0.1081 |  |  |  |  |
| 1308.4 | 0.05 | 0.0929 |  | 1762 | -0.0884 | -0.1821 |  |  |  |  |
| 1298.9 | 0.15 | 0.1119 |  | 1773.5 | -0.0184 | -0.0111 |  |  |  |  |
| 1298.9 | 0.18 | 0.1119 |  | 1760.2 | -0.1984 | -0.1921 |  |  |  |  |
| 1314.3 | -0.03 | -0.0611 |  | 1768.2 | 0.1116 | 0.0449 |  |  |  |  |
| 1337.1 | -0.12 | -0.0821 |  | 1766.6 | 0.0316 | -0.0221 |  |  |  |  |
| 1343.1 | -0.03 | -0.0451 |  | 1767.4 | -0.0284 | 0.0129 |  |  |  |  |
| 1340.7 | -0.06 | -0.1321 |  | 1772.1 | -0.0184 | -0.0111 |  |  |  |  |
| 1331.2 | -0.27 | -0.1921 |  | 1753.8 | 0.0416 | 0.0359 |  |  |  |  |
| 1329.2 | -0.27 | -0.2701 |  | 1761.8 | -0.2584 | -0.1851 |  |  |  |  |
| 1334.6 | -0.09 | -0.0741 |  | 1755.4 | 0.0716 | 0.0289 |  |  |  |  |
| 1334.6 | -0.04 | -0.0741 |  | 1782.3 | -0.0184 | -0.0131 |  |  |  |  |
| 1339.3 | -0.07 | -0.1281 |  | 1793.9 | -0.0884 | -0.0781 |  |  |  |  |
| 1334 | -0.15 | -0.0911 |  | 1797.5 | -0.0384 | -0.1291 |  |  |  |  |
| 1364.7 | 0 | 0.0459 |  | 1784.1 | -0.2084 | -0.1521 |  |  |  |  |
| 1364.7 | -0.01 | 0.0459 |  | 1776.9 | 0.1316 | 0.0649 |  |  |  |  |
| 1358.5 | -0.05 | 0.0019 |  | 1791.3 | -0.1284 | -0.1651 |  |  |  |  |
| 1366.1 | 0.12 | 0.0679 |  | 1796 | -0.0384 | -0.0831 |  |  |  |  |
| 1366.1 | 0.02 | 0.0679 |  | 1796 | -0.0184 | -0.0831 |  |  |  |  |
| 1363.2 | 0.06 | 0.0409 |  | 1790.8 | -0.1884 | -0.1751 |  |  |  |  |
| 1344.9 | 0.1 | 0.0309 |  | 1785.7 | -0.2084 | -0.2401 |  |  |  |  |
| 1349.9 | -0.14 | -0.1851 |  | 1779.3 | 0.0716 | 0.0849 |  |  |  |  |
| 1373.4 | -0.01 | 0.0099 |  | 1795 | -0.0884 | -0.0831 |  |  |  |  |
| 1379 | -0.28 | -0.2361 |  | 1806.2 | -0.0184 | -0.0851 |  |  |  |  |
| 1391.1 | -0.04 | -0.0221 |  | 1817.8 | -0.1984 | -0.1181 |  |  |  |  |
| 1388.6 | 0 | -0.0701 |  | 1817.8 | -0.0784 | -0.1181 |  |  |  |  |
| 1388.6 | -0.01 | -0.0701 |  | 1812.2 | -0.3984 | -0.3601 |  |  |  |  |
| 1379 | -0.26 | -0.2401 |  | 1821.4 | -0.0084 | -0.1001 |  |  |  |  |
| 1384.9 | 0.08 | 0.0319 |  | 1808 | -0.2084 | -0.2161 |  |  |  |  |
| 1391.3 | -0.08 | -0.0391 |  | 1811.9 | -0.3984 | -0.3631 |  |  |  |  |
| 1377 | -0.29 | -0.2191 |  | 1803.7 | -0.0284 | -0.0321 |  |  |  |  |
| 1382.4 | -0.04 | -0.0651 |  | 1819.9 | -0.0584 | -0.1061 |  |  |  |  |
| 1370.7 | 0.2 | 0.1269 |  | 1819.9 | -0.0284 | -0.1061 |  |  |  |  |
| 1379.3 | -0.15 | -0.2231 |  | 1813.6 | -0.3284 | -0.3391 |  |  |  |  |
| 1386.1 | -0.06 | -0.0161 |  | 1814.7 | -0.1984 | -0.2681 |  |  |  |  |
| 1397.3 | -0.01 | -0.0101 |  | 1818.9 | -0.0884 | -0.1151 |  |  |  |  |
| 1412.5 | 0.03 | 0.0239 |  | 1816.3 | -0.2784 | -0.1861 |  |  |  |  |
| 1412.5 | 0 | 0.0239 |  | 1806.7 | -0.1884 | -0.1211 |  |  |  |  |
| 1408.9 | -0.01 | 0.0399 |  | 1830.1 | -0.0684 | 0.0159 |  |  |  |  |
| 1406.4 | -0.02 | -0.0571 |  | 1841.7 | -0.1084 | -0.0281 |  |  |  |  |
| 1394.6 | 0.13 | 0.0529 |  | 1845.3 | 0.0116 | 0.0449 |  |  |  |  |
| 1411.1 | 0.08 | 0.0119 |  | 1841.1 | -0.1384 | -0.0401 |  |  |  |  |
| 1405.8 | -0.14 | -0.1061 |  | 1839.2 | -0.1684 | -0.1511 |  |  |  |  |
| 1400.8 | -0.13 | -0.1821 |  | 1843.9 | -0.0184 | 0.0529 |  |  |  |  |
| 1410 | -0.05 | 0.0299 |  | 1838.7 | -0.2084 | -0.1901 |  |  |  |  |
| 1436.5 | -0.06 | -0.0401 |  | 1827.2 | 0.0616 | 0.0229 |  |  |  |  |
| 1421.2 | -0.01 | 0.0539 |  | 1842.8 | -0.0684 | -0.0001 |  |  |  |  |
| 1432.8 | -0.14 | -0.0711 |  | 1869.3 | 0.0616 | -0.0041 |  |  |  |  |
| 1439 | 0.04 | -0.0131 |  | 1865.7 | -0.0684 | -0.0421 |  |  |  |  |
| 1436.5 | 0 | -0.0291 |  | 1862.4 | -0.0984 | -0.1271 |  |  |  |  |
| 1436.5 | 0 | -0.0291 |  | 1869.3 | -0.0084 | 0.0039 |  |  |  |  |
| 1432.8 | 0.03 | -0.0541 |  | 1848.7 | 0.1616 | 0.0739 |  |  |  |  |
| 1430.3 | -0.09 | -0.1601 |  | 1856.7 | 0.0816 | 0.0969 |  |  |  |  |
| 1435 | 0.04 | -0.0251 |  | 1863.1 | -0.1184 | -0.1221 |  |  |  |  |
| 1429.8 | -0.13 | -0.1741 |  | 1851.4 | 0.1716 | 0.1529 |  |  |  |  |
| 1424.7 | -0.15 | -0.1381 |  | 1851.4 | 0.2016 | 0.1529 |  |  |  |  |
| 1434 | -0.05 | -0.0171 |  | 1867.8 | -0.0284 | 0.0199 |  |  |  |  |
| 1431.4 | -0.19 | -0.1101 |  | 1858.6 | -0.0784 | -0.0461 |  |  |  |  |
| 1447.2 | -0.02 | -0.0261 |  | 1854.3 | 0.2416 | 0.1659 |  |  |  |  |
| 1441 | -0.02 | 0.0149 |  | 1862.6 | -0.1884 | -0.1311 |  |  |  |  |
| 1441 | -0.06 | 0.0149 |  | 1851.1 | 0.0716 | 0.1469 |  |  |  |  |
| 1462.4 | 0 | 0.0369 |  | 1878 | -0.0584 | -0.0051 |  |  |  |  |
| 1462.4 | 0.02 | 0.0369 |  | 1889.6 | -0.1184 | -0.0981 |  |  |  |  |
| 1441.2 | 0.04 | -0.0091 |  | 1893.2 | -0.0084 | -0.0141 |  |  |  |  |
| 1456.2 | -0.05 | -0.0481 |  | 1888.9 | -0.1484 | -0.1011 |  |  |  |  |
| 1453.1 | -0.16 | -0.1901 |  | 1887 | -0.1384 | -0.2051 |  |  |  |  |
| 1444.6 | 0.05 | 0.0449 |  | 1877.6 | 0.0216 | 0.0109 |  |  |  |  |
| 1460.9 | 0.09 | 0.0419 |  | 1882.6 | -0.0484 | -0.0961 |  |  |  |  |
| 1442.8 | 0.01 | 0.0399 |  | 1886.5 | -0.1884 | -0.1781 |  |  |  |  |
| 1455.8 | -0.14 | -0.0571 |  | 1890.7 | -0.1084 | -0.0541 |  |  |  |  |
| 1450.7 | -0.19 | -0.1741 |  | 1901.9 | -0.0484 | -0.1461 |  |  |  |  |
| 1482.7 | 0.02 | 0.0909 |  | 1913.5 | -0.1584 | -0.0791 |  |  |  |  |
| 1485.8 | 0.2 | 0.1229 |  | 1913.5 | -0.0784 | -0.0791 |  |  |  |  |
| 1465.2 | 0.03 | 0.0319 |  | 1912.6 | -0.0984 | -0.1131 |  |  |  |  |
| 1465.8 | 0.08 | 0.0309 |  | 1917.1 | -0.0184 | 0.0034 |  |  |  |  |
| 1473.8 | 0.1 | 0.0499 |  | 1911 | -0.0684 | -0.1426 |  |  |  |  |
| 1474.8 | -0.02 | -0.0031 |  | 1903.9 | 0.0516 | -0.0381 |  |  |  |  |
| 1480.2 | -0.1 | -0.0461 |  | 1915.7 | -0.0584 | -0.0341 |  |  |  |  |
| 1480.2 | -0.05 | -0.0461 |  | 1915.7 | 0.0016 | -0.0341 |  |  |  |  |
| 1465.4 | 0.08 | 0.0619 |  | 1910.5 | -0.1884 | -0.1496 |  |  |  |  |
| 1468.5 | 0.13 | 0.1119 |  | 1914.7 | -0.0784 | -0.0576 |  |  |  |  |
| 1468.5 | 0.15 | 0.1059 |  | 1925.9 | -0.0284 | 0.0419 |  |  |  |  |
| 1470.7 | 0.12 | 0.1099 |  | 1937.5 | -0.1884 | -0.1456 |  |  |  |  |
| 1484.9 | 0.09 | 0.1129 |  | 1937.5 | -0.0784 | -0.1456 |  |  |  |  |
| 1466.7 | 0.05 | 0.0729 |  | 1939.4 | -0.1684 | -0.0946 |  |  |  |  |
| 1466.7 | 0 | 0.0729 |  | 1941.1 | -0.0184 | -0.0156 |  |  |  |  |
| 1473.1 | 0.05 | 0.0209 |  | 1939.6 | -0.0084 | -0.0871 |  |  |  |  |
| 1506.7 | -0.14 | -0.0821 |  | 1934.4 | -0.1984 | -0.1681 |  |  |  |  |
| 1510.3 | 0 | -0.0411 |  | 1922.9 | 0.0716 | 0.0329 |  |  |  |  |
| 1510.3 | 0 | -0.0411 |  | 1938.6 | -0.0684 | -0.1136 |  |  |  |  |
| 1506 | -0.1 | -0.0721 |  | 1965 | 0.0516 | -0.0086 |  |  |  |  |
| 1506.6 | 0.02 | -0.0401 |  | 1949.8 | -0.0284 | -0.0026 |  |  |  |  |
| 1489.1 | 0.05 | 0.0799 |  | 1961.4 | -0.1884 | -0.1406 |  |  |  |  |
| 1489.7 | 0.1 | 0.0869 |  | 1961.4 | -0.1184 | -0.1406 |  |  |  |  |
| 1498.8 | -0.01 | -0.0771 |  | 1965 | 0.0116 | -0.0026 |  |  |  |  |
| 1504.1 | -0.11 | -0.1291 |  | 1958.1 | 0.0016 | -0.0981 |  |  |  |  |
| 1504.1 | -0.08 | -0.1291 |  | 1958.8 | -0.1884 | -0.1491 |  |  |  |  |
| 1492.4 | 0.1 | 0.1429 |  | 1956 | 0.0216 | -0.0431 |  |  |  |  |
| 1492.4 | 0.19 | 0.1249 |  | 1946.8 | 0.0616 | -0.0236 |  |  |  |  |
| 1501 | -0.14 | -0.1331 |  | 1962.5 | -0.0684 | -0.1121 |  |  |  |  |
| 1496.4 | 0.06 | 0.0729 |  | 1973.7 | -0.0284 | -0.0311 |  |  |  |  |
| 1508.8 | 0.02 | 0.0089 |  | 1985.3 | -0.1784 | -0.1031 |  |  |  |  |
| 1508.8 | -0.01 | 0.0089 |  | 1985.3 | -0.1184 | -0.1031 |  |  |  |  |
| 1490.6 | 0.04 | 0.1029 |  | 1988.9 | 0.0016 | -0.0351 |  |  |  |  |
| 1503.6 | -0.19 | -0.1211 |  | 1982 | -0.0084 | -0.0441 |  |  |  |  |
| 1503.6 | -0.15 | -0.1221 |  | 1982.8 | -0.0684 | -0.0566 |  |  |  |  |
| 1507.8 | -0.06 | -0.0491 |  | 1980.6 | 0.0416 | 0.0004 |  |  |  |  |
| 1519 | -0.01 | -0.0411 |  | 1969.2 | 0.0416 | 0.0389 |  |  |  |  |
| 1530.6 | -0.14 | -0.1271 |  | 1970.8 | 0.0616 | -0.0211 |  |  |  |  |
| 1512.8 | -0.02 | -0.0121 |  | 1986.4 | -0.0784 | -0.1016 |  |  |  |  |
| 1530 | -0.1 | -0.1491 |  | 1997.7 | -0.0584 | -0.0421 |  |  |  |  |
| 1522.7 | -0.01 | -0.0931 |  | 2009.3 | -0.1084 | -0.0506 |  |  |  |  |
| 1514.6 | 0.05 | -0.0271 |  | 2012.9 | 0.0116 | -0.0086 |  |  |  |  |
| 1527.5 | -0.15 | -0.1881 |  | 2008.6 | -0.1384 | -0.0391 |  |  |  |  |
| 1522.5 | -0.15 | -0.1401 |  | 2005.9 | 0.0116 | 0.0209 |  |  |  |  |
| 1531.7 | -0.07 | -0.1011 |  | 2004.5 | 0.0616 | 0.0459 |  |  |  |  |
| 1543 | -0.01 | -0.0781 |  | 2003.5 | 0.1216 | 0.0479 |  |  |  |  |
| 1554.5 | -0.13 | -0.1381 |  | 2011.4 | 0.0116 | -0.0626 |  |  |  |  |
| 1558.1 | 0 | -0.0421 |  | 1994.7 | 0.0716 | -0.0056 |  |  |  |  |
| 1558.1 | -0.01 | -0.0421 |  | 2010.4 | -0.0384 | -0.0691 |  |  |  |  |
| 1546.6 | -0.04 | -0.0681 |  | 2021.6 | -0.0184 | 0.0099 |  |  |  |  |
| 1552 | -0.13 | -0.1471 |  | 2033.2 | -0.0884 | 0.0079 |  |  |  |  |
| 1552 | -0.07 | -0.1481 |  | 2036.8 | -0.0084 | 0.0069 |  |  |  |  |
| 1551.5 | -0.2 | -0.1701 |  | 2029.9 | 0.0916 | 0.0754 |  |  |  |  |
| 1551.5 | -0.15 | -0.1701 |  | 2029.9 | 0.0116 | 0.0754 |  |  |  |  |
| 1546.4 | -0.14 | -0.0701 |  | 2030.7 | 0.0316 | 0.0944 |  |  |  |  |
| 1555.7 | -0.07 | -0.1191 |  | 2028.4 | 0.0416 | 0.0849 |  |  |  |  |
| 1578.5 | -0.14 | -0.1761 |  | 2035.3 | -0.0184 | -0.0261 |  |  |  |  |
| 1561.3 | 0.04 | -0.0311 |  | 2017.1 | 0.0716 | 0.1104 |  |  |  |  |
| 1575.9 | -0.11 | -0.1431 |  | 2018.6 | 0.0816 | 0.0744 |  |  |  |  |
| 1575.4 | -0.14 | -0.1961 |  | 2034.3 | -0.0484 | -0.0001 |  |  |  |  |
| 1570.4 | -0.21 | -0.1551 |  | 2045.5 | -0.0284 | -0.0581 |  |  |  |  |
| 1579.1 | -0.22 | -0.1581 |  | 2047.3 | -0.2084 | -0.1291 |  |  |  |  |
| 1602.4 | -0.15 | -0.1991 |  | 2055.3 | 0.1516 | 0.0539 |  |  |  |  |
| 1586.3 | -0.28 | -0.3251 |  | 2060.1 | 0.1416 | 0.1189 |  |  |  |  |
| 1606 | 0 | -0.0421 |  | 2053.8 | 0.0816 | 0.0079 |  |  |  |  |
| 1606 | 0 | -0.0421 |  | 2053.8 | -0.0084 | 0.0079 |  |  |  |  |
| 1599.8 | -0.09 | -0.1561 |  | 2049 | -0.2384 | -0.1931 |  |  |  |  |
| 1599.3 | -0.14 | -0.2061 |  | 2042.6 | 0.0716 | 0.0759 |  |  |  |  |
| 1626.3 | -0.14 | -0.1381 |  | 2084.7 | -0.0184 | 0.0239 |  |  |  |  |
| 1623.8 | -0.1 | -0.1191 |  | 2079.3 | 0.1316 | 0.0849 |  |  |  |  |
| 1623.3 | -0.14 | -0.1351 |  | 2077.7 | -0.0284 | -0.0271 |  |  |  |  |
| 1618.2 | -0.17 | -0.1271 |  | 2086.1 | 0.0116 | 0.0649 |  |  |  |  |
| 1627.5 | -0.07 | -0.1141 |  | 2076.3 | 0.0216 | -0.0101 |  |  |  |  |
| 1627 | -0.17 | -0.1401 |  | 2066.7 | 0.2116 | 0.2199 |  |  |  |  |
| 1650.3 | -0.14 | -0.1271 |  | 2066.7 | 0.3016 | 0.2199 |  |  |  |  |
| 1653.9 | 0.01 | -0.0701 |  | 2083.2 | 0.0016 | 0.0859 |  |  |  |  |
| 1653.9 | 0 | -0.0701 |  | 2069.7 | 0.2416 | 0.1429 |  |  |  |  |
| 1647.7 | -0.08 | -0.0861 |  | 2093.4 | -0.0284 | 0.0459 |  |  |  |  |
| 1651.7 | -0.06 | -0.1111 |  | 2105 | -0.0884 | -0.0301 |  |  |  |  |
| 1652.4 | -0.04 | -0.0941 |  | 2108.6 | -0.0284 | -0.0521 |  |  |  |  |
| 1647.2 | -0.14 | -0.0821 |  | 2090.7 | 0.2216 | 0.1319 |  |  |  |  |
| 1642.2 | -0.18 | -0.1531 |  | 2093.8 | 0.0516 | 0.0369 |  |  |  |  |
| 1651.4 | -0.08 | -0.1151 |  | 2099.3 | -0.1884 | -0.2251 |  |  |  |  |
| 1661.5 | -0.27 | -0.1891 |  | 2101.9 | -0.1984 | -0.1311 |  |  |  |  |
| 1677.8 | 0.02 | -0.0211 |  | 2096.9 | -0.1684 | -0.1821 |  |  |  |  |
| 1677.8 | 0.01 | -0.0211 |  | 2090.4 | 0.0816 | 0.1619 |  |  |  |  |
| 1671.6 | -0.08 | -0.0181 |  | 2106.1 | -0.0884 | -0.0181 |  |  |  |  |
| 1669.4 | 0.03 | -0.0271 |  | 2117.3 | -0.0184 | -0.0341 |  |  |  |  |
| 1668.5 | -0.12 | -0.0431 |  | 2128.9 | -0.0884 | -0.0341 |  |  |  |  |
| 1666.1 | -0.19 | -0.1441 |  | 2132.5 | -0.0184 | -0.0561 |  |  |  |  |
| 1675.3 | -0.05 | -0.0701 |  | 2119.1 | -0.2084 | -0.1241 |  |  |  |  |
| 1701.8 | -0.02 | 0.0599 |  | 2131.1 | 0.0116 | -0.0631 |  |  |  |  |
| 1686.5 | -0.17 | -0.1561 |  | 2125.8 | -0.1984 | -0.2201 |  |  |  |  |
| 1701.7 | 0 | 0.0819 |  | 2120.8 | -0.1484 | -0.2211 |  |  |  |  |
| 1692.2 | -0.11 | -0.0351 |  | 2114.3 | 0.0816 | 0.0199 |  |  |  |  |
| 1680.5 | 0.03 | 0.0219 |  | 2130 | -0.0784 | -0.0551 |  |  |  |  |
| 1681.1 | 0.09 | 0.0329 |  | 2156.5 | 0.0616 | 0.0329 |  |  |  |  |
| 1693.4 | 0.06 | -0.0041 |  | 2141.2 | -0.0384 | -0.0001 |  |  |  |  |
| 1700.3 | 0.07 | 0.0209 |  | 2152.8 | -0.1184 | -0.0651 |  |  |  |  |
| 1682.1 | 0.03 | 0.0139 |  | 2156.5 | -0.0084 | 0.0269 |  |  |  |  |
| 1690 | -0.16 | -0.1691 |  | 2150.3 | -0.0884 | -0.1781 |  |  |  |  |
| 1725.7 | -0.06 | -0.0021 |  | 2155 | 0.0516 | 0.0339 |  |  |  |  |
| 1710.5 | -0.02 | -0.0271 |  | 2149.8 | -0.1984 | -0.1901 |  |  |  |  |
| 1725.7 | 0.02 | -0.0421 |  |  |  |  |  |  |  |  |
| 1725.7 | 0.02 | -0.0421 |  |  |  |  |  |  |  |  |
| 1705.1 | 0.13 | 0.1919 |  |  |  |  |  |  |  |  |
| 1719.5 | -0.02 | 0.0219 |  |  |  |  |  |  |  |  |
| 1717.3 | 0.05 | 0.0369 |  |  |  |  |  |  |  |  |
| 1717.3 | 0.07 | 0.0369 |  |  |  |  |  |  |  |  |
| 1707.8 | 0.12 | 0.1079 |  |  |  |  |  |  |  |  |
| 1707.8 | 0.14 | 0.1079 |  |  |  |  |  |  |  |  |
| 1706 | 0.06 | 0.0999 |  |  |  |  |  |  |  |  |
| 1714 | -0.16 | -0.1801 |  |  |  |  |  |  |  |  |
| 1723.2 | -0.07 | -0.0351 |  |  |  |  |  |  |  |  |
| 1736.9 | -0.28 | -0.2801 |  |  |  |  |  |  |  |  |
| 1736.9 | -0.3 | -0.2801 |  |  |  |  |  |  |  |  |
| 1749.6 | 0.05 | -0.0271 |  |  |  |  |  |  |  |  |
| 1749.6 | 0.03 | -0.0271 |  |  |  |  |  |  |  |  |
| 1745.3 | -0.1 | -0.0321 |  |  |  |  |  |  |  |  |
| 1729.9 | 0 | 0.0459 |  |  |  |  |  |  |  |  |
| 1729 | 0.06 | 0.0399 |  |  |  |  |  |  |  |  |
| 1738.1 | -0.28 | -0.2921 |  |  |  |  |  |  |  |  |
| 1743.4 | -0.06 | -0.0561 |  |  |  |  |  |  |  |  |
| 1748.2 | -0.05 | -0.0321 |  |  |  |  |  |  |  |  |
| 1729.9 | 0.04 | -0.0081 |  |  |  |  |  |  |  |  |
| 1747.1 | -0.06 | -0.0391 |  |  |  |  |  |  |  |  |
| 1758.3 | -0.17 | -0.1081 |  |  |  |  |  |  |  |  |
| 1773.5 | 0.03 | -0.0111 |  |  |  |  |  |  |  |  |
| 1773.5 | -0.01 | -0.0111 |  |  |  |  |  |  |  |  |
| 1752.9 | 0.08 | 0.0169 |  |  |  |  |  |  |  |  |
| 1767.4 | 0 | 0.0129 |  |  |  |  |  |  |  |  |
| 1772.1 | 0.04 | -0.0111 |  |  |  |  |  |  |  |  |
| 1772.1 | -0.02 | -0.0111 |  |  |  |  |  |  |  |  |
| 1753.8 | 0.06 | 0.0359 |  |  |  |  |  |  |  |  |
| 1761.8 | -0.19 | -0.1851 |  |  |  |  |  |  |  |  |
| 1782.3 | 0 | -0.0131 |  |  |  |  |  |  |  |  |
| 1793.9 | -0.14 | -0.0781 |  |  |  |  |  |  |  |  |
| 1787.1 | -0.3 | -0.2871 |  |  |  |  |  |  |  |  |
| 1781.6 | 0.04 | -0.0011 |  |  |  |  |  |  |  |  |
| 1776.9 | 0.12 | 0.0649 |  |  |  |  |  |  |  |  |
| 1791.3 | -0.09 | -0.1651 |  |  |  |  |  |  |  |  |
| 1791.3 | -0.09 | -0.1651 |  |  |  |  |  |  |  |  |
| 1796 | -0.02 | -0.0831 |  |  |  |  |  |  |  |  |
| 1777.8 | 0.06 | 0.0549 |  |  |  |  |  |  |  |  |
| 1790.8 | -0.13 | -0.1751 |  |  |  |  |  |  |  |  |
| 1795 | -0.06 | -0.0831 |  |  |  |  |  |  |  |  |
| 1817.8 | -0.13 | -0.1181 |  |  |  |  |  |  |  |  |
| 1812.2 | -0.3 | -0.3601 |  |  |  |  |  |  |  |  |
| 1811.9 | -0.3 | -0.3631 |  |  |  |  |  |  |  |  |
| 1803.7 | -0.06 | -0.0321 |  |  |  |  |  |  |  |  |
| 1819.9 | -0.04 | -0.1061 |  |  |  |  |  |  |  |  |
| 1818.9 | -0.05 | -0.1151 |  |  |  |  |  |  |  |  |
| 1818.4 | -0.17 | -0.1001 |  |  |  |  |  |  |  |  |
| 1816.3 | -0.21 | -0.1861 |  |  |  |  |  |  |  |  |
| 1806.7 | -0.11 | -0.1211 |  |  |  |  |  |  |  |  |
| 1846.6 | 0.09 | 0.0549 |  |  |  |  |  |  |  |  |
| 1830.1 | -0.04 | 0.0159 |  |  |  |  |  |  |  |  |
| 1837.3 | -0.29 | -0.2141 |  |  |  |  |  |  |  |  |
| 1845.3 | 0.04 | 0.0449 |  |  |  |  |  |  |  |  |
| 1845.3 | 0.01 | 0.0449 |  |  |  |  |  |  |  |  |
| 1841.7 | 0.01 | -0.0271 |  |  |  |  |  |  |  |  |
| 1839.2 | -0.1 | -0.1501 |  |  |  |  |  |  |  |  |
| 1839.2 | -0.1 | -0.1511 |  |  |  |  |  |  |  |  |
| 1836 | -0.11 | -0.1851 |  |  |  |  |  |  |  |  |
| 1843.9 | 0 | 0.0529 |  |  |  |  |  |  |  |  |
| 1838.7 | -0.15 | -0.1901 |  |  |  |  |  |  |  |  |
| 1833.6 | -0.19 | -0.1541 |  |  |  |  |  |  |  |  |
| 1842.8 | -0.05 | -0.0001 |  |  |  |  |  |  |  |  |
| 1865.7 | -0.11 | -0.0421 |  |  |  |  |  |  |  |  |
| 1862.4 | -0.07 | -0.1271 |  |  |  |  |  |  |  |  |
| 1869.3 | 0.02 | 0.0039 |  |  |  |  |  |  |  |  |
| 1869.3 | 0 | 0.0039 |  |  |  |  |  |  |  |  |
| 1848.7 | 0.07 | 0.0739 |  |  |  |  |  |  |  |  |
| 1848.7 | 0.14 | 0.0739 |  |  |  |  |  |  |  |  |
| 1856.7 | 0.1 | 0.0969 |  |  |  |  |  |  |  |  |
| 1863.1 | -0.09 | -0.1221 |  |  |  |  |  |  |  |  |
| 1851.4 | 0.14 | 0.1529 |  |  |  |  |  |  |  |  |
| 1859.9 | -0.08 | -0.1111 |  |  |  |  |  |  |  |  |
| 1867.8 | 0.09 | 0.0199 |  |  |  |  |  |  |  |  |
| 1858.6 | -0.05 | -0.0461 |  |  |  |  |  |  |  |  |
| 1862.6 | -0.14 | -0.1311 |  |  |  |  |  |  |  |  |
| 1866.8 | -0.09 | -0.0051 |  |  |  |  |  |  |  |  |
| 1856 | 0.07 | 0.1189 |  |  |  |  |  |  |  |  |
| 1854.5 | 0.26 | 0.1869 |  |  |  |  |  |  |  |  |
| 1878 | -0.03 | -0.0051 |  |  |  |  |  |  |  |  |
| 1883.6 | -0.18 | -0.1341 |  |  |  |  |  |  |  |  |
| 1889.6 | -0.13 | -0.0981 |  |  |  |  |  |  |  |  |
| 1893.2 | -0.01 | -0.0141 |  |  |  |  |  |  |  |  |
| 1893.2 | 0 | -0.0141 |  |  |  |  |  |  |  |  |
| 1888.9 | -0.09 | -0.1011 |  |  |  |  |  |  |  |  |
| 1881.7 | -0.02 | -0.0721 |  |  |  |  |  |  |  |  |
| 1877.6 | 0.03 | 0.0109 |  |  |  |  |  |  |  |  |
| 1882.6 | -0.02 | -0.0961 |  |  |  |  |  |  |  |  |
| 1886.5 | -0.14 | -0.1781 |  |  |  |  |  |  |  |  |
| 1890.7 | -0.07 | -0.0541 |  |  |  |  |  |  |  |  |
| 1880 | 0.04 | -0.0271 |  |  |  |  |  |  |  |  |
| 1898.1 | -0.19 | -0.1071 |  |  |  |  |  |  |  |  |
| 1901.9 | -0.15 | -0.1461 |  |  |  |  |  |  |  |  |
| 1913.5 | -0.11 | -0.0791 |  |  |  |  |  |  |  |  |
| 1912.6 | -0.03 | -0.1131 |  |  |  |  |  |  |  |  |
| 1912.6 | -0.07 | -0.1131 |  |  |  |  |  |  |  |  |
| 1917.1 | 0.03 | 0.0034 |  |  |  |  |  |  |  |  |
| 1917.1 | 0.01 | 0.0034 |  |  |  |  |  |  |  |  |
| 1905.6 | -0.08 | -0.0221 |  |  |  |  |  |  |  |  |
| 1915.7 | 0.02 | -0.0341 |  |  |  |  |  |  |  |  |
| 1915.7 | -0.03 | -0.0341 |  |  |  |  |  |  |  |  |
| 1910.5 | -0.13 | -0.1496 |  |  |  |  |  |  |  |  |
| 1914.7 | -0.06 | -0.0576 |  |  |  |  |  |  |  |  |
| 1925.9 | -0.01 | 0.0419 |  |  |  |  |  |  |  |  |
| 1937.5 | -0.2 | -0.1456 |  |  |  |  |  |  |  |  |
| 1937.5 | -0.13 | -0.1456 |  |  |  |  |  |  |  |  |
| 1941.1 | 0 | -0.0156 |  |  |  |  |  |  |  |  |
| 1941.1 | 0.01 | -0.0156 |  |  |  |  |  |  |  |  |
| 1920.7 | 0.05 | 0.0239 |  |  |  |  |  |  |  |  |
| 1929.5 | -0.04 | -0.0446 |  |  |  |  |  |  |  |  |
| 1931.7 | -0.07 | -0.0856 |  |  |  |  |  |  |  |  |
| 1934.4 | -0.18 | -0.1776 |  |  |  |  |  |  |  |  |
| 1934.4 | -0.14 | -0.1726 |  |  |  |  |  |  |  |  |
| 1938.6 | -0.04 | -0.1136 |  |  |  |  |  |  |  |  |
| 1949.8 | -0.01 | -0.0026 |  |  |  |  |  |  |  |  |
| 1961.4 | -0.13 | -0.1406 |  |  |  |  |  |  |  |  |
| 1965 | 0.05 | -0.0026 |  |  |  |  |  |  |  |  |
| 1965 | 0.01 | -0.0026 |  |  |  |  |  |  |  |  |
| 1953.5 | -0.02 | 0.0134 |  |  |  |  |  |  |  |  |
| 1958.8 | -0.16 | -0.1491 |  |  |  |  |  |  |  |  |
| 1958.8 | -0.1 | -0.1491 |  |  |  |  |  |  |  |  |
| 1956 | 0.01 | -0.0431 |  |  |  |  |  |  |  |  |
| 1958.3 | -0.15 | -0.0866 |  |  |  |  |  |  |  |  |
| 1962.5 | -0.05 | -0.1146 |  |  |  |  |  |  |  |  |
| 1951.8 | 0.07 | 0.0089 |  |  |  |  |  |  |  |  |
| 1988.9 | -0.03 | -0.0351 |  |  |  |  |  |  |  |  |
| 1973.7 | 0 | -0.0311 |  |  |  |  |  |  |  |  |
| 1985.3 | -0.13 | -0.1031 |  |  |  |  |  |  |  |  |
| 1987.9 | -0.06 | -0.0756 |  |  |  |  |  |  |  |  |
| 1988.9 | 0.03 | -0.0351 |  |  |  |  |  |  |  |  |
| 1988.9 | 0 | -0.0351 |  |  |  |  |  |  |  |  |
| 1989.2 | -0.03 | -0.0211 |  |  |  |  |  |  |  |  |
| 1977.4 | -0.01 | 0.0334 |  |  |  |  |  |  |  |  |
| 1982.8 | -0.03 | -0.0566 |  |  |  |  |  |  |  |  |
| 1980.6 | 0.08 | 0.0004 |  |  |  |  |  |  |  |  |
| 1980.6 | 0.07 | 0.0004 |  |  |  |  |  |  |  |  |
| 1969.2 | 0.05 | 0.0389 |  |  |  |  |  |  |  |  |
| 1986.4 | -0.04 | -0.1016 |  |  |  |  |  |  |  |  |
| 1975.7 | 0.05 | -0.0116 |  |  |  |  |  |  |  |  |
| 1997.7 | -0.03 | -0.0421 |  |  |  |  |  |  |  |  |
| 2009.3 | -0.11 | -0.0506 |  |  |  |  |  |  |  |  |
| 2013 | 0.04 | -0.0001 |  |  |  |  |  |  |  |  |
| 2012.9 | 0.01 | -0.0086 |  |  |  |  |  |  |  |  |
| 2012.9 | 0.01 | -0.0086 |  |  |  |  |  |  |  |  |
| 2009.2 | 0.03 | -0.0366 |  |  |  |  |  |  |  |  |
| 2005.9 | 0.1 | 0.0209 |  |  |  |  |  |  |  |  |
| 2001.3 | 0.01 | -0.0116 |  |  |  |  |  |  |  |  |
| 2004.5 | 0.06 | 0.0459 |  |  |  |  |  |  |  |  |
| 2011.4 | -0.07 | -0.0626 |  |  |  |  |  |  |  |  |
| 2010.4 | -0.02 | -0.0691 |  |  |  |  |  |  |  |  |
| 2021.6 | -0.01 | 0.0099 |  |  |  |  |  |  |  |  |
| 2038.1 | 0.05 | 0.0329 |  |  |  |  |  |  |  |  |
| 2038.1 | -0.04 | 0.0329 |  |  |  |  |  |  |  |  |
| 2036.8 | -0.05 | 0.0069 |  |  |  |  |  |  |  |  |
| 2036.8 | -0.01 | 0.0069 |  |  |  |  |  |  |  |  |
| 2029.9 | 0.11 | 0.0754 |  |  |  |  |  |  |  |  |
| 2029.9 | 0.08 | 0.0754 |  |  |  |  |  |  |  |  |
| 2025.3 | -0.01 | 0.0064 |  |  |  |  |  |  |  |  |
| 2030.7 | 0.03 | 0.0944 |  |  |  |  |  |  |  |  |
| 2028.4 | 0.07 | 0.0849 |  |  |  |  |  |  |  |  |
| 2028.4 | 0.05 | 0.0849 |  |  |  |  |  |  |  |  |
| 2018.9 | 0.15 | 0.0674 |  |  |  |  |  |  |  |  |
| 2038.5 | 0.12 | 0.0619 |  |  |  |  |  |  |  |  |
| 2034.3 | -0.03 | -0.0001 |  |  |  |  |  |  |  |  |
| 2045.5 | -0.01 | -0.0581 |  |  |  |  |  |  |  |  |
| 2060.7 | -0.02 | 0.0619 |  |  |  |  |  |  |  |  |
| 2060.1 | 0.12 | 0.1189 |  |  |  |  |  |  |  |  |
| 2053.8 | 0.08 | 0.0079 |  |  |  |  |  |  |  |  |
| 2049.2 | -0.27 | -0.1861 |  |  |  |  |  |  |  |  |
| 2042.8 | 0.15 | 0.0749 |  |  |  |  |  |  |  |  |
| 2059.3 | 0.05 | 0.0809 |  |  |  |  |  |  |  |  |
| 2049 | -0.17 | -0.1931 |  |  |  |  |  |  |  |  |
| 2084.7 | 0 | 0.0239 |  |  |  |  |  |  |  |  |
| 2084.7 | -0.01 | 0.0239 |  |  |  |  |  |  |  |  |
| 2081 | 0 | 0.0809 |  |  |  |  |  |  |  |  |
| 2086.1 | 0.12 | 0.0899 |  |  |  |  |  |  |  |  |
| 2086.1 | 0.02 | 0.0649 |  |  |  |  |  |  |  |  |
| 2076.3 | 0.07 | -0.0101 |  |  |  |  |  |  |  |  |
| 2076.3 | 0.06 | -0.0101 |  |  |  |  |  |  |  |  |
| 2066.7 | 0.18 | 0.2199 |  |  |  |  |  |  |  |  |
| 2083.2 | 0.06 | 0.0859 |  |  |  |  |  |  |  |  |
| 2069.7 | 0.2 | 0.1429 |  |  |  |  |  |  |  |  |
| 2071.4 | 0.08 | 0.0379 |  |  |  |  |  |  |  |  |
| 2093.4 | -0.01 | 0.0459 |  |  |  |  |  |  |  |  |
| 2099 | -0.28 | -0.2501 |  |  |  |  |  |  |  |  |
| 2111.1 | -0.04 | -0.0311 |  |  |  |  |  |  |  |  |
| 2108.6 | 0 | -0.0521 |  |  |  |  |  |  |  |  |
| 2108.6 | -0.01 | -0.0521 |  |  |  |  |  |  |  |  |
| 2099 | -0.26 | -0.2271 |  |  |  |  |  |  |  |  |
| 2111.3 | -0.08 | -0.0561 |  |  |  |  |  |  |  |  |
| 2090.7 | 0.2 | 0.1319 |  |  |  |  |  |  |  |  |
| 2099.3 | -0.15 | -0.2251 |  |  |  |  |  |  |  |  |
| 2101.9 | -0.15 | -0.1311 |  |  |  |  |  |  |  |  |
| 2096.9 | -0.12 | -0.1821 |  |  |  |  |  |  |  |  |
| 2106.1 | -0.06 | -0.0181 |  |  |  |  |  |  |  |  |
| 2103.5 | -0.14 | -0.0551 |  |  |  |  |  |  |  |  |
| 2117.3 | -0.01 | -0.0341 |  |  |  |  |  |  |  |  |
| 2132.5 | 0 | -0.0561 |  |  |  |  |  |  |  |  |
| 2128.9 | -0.01 | -0.0591 |  |  |  |  |  |  |  |  |
| 2125.8 | -0.14 | -0.2201 |  |  |  |  |  |  |  |  |
| 2130 | -0.05 | -0.0551 |  |  |  |  |  |  |  |  |
| 2141.2 | -0.01 | -0.0001 |  |  |  |  |  |  |  |  |
| 2152.8 | -0.14 | -0.0651 |  |  |  |  |  |  |  |  |
| 2159 | 0.04 | 0.0219 |  |  |  |  |  |  |  |  |
| 2156.5 | 0 | 0.0269 |  |  |  |  |  |  |  |  |
| 2156.5 | 0 | 0.0269 |  |  |  |  |  |  |  |  |
| 2152.8 | 0.03 | -0.0181 |  |  |  |  |  |  |  |  |
| 2159.1 | 0.1 | 0.0279 |  |  |  |  |  |  |  |  |
| 2155 | 0.04 | 0.0339 |  |  |  |  |  |  |  |  |
| 2149.8 | -0.13 | -0.1901 |  |  |  |  |  |  |  |  |
| 2144.7 | -0.15 | -0.0881 |  |  |  |  |  |  |  |  |
| 2154 | -0.05 | 0.0179 |  |  |  |  |  |  |  |  |

| Table S3: We studied GLONASS (L1 and L2) derived tide gauge (TG) measurements for the current study. In this table column 1 indicates time in hours from 01 October 2019 to 31 December 2019. Column 2, the GLONASS (L1) signal derived results which is subtracted from its mean. Column 3 indicates corresponding GSI-TG values, from which its mean value is subtracted. Similarly, column 4 time in hours, column 5 is GLONASS (L2) signal derived results and column 6 is GSI-TG. | | | | | | |
| --- | --- | --- | --- | --- | --- | --- |
| Time in Hours from 01 October 2019 | GLONASS-TG  (L1)  (Mean-Derived) | GSI-TG  (Derived -Mean) |  | Time in Hours from 01 October 2019 | GLONASS-TG  (L2)  (Mean-Derived) | GSI-TG  (Derived -Mean) |
| 22.8 | 0.0412 | 0.1219 |  | 20.8 | 0.0798 | 0.1539 |
| 17 | 0.3512 | 0.2679 |  | 8.9 | 0.0098 | 0.0189 |
| 17 | 0.3512 | 0.2679 |  | 8.9 | 0.0198 | 0.0189 |
| 13.8 | 0.2112 | 0.1359 |  | 13.6 | 0.0498 | 0.1279 |
| 13.8 | 0.2012 | 0.1359 |  | 11.7 | 0.0098 | 0.0189 |
| 21.8 | 0.1812 | 0.1239 |  | 11.7 | -0.0402 | 0.0189 |
| 21.8 | 0.1812 | 0.1239 |  | 13.6 | 0.1898 | 0.1149 |
| 13.6 | 0.1212 | 0.1149 |  | 13.6 | 0.1898 | 0.1149 |
| 13.6 | 0.1112 | 0.1149 |  | 19.3 | 0.1398 | 0.1859 |
| 19.3 | 0.2412 | 0.1859 |  | 19.3 | 0.1598 | 0.1859 |
| 19.3 | 0.2412 | 0.1859 |  | 30.9 | 0.0898 | 0.1519 |
| 29.9 | 0.1212 | 0.2019 |  | 30.9 | 0.0898 | 0.1519 |
| 29.7 | 0.1912 | 0.2099 |  | 46.7 | 0.1598 | 0.1459 |
| 35.5 | 0.0112 | -0.0171 |  | 46.7 | 0.0898 | 0.1459 |
| 35.5 | 0.0112 | -0.0171 |  | 47.2 | 0.2098 | 0.1559 |
| 58.3 | 0.1612 | 0.2149 |  | 47.2 | 0.2198 | 0.1559 |
| 59.5 | 0.1612 | 0.1399 |  | 43.9 | 0.2298 | 0.1959 |
| 51.7 | 0.2212 | 0.2959 |  | 43.9 | 0.1798 | 0.1959 |
| 51.7 | 0.2312 | 0.2959 |  | 60.5 | 0.1298 | 0.1539 |
| 61.5 | 0.1312 | 0.2139 |  | 48.7 | 0.1498 | 0.1799 |
| 61.5 | 0.1312 | 0.2139 |  | 48.7 | 0.1498 | 0.1799 |
| 51.2 | 0.2312 | 0.2789 |  | 61.5 | 0.2398 | 0.2139 |
| 51.2 | 0.2312 | 0.2789 |  | 83.8 | 0.2098 | 0.2649 |
| 86.9 | 0.2312 | 0.2679 |  | 83.8 | 0.2098 | 0.2649 |
| 107.7 | 0.2312 | 0.2169 |  | 107.7 | 0.1498 | 0.2169 |
| 107.7 | 0.2312 | 0.2169 |  | 107.7 | 0.1898 | 0.2169 |
| 115.9 | 0.3212 | 0.2979 |  | 142.5 | 0.1398 | 0.1169 |
| 115.9 | 0.3212 | 0.2979 |  | 142.5 | 0.1198 | 0.1169 |
| 109.6 | 0.2012 | 0.1559 |  | 128.7 | 0.1698 | 0.1789 |
| 109.6 | 0.1912 | 0.1559 |  | 128.7 | 0.1798 | 0.1789 |
| 132.3 | -0.0088 | 0.0759 |  | 123.9 | 0.1998 | 0.2239 |
| 132.3 | 0.0212 | 0.0759 |  | 123.9 | 0.1898 | 0.2239 |
| 134.6 | 0.0312 | -0.0191 |  | 152.6 | 0.0398 | 0.0499 |
| 134.6 | 0.0412 | -0.0191 |  | 152.6 | 0.0498 | 0.0499 |
| 142.5 | 0.0412 | 0.1169 |  | 150.8 | 0.1498 | 0.0709 |
| 158.4 | 0.0312 | -0.0121 |  | 146.7 | 0.0998 | 0.1049 |
| 156.2 | 0.0012 | -0.0021 |  | 146.7 | 0.0998 | 0.1049 |
| 156.2 | 0.0012 | -0.0021 |  | 188.4 | 0.1298 | 0.0509 |
| 150.6 | 0.0212 | 0.0809 |  | 172.4 | 0.1698 | 0.1169 |
| 150.6 | 0.0112 | 0.0809 |  | 172.4 | 0.1398 | 0.1169 |
| 152.6 | 0.0612 | 0.0499 |  | 172.1 | 0.1398 | 0.1379 |
| 152.6 | 0.0712 | 0.0499 |  | 190.8 | 0.0198 | 0.0709 |
| 166.8 | 0.0212 | 0.0809 |  | 190.8 | 0.0298 | 0.0709 |
| 166.8 | 0.0412 | 0.0809 |  | 173.2 | 0.0698 | 0.0979 |
| 150.8 | -0.0088 | 0.0709 |  | 173.2 | 0.0598 | 0.0979 |
| 150.8 | 0.0012 | 0.0709 |  | 181.3 | 0.1498 | 0.1119 |
| 157.3 | -0.0488 | 0.0259 |  | 181.3 | 0.1498 | 0.1119 |
| 157.3 | -0.0488 | 0.0259 |  | 204.3 | 0.0598 | 0.0959 |
| 165.3 | -0.0688 | 0.0159 |  | 204.3 | 0.0698 | 0.0959 |
| 188.2 | -0.0188 | 0.0149 |  | 200.4 | 0.0498 | 0.0899 |
| 188.2 | -0.0188 | 0.0149 |  | 200.4 | 0.0398 | 0.0899 |
| 174.3 | 0.1012 | 0.1699 |  | 205.1 | 0.0798 | 0.0359 |
| 174.3 | 0.1012 | 0.1699 |  | 205.1 | 0.0698 | 0.0359 |
| 190.8 | 0.1012 | 0.0709 |  | 222.1 | 0.0598 | 0.0389 |
| 173.3 | 0.1712 | 0.1199 |  | 232.3 | -0.0002 | -0.0291 |
| 173.3 | 0.1812 | 0.1199 |  | 226.7 | 0.0898 | 0.0519 |
| 171.5 | 0.1812 | 0.1439 |  | 226.7 | 0.0998 | 0.0519 |
| 182.9 | -0.0088 | 0.0559 |  | 252 | 0.1198 | 0.1289 |
| 182.9 | 0.0012 | 0.0529 |  | 254.3 | 0.0498 | 0.1079 |
| 173.2 | 0.0412 | 0.1009 |  | 254.3 | 0.0498 | 0.1079 |
| 173.2 | 0.0312 | 0.0979 |  | 262 | 0.0298 | 0.0469 |
| 181.3 | 0.1012 | 0.1119 |  | 262 | -0.0202 | 0.0469 |
| 181.3 | 0.1012 | 0.1119 |  | 242.7 | 0.0398 | 0.0729 |
| 205.1 | 0.1212 | 0.0619 |  | 242.7 | 0.0598 | 0.0729 |
| 205.1 | 0.1112 | 0.0619 |  | 275 | 0.0998 | 0.0509 |
| 222.3 | 0.0412 | 0.0149 |  | 272 | 0.0398 | 0.0029 |
| 222.3 | 0.0412 | 0.0149 |  | 280.2 | 0.1098 | 0.1339 |
| 230.4 | -0.0388 | 0.0269 |  | 280.2 | 0.1098 | 0.1339 |
| 238.2 | 0.0412 | -0.0061 |  | 276.9 | 0.1198 | 0.1469 |
| 238.2 | 0.0312 | -0.0061 |  | 276.9 | 0.0998 | 0.1469 |
| 223.9 | 0.1312 | 0.0519 |  | 292.4 | 0.1998 | 0.1379 |
| 238.6 | 0.0112 | 0.0259 |  | 292.4 | 0.2098 | 0.1379 |
| 238.6 | 0.0012 | 0.0259 |  | 291.8 | 0.1298 | 0.1849 |
| 237 | -0.0888 | -0.0771 |  | 304.1 | 0.0598 | 0.0319 |
| 237 | -0.0888 | -0.0771 |  | 304.1 | 0.0498 | 0.0319 |
| 227 | -0.0088 | 0.0549 |  | 301 | 0.1098 | 0.0859 |
| 234.8 | -0.1288 | -0.0831 |  | 326.1 | 0.0498 | 0.0669 |
| 252.1 | 0.0512 | 0.1229 |  | 326.1 | 0.0398 | 0.0669 |
| 243.9 | 0.0112 | 0.0769 |  | 317 | 0.0698 | -0.0041 |
| 243.9 | 0.0112 | 0.0769 |  | 330.8 | -0.0502 | -0.0041 |
| 254.3 | 0.0312 | 0.1079 |  | 330.8 | -0.0402 | -0.0041 |
| 262 | -0.0088 | 0.0469 |  | 355.4 | -0.1902 | -0.1101 |
| 262 | -0.0188 | 0.0469 |  | 342.1 | 0.0098 | -0.0251 |
| 246.7 | 0.0012 | 0.0519 |  | 342.1 | 0.0098 | -0.0251 |
| 246.7 | 0.0112 | 0.0519 |  | 346.9 | -0.0402 | -0.1071 |
| 261.3 | -0.0688 | 0.0159 |  | 354.9 | -0.0802 | -0.0921 |
| 245.3 | 0.1312 | 0.0489 |  | 354.9 | -0.0302 | -0.0921 |
| 245.3 | 0.1212 | 0.0489 |  | 338.1 | 0.1198 | 0.0679 |
| 253 | 0.1512 | 0.1279 |  | 338.1 | 0.1098 | 0.0679 |
| 253 | 0.1412 | 0.1279 |  | 363.6 | -0.1102 | -0.0601 |
| 259 | 0.0312 | 0.0079 |  | 378.2 | 0.0698 | -0.0031 |
| 259 | -0.0088 | 0.0079 |  | 405.2 | 0.0498 | 0.0009 |
| 275.8 | 0.0312 | 0.0809 |  | 397.2 | -0.1602 | -0.0791 |
| 275.8 | 0.0112 | 0.0809 |  | 403.7 | 0.0598 | 0.0129 |
| 283.7 | 0.0612 | 0.0799 |  | 403.7 | 0.0498 | 0.0129 |
| 269 | 0.1712 | 0.0909 |  | 394.6 | -0.0702 | -0.1401 |
| 270.5 | -0.0288 | 0.0309 |  | 402.2 | 0.0398 | 0.0619 |
| 270.5 | 0.0012 | 0.0309 |  | 402.2 | 0.0198 | 0.0619 |
| 274.9 | 0.1112 | 0.0699 |  | 413.8 | 0.0798 | 0.0339 |
| 276.9 | 0.0912 | 0.1469 |  | 413.8 | 0.0898 | 0.0339 |
| 276.9 | 0.0912 | 0.1469 |  | 423.8 | 0.0498 | 0.0199 |
| 302.2 | -0.0188 | 0.0269 |  | 419.7 | -0.0902 | -0.0981 |
| 302.2 | -0.0188 | 0.0269 |  | 426.8 | 0.1698 | 0.0869 |
| 304.1 | 0.0912 | 0.0319 |  | 428.6 | 0.0298 | 0.0319 |
| 292.9 | 0.1112 | 0.0669 |  | 428.6 | 0.0398 | 0.0319 |
| 292.9 | 0.1212 | 0.0669 |  | 418.4 | -0.0402 | -0.0571 |
| 323.7 | 0.0112 | -0.0071 |  | 418.4 | -0.1002 | -0.0571 |
| 323.7 | 0.0112 | -0.0071 |  | 445.7 | 0.0398 | -0.0411 |
| 317.7 | 0.0012 | -0.0291 |  | 450.7 | 0.1298 | 0.0729 |
| 317.7 | 0.0012 | -0.0291 |  | 450.7 | 0.1298 | 0.0729 |
| 326.1 | 0.0412 | 0.0669 |  | 452.7 | 0.1698 | 0.1139 |
| 326.1 | 0.0412 | 0.0669 |  | 452.7 | 0.1698 | 0.1139 |
| 334 | 0.0412 | -0.0121 |  | 463.6 | 0.0498 | 0.0979 |
| 334 | 0.0312 | -0.0121 |  | 463.6 | 0.0298 | 0.0979 |
| 342.1 | -0.0088 | -0.0251 |  | 462 | 0.1598 | 0.1439 |
| 342.1 | 0.0012 | -0.0251 |  | 462 | 0.1398 | 0.1439 |
| 358.1 | -0.1088 | -0.1161 |  | 460.7 | 0.1698 | 0.1759 |
| 358.1 | -0.1288 | -0.1161 |  | 460.7 | 0.1798 | 0.1759 |
| 356.1 | -0.0788 | -0.1191 |  | 483.9 | 0.1498 | 0.1259 |
| 356.1 | -0.0688 | -0.1191 |  | 485.6 | 0.1198 | 0.1399 |
| 342.4 | -0.0088 | -0.0461 |  | 485.6 | 0.1298 | 0.1399 |
| 342.4 | 0.0012 | -0.0461 |  | 481.5 | 0.0498 | 0.0939 |
| 348.8 | 0.0012 | -0.0411 |  | 481.5 | 0.0498 | 0.0939 |
| 348.8 | 0.0012 | -0.0411 |  | 509.1 | 0.0898 | 0.1539 |
| 338.1 | 0.0112 | 0.0679 |  | 509.1 | 0.0998 | 0.1539 |
| 338.1 | -0.0088 | 0.0679 |  | 517.5 | 0.0298 | 0.0279 |
| 363.6 | 0.0212 | -0.0601 |  | 511.6 | 0.1598 | 0.1499 |
| 403.3 | 0.0412 | 0.0269 |  | 511.6 | 0.1298 | 0.1499 |
| 404.7 | 0.0612 | 0.0259 |  | 506.8 | 0.1698 | 0.1449 |
| 404.7 | 0.0712 | 0.0259 |  | 506.8 | 0.1498 | 0.1449 |
| 386.5 | 0.0012 | 0.0369 |  | 521.9 | -0.0402 | -0.0101 |
| 404.3 | 0.0112 | -0.0191 |  | 521.9 | -0.0502 | -0.0101 |
| 413.8 | 0.0412 | 0.0339 |  | 531.5 | 0.0998 | 0.1479 |
| 413.8 | 0.0312 | 0.0339 |  | 538.5 | 0.1298 | 0.1259 |
| 421.9 | -0.0988 | -0.0381 |  | 538.5 | 0.1398 | 0.1259 |
| 429.6 | 0.0012 | 0.0059 |  | 533.8 | 0.1698 | 0.1319 |
| 429.6 | -0.0188 | 0.0059 |  | 533.8 | 0.1598 | 0.1319 |
| 423.8 | 0.0612 | 0.0269 |  | 539.5 | 0.0998 | 0.0959 |
| 430.1 | 0.0412 | 0.0299 |  | 573.7 | 0.0298 | 0.0199 |
| 412.8 | 0.1112 | 0.0709 |  | 556.1 | 0.0598 | 0.0859 |
| 412.8 | 0.1312 | 0.0709 |  | 556.1 | 0.0698 | 0.0859 |
| 428.6 | -0.0188 | 0.0319 |  | 583.3 | 0.0198 | 0.0079 |
| 428.6 | -0.0188 | 0.0319 |  | 583.3 | 0.0298 | 0.0079 |
| 443.6 | -0.0388 | 0.0009 |  | 594.2 | -0.0802 | -0.0641 |
| 443.6 | 0.0012 | 0.0009 |  | 590.1 | -0.0002 | 0.0419 |
| 445.7 | 0.0312 | -0.0411 |  | 596.2 | -0.0702 | -0.0491 |
| 445.7 | 0.0212 | -0.0411 |  | 586.1 | 0.0098 | 0.0929 |
| 467.3 | -0.0188 | -0.0211 |  | 612.8 | 0.2498 | 0.1729 |
| 467.3 | -0.0088 | -0.0211 |  | 605.2 | 0.0798 | 0.0249 |
| 469.6 | -0.0088 | -0.0771 |  | 605.2 | 0.0798 | 0.0249 |
| 463.6 | 0.0712 | 0.0979 |  | 621.1 | 0.0198 | -0.0111 |
| 473.3 | 0.0412 | 0.0219 |  | 621.1 | 0.0098 | -0.0111 |
| 473.3 | -0.0188 | 0.0219 |  | 615.2 | 0.0698 | 0.0969 |
| 458.3 | 0.0812 | 0.1429 |  | 615.2 | 0.0498 | 0.0969 |
| 458.3 | 0.0812 | 0.1389 |  | 609.6 | 0.0898 | 0.0419 |
| 485.6 | 0.0812 | 0.1399 |  | 663.1 | 0.1498 | 0.0859 |
| 485.6 | 0.0612 | 0.1399 |  | 657.8 | -0.1202 | -0.1171 |
| 493.6 | -0.0488 | -0.0671 |  | 657.8 | -0.0802 | -0.1171 |
| 493.6 | -0.0988 | -0.0671 |  | 682.6 | -0.1502 | -0.1291 |
| 489.6 | -0.0088 | 0.0499 |  | 677 | 0.1598 | 0.0989 |
| 489.6 | 0.0212 | 0.0499 |  | 677 | 0.1398 | 0.0989 |
| 483.1 | 0.0512 | 0.1109 |  | 687 | 0.0898 | 0.0999 |
| 500.5 | 0.0812 | 0.0959 |  | 687 | 0.0798 | 0.0999 |
| 500.5 | 0.0812 | 0.0959 |  | 690.3 | 0.0798 | 0.1309 |
| 484.3 | 0.1412 | 0.1179 |  | 681.7 | -0.1702 | -0.1431 |
| 484.3 | 0.1312 | 0.1179 |  | 700.6 | 0.1398 | 0.1559 |
| 517.5 | 0.0312 | 0.0279 |  | 700.6 | 0.1198 | 0.1559 |
| 517.5 | 0.0312 | 0.0279 |  | 709 | 0.0398 | -0.0201 |
| 525.4 | 0.0312 | 0.1079 |  | 709 | 0.0298 | -0.0201 |
| 516.6 | -0.0688 | 0.0129 |  | 698.3 | 0.1798 | 0.1789 |
| 516.6 | -0.0688 | 0.0129 |  | 698.3 | 0.1598 | 0.1789 |
| 541.5 | 0.0812 | 0.0179 |  | 732.9 | -0.0502 | -0.1171 |
| 541.5 | 0.0912 | 0.0179 |  | 740.8 | 0.0798 | 0.0749 |
| 549.7 | -0.0088 | 0.0739 |  | 740.8 | 0.0498 | 0.0749 |
| 549.7 | 0.0112 | 0.0739 |  | 728.9 | 0.0098 | 0.0009 |
| 539.5 | 0.0212 | 0.0959 |  | 728.9 | 0.0198 | 0.0009 |
| 539.5 | 0.0412 | 0.0959 |  | 731.7 | -0.0402 | -0.1021 |
| 555.1 | 0.0112 | 0.0929 |  | 739.3 | 0.1398 | 0.0729 |
| 557.2 | 0.1412 | 0.0919 |  | 750.9 | 0.0898 | 0.0779 |
| 557.2 | 0.1112 | 0.0919 |  | 750.9 | 0.0898 | 0.0779 |
| 573.7 | 0.1012 | 0.0199 |  | 766.7 | 0.0898 | 0.0509 |
| 556.2 | 0.1212 | 0.1199 |  | 744.8 | 0.0598 | 0.0669 |
| 556.1 | 0.0512 | 0.0859 |  | 744.8 | 0.0298 | 0.0669 |
| 556.1 | 0.0412 | 0.0859 |  | 756.8 | -0.1502 | -0.1561 |
| 564.2 | 0.1112 | 0.0689 |  | 756.8 | -0.1402 | -0.1561 |
| 564.2 | 0.1112 | 0.0689 |  | 755.5 | -0.1302 | -0.1371 |
| 579 | 0.0512 | 0.0319 |  | 755.5 | -0.1102 | -0.1371 |
| 579 | 0.0212 | 0.0319 |  | 782.8 | 0.0598 | -0.0231 |
| 583.3 | 0.0512 | 0.0079 |  | 790.6 | 0.0898 | 0.1479 |
| 583.3 | 0.0512 | 0.0079 |  | 790.6 | 0.0698 | 0.1479 |
| 578.8 | -0.0188 | 0.0369 |  | 787.8 | 0.1598 | 0.1409 |
| 588 | 0.1412 | 0.1109 |  | 787.8 | 0.1598 | 0.1409 |
| 588 | 0.1412 | 0.1109 |  | 789.8 | 0.1798 | 0.1589 |
| 605.2 | 0.0512 | 0.0249 |  | 789.8 | 0.1798 | 0.1589 |
| 605.2 | 0.0412 | 0.0249 |  | 771.2 | 0.0498 | 0.1009 |
| 621.1 | 0.0212 | -0.0111 |  | 771.2 | 0.0298 | 0.1009 |
| 621.1 | 0.0112 | -0.0111 |  | 800.7 | 0.0098 | 0.0619 |
| 621.5 | 0.0612 | 0.0239 |  | 797.6 | 0.1598 | 0.1219 |
| 621.5 | 0.0612 | 0.0239 |  | 797.8 | 0.1998 | 0.1199 |
| 612.2 | 0.1912 | 0.1439 |  | 822.6 | 0.0598 | 0.0489 |
| 612.2 | 0.2012 | 0.1439 |  | 835.1 | -0.1102 | -0.0511 |
| 619.9 | 0.0012 | -0.0251 |  | 835.1 | -0.0902 | -0.0511 |
| 619.9 | 0.0012 | -0.0251 |  | 846.3 | 0.1198 | 0.0899 |
| 642.5 | 0.0012 | 0.0029 |  | 862.5 | 0.1198 | 0.0519 |
| 642.5 | 0.0012 | 0.0029 |  | 840.6 | 0.0098 | 0.0759 |
| 645 | 0.0012 | -0.0461 |  | 840.6 | 0.0098 | 0.0759 |
| 645 | -0.0088 | -0.0461 |  | 845.5 | 0.0698 | 0.0859 |
| 629.6 | 0.0212 | 0.0409 |  | 845.5 | 0.0698 | 0.0859 |
| 629.6 | 0.0112 | 0.0409 |  | 872.5 | 0.0398 | 0.1049 |
| 644.2 | -0.0688 | -0.0561 |  | 872.5 | 0.0498 | 0.1049 |
| 644.2 | -0.0688 | -0.0561 |  | 875.5 | 0.1798 | 0.1199 |
| 635.9 | 0.1512 | 0.1019 |  | 875.5 | 0.1698 | 0.1199 |
| 635.9 | 0.1412 | 0.1019 |  | 870.9 | 0.1598 | 0.0799 |
| 641.9 | 0.0412 | 0.0039 |  | 870.9 | 0.1498 | 0.0799 |
| 641.9 | 0.0312 | 0.0039 |  | 866.7 | 0.0998 | 0.0619 |
| 658.7 | -0.0088 | -0.0761 |  | 866.7 | 0.0998 | 0.0619 |
| 658.7 | -0.0088 | -0.0761 |  | 910.8 | 0.0198 | 0.0089 |
| 666.5 | 0.0212 | -0.0071 |  | 910.8 | 0.0298 | 0.0089 |
| 653.1 | 0.0612 | -0.0011 |  | 893.2 | 0.0698 | -0.0091 |
| 653.1 | 0.0412 | -0.0011 |  | 893.2 | 0.0598 | -0.0091 |
| 661.1 | -0.0188 | 0.0589 |  | 924.3 | 0.0598 | 0.0249 |
| 653.4 | 0.0112 | -0.0181 |  | 924.3 | 0.0698 | 0.0249 |
| 653.4 | 0.0212 | -0.0181 |  | 932.3 | 0.0198 | -0.0481 |
| 652.2 | -0.0388 | 0.0429 |  | 920.4 | 0.0498 | -0.0281 |
| 652.2 | -0.0388 | 0.0429 |  | 920.4 | 0.0398 | -0.0281 |
| 651.7 | -0.0288 | 0.0369 |  | 919 | -0.0502 | -0.0241 |
| 650.5 | 0.1012 | 0.0919 |  | 919 | -0.0602 | -0.0241 |
| 650.5 | 0.1112 | 0.0919 |  | 925.2 | 0.0798 | 0.0269 |
| 677 | 0.0812 | 0.0989 |  | 925.2 | 0.0698 | 0.0269 |
| 677 | 0.0612 | 0.0989 |  | 917.2 | -0.0402 | -0.0021 |
| 692 | 0.0612 | 0.0479 |  | 954.8 | 0.0598 | -0.0161 |
| 692 | 0.0712 | 0.0479 |  | 952.3 | -0.0002 | 0.0039 |
| 679.3 | -0.0988 | -0.0531 |  | 974.1 | 0.2498 | 0.1849 |
| 679.3 | -0.0888 | -0.0531 |  | 972 | 0.1198 | 0.1189 |
| 675.8 | 0.1412 | 0.0939 |  | 979.3 | 0.1298 | 0.1739 |
| 675.8 | 0.1512 | 0.0939 |  | 979.3 | 0.1298 | 0.1739 |
| 691.8 | 0.1212 | 0.0709 |  | 981.3 | 0.1798 | 0.1939 |
| 691.8 | 0.1112 | 0.0709 |  | 981.3 | 0.1598 | 0.1939 |
| 709 | 0.0412 | -0.0201 |  | 962.7 | 0.0598 | 0.1299 |
| 709 | 0.0312 | -0.0201 |  | 992 | 0.0398 | -0.0331 |
| 716.7 | 0.1112 | 0.0489 |  | 1000.2 | 0.1098 | 0.1139 |
| 716.7 | 0.0912 | 0.0489 |  | 1000.2 | 0.1098 | 0.1139 |
| 707.1 | -0.1088 | -0.0961 |  | 994.9 | -0.0702 | -0.0031 |
| 707.1 | -0.0988 | -0.0961 |  | 994.9 | -0.0802 | -0.0031 |
| 701.3 | 0.0412 | 0.1149 |  | 996.9 | 0.1198 | 0.0999 |
| 701.3 | 0.0512 | 0.1149 |  | 996.9 | 0.0998 | 0.0999 |
| 708.3 | -0.0788 | -0.0771 |  | 1024.1 | 0.0598 | 0.0149 |
| 708.3 | -0.0788 | -0.0771 |  | 1024.1 | 0.0498 | 0.0149 |
| 742.8 | 0.0412 | 0.0769 |  | 1035.3 | 0.1798 | 0.1699 |
| 742.8 | 0.0212 | 0.0769 |  | 1050.8 | -0.0502 | -0.0121 |
| 733.6 | -0.0188 | -0.0981 |  | 1050.8 | -0.0402 | -0.0121 |
| 733.6 | -0.0188 | -0.0981 |  | 1062.1 | 0.0098 | -0.0741 |
| 741.8 | 0.1812 | 0.1029 |  | 1062.1 | 0.0098 | -0.0741 |
| 741.8 | 0.1812 | 0.1029 |  | 1066.3 | -0.2502 | -0.2601 |
| 741.4 | 0.0312 | 0.0589 |  | 1075 | -0.0802 | -0.0241 |
| 741.4 | 0.0112 | 0.0589 |  | 1075 | -0.0302 | -0.0241 |
| 750.9 | 0.0512 | 0.0779 |  | 1088.5 | -0.0502 | -0.1191 |
| 750.9 | 0.0412 | 0.0779 |  | 1084.7 | 0.0798 | 0.0159 |
| 759 | -0.0688 | -0.1161 |  | 1084.7 | 0.0898 | 0.0159 |
| 759 | -0.0688 | -0.1161 |  | 1098.2 | 0.0698 | 0.0649 |
| 766.7 | 0.0412 | 0.0509 |  | 1117.3 | -0.1902 | -0.1661 |
| 766.7 | 0.0212 | 0.0509 |  | 1117.3 | -0.1602 | -0.1661 |
| 746.4 | 0.0512 | 0.1079 |  | 1123.8 | 0.0598 | -0.0111 |
| 767.2 | 0.0412 | 0.0539 |  | 1123.8 | 0.0498 | -0.0111 |
| 767.2 | 0.0412 | 0.0539 |  | 1111.8 | 0.0098 | -0.0421 |
| 749.9 | 0.1212 | 0.1079 |  | 1111.8 | 0.0198 | -0.0421 |
| 749.9 | 0.1112 | 0.1079 |  | 1114.6 | -0.0702 | -0.1441 |
| 765.6 | -0.0188 | 0.0559 |  | 1122.2 | 0.0198 | -0.0641 |
| 765.6 | -0.0188 | 0.0559 |  | 1133.8 | 0.0798 | 0.0959 |
| 749.8 | 0.1112 | 0.1009 |  | 1133.8 | 0.0898 | 0.0959 |
| 780.5 | 0.0412 | -0.0391 |  | 1143.8 | 0.0498 | 0.0359 |
| 780.5 | 0.0312 | -0.0391 |  | 1139.7 | -0.0902 | -0.0331 |
| 782.8 | 0.0412 | -0.0231 |  | 1150.1 | 0.1598 | 0.1429 |
| 782.8 | 0.0412 | -0.0231 |  | 1150.1 | 0.2198 | 0.1429 |
| 773.8 | 0.1512 | 0.1249 |  | 1146.8 | 0.1898 | 0.1119 |
| 773.8 | 0.1612 | 0.1249 |  | 1146.8 | 0.1698 | 0.1119 |
| 787.5 | 0.0312 | 0.1099 |  | 1132.7 | 0.1998 | 0.1179 |
| 787.5 | 0.0312 | 0.1099 |  | 1132.7 | 0.1998 | 0.1179 |
| 804.4 | -0.0088 | -0.0401 |  | 1138.4 | -0.0402 | 0.0109 |
| 804.4 | -0.0088 | -0.0401 |  | 1138.2 | 0.0398 | 0.0139 |
| 798.7 | 0.0312 | 0.1149 |  | 1138.2 | 0.0698 | 0.0139 |
| 806.5 | -0.0288 | -0.0741 |  | 1172.7 | 0.1698 | 0.1079 |
| 806.5 | -0.0388 | -0.0741 |  | 1172.7 | 0.1698 | 0.1079 |
| 810.4 | 0.0012 | -0.0351 |  | 1167.8 | -0.2102 | -0.1521 |
| 797.6 | 0.0612 | 0.1219 |  | 1167.8 | -0.1902 | -0.1521 |
| 797.6 | 0.0512 | 0.1219 |  | 1183.6 | 0.0298 | -0.0391 |
| 822.6 | 0.0512 | 0.0489 |  | 1186.3 | -0.0802 | -0.1181 |
| 822.6 | 0.0312 | 0.0489 |  | 1186.3 | -0.1302 | -0.1181 |
| 830.7 | -0.0088 | -0.0851 |  | 1178.3 | -0.0602 | -0.0041 |
| 830.7 | -0.0588 | -0.0851 |  | 1178.3 | -0.0302 | -0.0041 |
| 826.6 | -0.0088 | 0.0239 |  | 1209.6 | -0.1602 | -0.0771 |
| 837.6 | 0.0712 | 0.0279 |  | 1227.1 | -0.2202 | -0.1791 |
| 837.6 | 0.0712 | 0.0279 |  | 1245.4 | -0.0202 | -0.0881 |
| 852.2 | -0.0088 | 0.0339 |  | 1226.2 | -0.1002 | -0.1811 |
| 852.2 | 0.0212 | 0.0339 |  | 1226.2 | -0.1402 | -0.1811 |
| 846.3 | 0.0112 | 0.0899 |  | 1250.5 | -0.1902 | -0.1431 |
| 854.6 | 0.0312 | -0.0271 |  | 1258.4 | -0.1302 | -0.0811 |
| 854.6 | 0.0412 | -0.0271 |  | 1258.4 | -0.1202 | -0.0811 |
| 862.5 | 0.0412 | 0.0519 |  | 1293.7 | 0.0298 | 0.0349 |
| 862.5 | 0.0212 | 0.0519 |  | 1289.7 | 0.0098 | -0.0101 |
| 858.5 | -0.0988 | -0.0421 |  | 1299 | 0.1398 | 0.1139 |
| 846.9 | 0.0412 | 0.0849 |  | 1307.3 | -0.0302 | 0.0499 |
| 846.9 | 0.0312 | 0.0849 |  | 1314.2 | -0.0802 | -0.0521 |
| 843.3 | 0.0812 | 0.0829 |  | 1316.2 | -0.0702 | -0.0891 |
| 875.5 | 0.1912 | 0.1099 |  | 1306.1 | 0.0098 | -0.0341 |
| 878.4 | 0.0312 | 0.0609 |  | 1306.1 | -0.0502 | -0.0341 |
| 870.6 | 0.0212 | 0.0559 |  | 1323.3 | -0.0802 | -0.0491 |
| 870.6 | 0.0112 | 0.0559 |  | 1365 | 0.0898 | 0.0459 |
| 872.5 | 0.0612 | 0.1049 |  | 1365 | 0.0898 | 0.0459 |
| 872.5 | 0.0712 | 0.1049 |  | 1362.2 | 0.1198 | 0.0409 |
| 886.8 | 0.0212 | 0.0709 |  | 1370.2 | 0.2098 | 0.1619 |
| 886.8 | 0.0412 | 0.0709 |  | 1370.2 | 0.1898 | 0.1619 |
| 870.9 | 0.0012 | 0.0799 |  | 1372.2 | 0.1598 | 0.0799 |
| 866.7 | 0.0012 | 0.0619 |  | 1402.6 | -0.1502 | -0.2211 |
| 866.7 | -0.0088 | 0.0619 |  | 1410.3 | 0.0798 | 0.0379 |
| 892.1 | 0.0012 | 0.0149 |  | 1401.7 | -0.1702 | -0.2191 |
| 892.1 | 0.0112 | 0.0149 |  | 1420.6 | 0.1398 | 0.0649 |
| 908.2 | -0.0188 | -0.0471 |  | 1420.6 | 0.1198 | 0.0649 |
| 908.2 | -0.0188 | -0.0471 |  | 1436.7 | -0.1102 | -0.0321 |
| 908.6 | -0.1188 | -0.0381 |  | 1433.7 | -0.0702 | -0.0131 |
| 908.6 | -0.1188 | -0.0381 |  | 1433.7 | -0.0602 | -0.0131 |
| 902.9 | -0.0088 | -0.0331 |  | 1460.8 | 0.0798 | 0.0369 |
| 902.9 | 0.0012 | -0.0311 |  | 1460.8 | 0.0498 | 0.0369 |
| 893.2 | 0.0412 | 0.0039 |  | 1443.9 | -0.0502 | 0.0139 |
| 893.2 | 0.0312 | -0.0091 |  | 1470.9 | 0.0898 | 0.1179 |
| 915 | -0.0488 | 0.0319 |  | 1470.9 | 0.0898 | 0.1179 |
| 925.1 | 0.1112 | 0.0299 |  | 1486.7 | 0.1598 | 0.1309 |
| 942.3 | 0.0412 | -0.0431 |  | 1486.7 | 0.0898 | 0.1309 |
| 942.3 | 0.0412 | -0.0431 |  | 1480.8 | 0.0598 | 0.0359 |
| 958.2 | 0.0412 | 0.0469 |  | 1480.8 | 0.0598 | 0.0359 |
| 958.2 | 0.0312 | 0.0469 |  | 1464.8 | 0.0598 | 0.0619 |
| 958.6 | 0.0112 | 0.0949 |  | 1464.8 | 0.0298 | 0.0619 |
| 957 | -0.0888 | -0.0091 |  | 1476.8 | -0.1402 | -0.0621 |
| 957 | -0.0888 | -0.0091 |  | 1483.9 | 0.1798 | 0.1439 |
| 947 | -0.0088 | -0.0091 |  | 1500.6 | -0.1702 | -0.1371 |
| 976.1 | 0.1612 | 0.2149 |  | 1488.7 | 0.1498 | 0.0779 |
| 972 | 0.0412 | 0.1189 |  | 1488.7 | 0.1498 | 0.0779 |
| 966.7 | 0.0012 | 0.0049 |  | 1499.5 | -0.1202 | -0.0781 |
| 966.7 | 0.0112 | 0.0049 |  | 1504.8 | -0.0902 | -0.1011 |
| 965.3 | 0.1212 | 0.0429 |  | 1491.2 | 0.0498 | 0.0929 |
| 973 | 0.1512 | 0.1609 |  | 1491.2 | 0.0298 | 0.0929 |
| 973 | 0.1412 | 0.1609 |  | 1520.7 | -0.0402 | -0.0701 |
| 995.8 | 0.0312 | 0.0449 |  | 1520.7 | 0.0098 | -0.0701 |
| 995.8 | 0.0112 | 0.0449 |  | 1514.2 | -0.0602 | -0.0351 |
| 1003.7 | 0.0612 | 0.0529 |  | 1523.4 | -0.1402 | -0.1401 |
| 1001.8 | 0.0112 | 0.0689 |  | 1523.4 | -0.1202 | -0.1401 |
| 989 | 0.1712 | 0.0919 |  | 1548.2 | -0.1502 | -0.1081 |
| 990.5 | -0.0288 | 0.0439 |  | 1546.7 | -0.0902 | -0.0651 |
| 990.5 | 0.0012 | 0.0439 |  | 1544.3 | -0.1102 | -0.0901 |
| 989.3 | -0.0288 | 0.0469 |  | 1555.2 | -0.1102 | -0.1031 |
| 996.9 | 0.0912 | 0.0999 |  | 1555.2 | -0.0902 | -0.1031 |
| 996.9 | 0.0912 | 0.0999 |  | 1574.2 | -0.2802 | -0.2511 |
| 1011.8 | 0.0012 | 0.0179 |  | 1560.6 | -0.1502 | -0.0891 |
| 1011.8 | -0.0088 | 0.0179 |  | 1563 | -0.1002 | -0.0451 |
| 1022.2 | -0.0188 | 0.0209 |  | 1563 | -0.0802 | -0.0451 |
| 1022.2 | -0.0188 | 0.0209 |  | 1595.5 | -0.1602 | -0.1261 |
| 1024.1 | 0.0912 | 0.0149 |  | 1595.5 | -0.1002 | -0.1261 |
| 1016.4 | -0.0788 | -0.1001 |  | 1630.8 | 0.0198 | -0.0521 |
| 1016.4 | -0.0888 | -0.1001 |  | 1630.8 | 0.0298 | -0.0521 |
| 1044.2 | -0.1288 | -0.0991 |  | 1625.2 | -0.1502 | -0.1471 |
| 1044.2 | -0.1288 | -0.0991 |  | 1625.2 | -0.1602 | -0.1471 |
| 1052.2 | -0.1188 | -0.0381 |  | 1643.1 | -0.1502 | -0.1101 |
| 1038.4 | 0.0512 | 0.0929 |  | 1672.3 | -0.0002 | -0.0161 |
| 1038.4 | 0.0512 | 0.0929 |  | 1668.2 | -0.1002 | -0.0601 |
| 1075.5 | -0.0188 | -0.0601 |  | 1694.3 | 0.0498 | 0.0679 |
| 1062.1 | -0.0088 | -0.0741 |  | 1694.3 | 0.0498 | 0.0679 |
| 1062.1 | 0.0012 | -0.0741 |  | 1702 | 0.0298 | 0.0769 |
| 1069.8 | -0.1988 | -0.1331 |  | 1682.7 | 0.0398 | 0.0079 |
| 1069.8 | -0.1788 | -0.1331 |  | 1682.7 | 0.0598 | 0.0079 |
| 1076.1 | -0.0788 | -0.0191 |  | 1720.2 | 0.1098 | 0.0509 |
| 1076.1 | -0.0688 | -0.0191 |  | 1720.2 | 0.1098 | 0.0509 |
| 1076.7 | -0.0088 | -0.0231 |  | 1714.9 | -0.0702 | -0.0811 |
| 1076.7 | 0.0112 | -0.0231 |  | 1714.9 | -0.0802 | -0.0811 |
| 1058.2 | 0.0112 | 0.0079 |  | 1739.6 | -0.2902 | -0.2291 |
| 1058.2 | -0.0088 | 0.0079 |  | 1735.7 | -0.1802 | -0.2331 |
| 1083.6 | 0.0612 | 0.0119 |  | 1738.8 | -0.3502 | -0.2851 |
| 1083.6 | 0.0212 | 0.0119 |  | 1774 | 0.1198 | 0.0509 |
| 1102.3 | 0.0912 | 0.0399 |  | 1770.8 | -0.0502 | 0.0329 |
| 1102.3 | 0.1212 | 0.0399 |  | 1770.8 | -0.0402 | 0.0329 |
| 1083.4 | 0.0512 | 0.0009 |  | 1782.1 | 0.0098 | -0.0201 |
| 1084.7 | 0.0412 | 0.0159 |  | 1782.1 | 0.0098 | -0.0201 |
| 1084.7 | 0.0412 | 0.0159 |  | 1786.3 | -0.2502 | -0.2771 |
| 1118.7 | -0.1488 | -0.1531 |  | 1795 | -0.0802 | -0.0921 |
| 1118.7 | -0.1488 | -0.1531 |  | 1795 | -0.0302 | -0.0921 |
| 1116.6 | -0.1988 | -0.1911 |  | 1778.2 | 0.1198 | 0.0819 |
| 1106.5 | 0.0012 | 0.0789 |  | 1778.2 | 0.1098 | 0.0819 |
| 1133.8 | 0.0412 | 0.0959 |  | 1803.6 | -0.1102 | -0.0341 |
| 1133.8 | 0.0312 | 0.0959 |  | 1845.2 | 0.0498 | 0.0409 |
| 1141.9 | -0.0988 | -0.0251 |  | 1837.3 | -0.1902 | -0.2031 |
| 1143.8 | 0.0612 | 0.0339 |  | 1837.3 | -0.1602 | -0.2031 |
| 1132.8 | 0.1112 | 0.1019 |  | 1843.8 | 0.0598 | 0.0349 |
| 1132.8 | 0.1312 | 0.1019 |  | 1843.8 | 0.0498 | 0.0349 |
| 1132.7 | 0.1712 | 0.1179 |  | 1831.8 | 0.0098 | -0.0711 |
| 1163.6 | -0.0388 | -0.1011 |  | 1844.3 | 0.0698 | 0.0559 |
| 1173.5 | -0.0088 | 0.0479 |  | 1842.2 | 0.0398 | -0.0151 |
| 1173.5 | -0.0188 | 0.0479 |  | 1842.2 | 0.0198 | -0.0151 |
| 1161.3 | 0.1412 | 0.0569 |  | 1853.6 | 0.1998 | 0.1639 |
| 1170.4 | -0.0388 | -0.0501 |  | 1853.6 | 0.1898 | 0.1639 |
| 1170.4 | 0.0112 | -0.0501 |  | 1859.7 | -0.0902 | -0.0871 |
| 1192.7 | -0.2288 | -0.1951 |  | 1868.6 | 0.0298 | 0.0079 |
| 1181.7 | 0.0612 | -0.0011 |  | 1868.6 | 0.0398 | 0.0079 |
| 1181.7 | 0.0212 | -0.0011 |  | 1852.7 | 0.1998 | 0.1889 |
| 1182 | -0.0088 | 0.0109 |  | 1852.7 | 0.1998 | 0.1889 |
| 1182 | -0.0088 | 0.0109 |  | 1858.4 | -0.0402 | -0.0321 |
| 1178.3 | 0.0812 | 0.0209 |  | 1858.4 | -0.1002 | -0.0321 |
| 1201.5 | -0.0388 | -0.0601 |  | 1858.2 | 0.0398 | -0.0321 |
| 1201.5 | -0.0488 | -0.0601 |  | 1891.1 | -0.1502 | -0.0701 |
| 1221.2 | -0.1388 | -0.1511 |  | 1887.8 | -0.2102 | -0.1681 |
| 1221.2 | -0.2288 | -0.1511 |  | 1887.8 | -0.1902 | -0.1681 |
| 1209.6 | -0.0088 | -0.0771 |  | 1903.6 | 0.0498 | -0.0321 |
| 1209.3 | -0.0488 | -0.0661 |  | 1903.6 | 0.0298 | -0.0321 |
| 1209.3 | -0.0488 | -0.0661 |  | 1906.3 | -0.0802 | -0.0571 |
| 1207.8 | -0.0688 | -0.0651 |  | 1906.3 | -0.1302 | -0.0571 |
| 1235.2 | -0.0388 | -0.1001 |  | 1898.3 | -0.0602 | -0.0661 |
| 1235.2 | -0.0288 | -0.1001 |  | 1898.3 | -0.0302 | -0.0661 |
| 1229.1 | -0.1188 | -0.2011 |  | 1925.6 | 0.1198 | 0.0414 |
| 1234.9 | -0.1188 | -0.0881 |  | 1921.5 | 0.0498 | 0.0169 |
| 1235.6 | -0.1088 | -0.0791 |  | 1921.5 | 0.0498 | 0.0169 |
| 1235.6 | -0.0888 | -0.0791 |  | 1941.2 | 0.0198 | -0.0191 |
| 1243.7 | -0.1188 | -0.1901 |  | 1965.4 | -0.0002 | -0.0036 |
| 1243.7 | -0.1088 | -0.1901 |  | 1965.4 | -0.0202 | -0.0036 |
| 1236.6 | -0.0688 | -0.1391 |  | 1946.2 | -0.1002 | -0.0416 |
| 1236.6 | -0.0688 | -0.1391 |  | 1961.9 | -0.0402 | -0.0921 |
| 1259.1 | -0.0288 | -0.0511 |  | 1961.9 | -0.0502 | -0.0921 |
| 1259.1 | -0.0388 | -0.0511 |  | 1973.5 | 0.0298 | -0.0301 |
| 1261.2 | -0.1088 | -0.0991 |  | 1973.5 | 0.0398 | -0.0301 |
| 1261.2 | -0.1288 | -0.0991 |  | 1979.5 | 0.0998 | 0.0334 |
| 1269.7 | -0.0088 | -0.0651 |  | 2013.7 | 0.0298 | 0.0369 |
| 1269.7 | 0.0112 | -0.0651 |  | 1996.1 | 0.0598 | -0.0136 |
| 1267.5 | -0.1188 | -0.1681 |  | 1996.1 | 0.0698 | -0.0136 |
| 1267.5 | -0.1288 | -0.1681 |  | 2009.7 | 0.0098 | -0.0641 |
| 1260.2 | -0.0788 | -0.0621 |  | 2035.2 | 0.0398 | 0.0159 |
| 1260.2 | -0.0688 | -0.0621 |  | 2035.2 | 0.0398 | 0.0159 |
| 1258.4 | -0.1588 | -0.0811 |  | 2023.3 | 0.0198 | -0.0051 |
| 1259.5 | 0.0212 | -0.0461 |  | 2023.3 | 0.0298 | -0.0051 |
| 1275.1 | -0.0288 | -0.0971 |  | 2034.3 | -0.0802 | -0.0131 |
| 1293.7 | 0.1012 | 0.0349 |  | 2030.1 | -0.0002 | 0.0654 |
| 1284.2 | 0.1112 | 0.0419 |  | 2036.2 | -0.0702 | -0.0131 |
| 1284.2 | 0.1112 | 0.0419 |  | 2026.1 | 0.0098 | 0.0434 |
| 1299 | 0.0512 | 0.1139 |  | 2033.7 | 0.0898 | 0.0059 |
| 1308 | 0.1412 | 0.0699 |  | 2055.2 | 0.0698 | 0.0759 |
| 1308 | 0.1412 | 0.0699 |  | 2055.2 | 0.0498 | 0.0759 |
| 1333.4 | -0.0988 | -0.0801 |  | 2051.1 | -0.1102 | -0.1481 |
| 1337.7 | -0.1088 | -0.1161 |  | 2051.1 | -0.0802 | -0.1481 |
| 1326.3 | -0.1488 | -0.2151 |  | 2058.3 | 0.1698 | 0.1119 |
| 1326.3 | -0.2388 | -0.2151 |  | 2084.9 | 0.0898 | 0.0469 |
| 1346.8 | -0.0188 | -0.0101 |  | 2084.9 | 0.0898 | 0.0469 |
| 1346.8 | -0.0088 | -0.0101 |  | 2082.2 | 0.1198 | 0.0829 |
| 1362.5 | 0.0012 | 0.0179 |  | 2084.2 | 0.1598 | 0.0899 |
| 1362.5 | 0.0012 | 0.0179 |  | 2084.2 | 0.1598 | 0.0899 |
| 1357.2 | 0.0312 | -0.0501 |  | 2090.2 | 0.1898 | 0.1239 |
| 1357.2 | 0.0312 | -0.0501 |  | 2092.2 | 0.1798 | 0.0959 |
| 1365 | 0.0012 | 0.0459 |  | 2092.2 | 0.1598 | 0.0959 |
| 1365 | -0.0088 | 0.0459 |  | 2132 | -0.0802 | -0.0661 |
| 1355.9 | -0.0888 | -0.1411 |  | 2140.6 | 0.1198 | 0.0439 |
| 1361.9 | 0.0412 | 0.0449 |  | 2137.7 | -0.1102 | -0.0381 |
| 1361.9 | 0.0312 | 0.0449 |  | 2153.7 | -0.0702 | -0.0101 |
| 1386.5 | 0.0212 | -0.0381 |  | 2153.7 | -0.0602 | -0.0101 |
| 1373.1 | 0.0612 | 0.0269 |  | 2153.3 | 0.0698 | -0.0051 |
| 1373.1 | 0.0412 | 0.0269 |  | 2153.3 | 0.0498 | -0.0051 |
| 1386.6 | 0.0812 | 0.0039 |  |  |  |  |
| 1373.4 | 0.0112 | 0.0319 |  |  |  |  |
| 1373.4 | 0.0212 | 0.0319 |  |  |  |  |
| 1370.2 | 0.2212 | 0.1619 |  |  |  |  |
| 1370.2 | 0.2412 | 0.1619 |  |  |  |  |
| 1370.5 | 0.1012 | 0.1269 |  |  |  |  |
| 1370.5 | 0.1112 | 0.1269 |  |  |  |  |
| 1397 | 0.0812 | 0.0049 |  |  |  |  |
| 1397 | 0.0612 | 0.0049 |  |  |  |  |
| 1392.9 | -0.0288 | 0.0169 |  |  |  |  |
| 1405.1 | -0.0788 | -0.1621 |  |  |  |  |
| 1405.1 | -0.0988 | -0.1621 |  |  |  |  |
| 1412 | 0.0612 | 0.0209 |  |  |  |  |
| 1412 | 0.0712 | 0.0209 |  |  |  |  |
| 1399.3 | -0.0988 | -0.1361 |  |  |  |  |
| 1399.3 | -0.0888 | -0.1361 |  |  |  |  |
| 1411.8 | 0.1212 | 0.0489 |  |  |  |  |
| 1411.8 | 0.1112 | 0.0489 |  |  |  |  |
| 1435.2 | -0.0888 | -0.0201 |  |  |  |  |
| 1435.2 | -0.0888 | -0.0201 |  |  |  |  |
| 1421.3 | 0.0412 | 0.0629 |  |  |  |  |
| 1421.3 | 0.0512 | 0.0629 |  |  |  |  |
| 1462.8 | 0.0412 | 0.0459 |  |  |  |  |
| 1462.8 | 0.0212 | 0.0459 |  |  |  |  |
| 1452.9 | -0.2788 | -0.1941 |  |  |  |  |
| 1445.8 | -0.0488 | 0.0189 |  |  |  |  |
| 1445.8 | -0.0388 | 0.0189 |  |  |  |  |
| 1443.6 | 0.0012 | 0.0339 |  |  |  |  |
| 1443.6 | -0.0488 | 0.0339 |  |  |  |  |
| 1461.4 | 0.0312 | 0.0569 |  |  |  |  |
| 1461.4 | 0.0112 | 0.0569 |  |  |  |  |
| 1470.9 | 0.0512 | 0.1179 |  |  |  |  |
| 1470.9 | 0.0412 | 0.1179 |  |  |  |  |
| 1479 | -0.0688 | -0.0421 |  |  |  |  |
| 1479 | -0.0688 | -0.0421 |  |  |  |  |
| 1466.4 | 0.0512 | 0.0509 |  |  |  |  |
| 1487.2 | 0.0412 | 0.0999 |  |  |  |  |
| 1487.2 | 0.0412 | 0.0999 |  |  |  |  |
| 1469.9 | 0.1212 | 0.0989 |  |  |  |  |
| 1469.9 | 0.1112 | 0.0989 |  |  |  |  |
| 1478.1 | 0.0012 | -0.0691 |  |  |  |  |
| 1469.7 | 0.1112 | 0.1169 |  |  |  |  |
| 1469.7 | 0.1912 | 0.1169 |  |  |  |  |
| 1475.5 | 0.0112 | 0.0009 |  |  |  |  |
| 1475.5 | 0.0112 | 0.0009 |  |  |  |  |
| 1510.6 | -0.0288 | -0.0441 |  |  |  |  |
| 1510.6 | -0.0188 | -0.0441 |  |  |  |  |
| 1495.2 | -0.0188 | 0.0659 |  |  |  |  |
| 1495.2 | 0.0112 | 0.0659 |  |  |  |  |
| 1509.8 | -0.0688 | -0.0161 |  |  |  |  |
| 1509.8 | -0.0788 | -0.0161 |  |  |  |  |
| 1493.8 | 0.1512 | 0.1119 |  |  |  |  |
| 1493.8 | 0.1612 | 0.1119 |  |  |  |  |
| 1507.5 | 0.0312 | -0.0361 |  |  |  |  |
| 1507.5 | 0.0312 | -0.0361 |  |  |  |  |
| 1518.7 | 0.0312 | -0.0221 |  |  |  |  |
| 1518.7 | 0.0112 | -0.0221 |  |  |  |  |
| 1517 | -0.0888 | -0.0461 |  |  |  |  |
| 1517 | -0.0888 | -0.0461 |  |  |  |  |
| 1517.8 | -0.0188 | -0.0111 |  |  |  |  |
| 1517.8 | -0.0188 | -0.0111 |  |  |  |  |
| 1538.5 | -0.0788 | -0.0841 |  |  |  |  |
| 1538.5 | -0.0388 | -0.0841 |  |  |  |  |
| 1546.7 | -0.0088 | -0.0651 |  |  |  |  |
| 1544.9 | -0.0788 | -0.1241 |  |  |  |  |
| 1582.5 | 0.0212 | -0.0521 |  |  |  |  |
| 1580.8 | -0.1188 | -0.0811 |  |  |  |  |
| 1581.5 | -0.0588 | -0.1321 |  |  |  |  |
| 1581.5 | -0.0588 | -0.1321 |  |  |  |  |
| 1596.2 | 0.0012 | -0.0811 |  |  |  |  |
| 1596.2 | 0.0012 | -0.0811 |  |  |  |  |
| 1598.3 | -0.1288 | -0.1641 |  |  |  |  |
| 1598.3 | -0.1788 | -0.1641 |  |  |  |  |
| 1606.7 | -0.1288 | -0.0801 |  |  |  |  |
| 1606.7 | -0.1388 | -0.0801 |  |  |  |  |
| 1604.6 | -0.1088 | -0.1201 |  |  |  |  |
| 1604.6 | -0.0988 | -0.1201 |  |  |  |  |
| 1584.8 | -0.0888 | -0.1721 |  |  |  |  |
| 1605.3 | -0.0688 | -0.0721 |  |  |  |  |
| 1605.3 | -0.0988 | -0.0721 |  |  |  |  |
| 1620.2 | -0.1288 | -0.0981 |  |  |  |  |
| 1620.2 | -0.1188 | -0.0981 |  |  |  |  |
| 1610 | -0.0488 | -0.0721 |  |  |  |  |
| 1620.6 | -0.1488 | -0.1001 |  |  |  |  |
| 1620.6 | -0.1288 | -0.1001 |  |  |  |  |
| 1628.6 | -0.1188 | -0.0941 |  |  |  |  |
| 1628.6 | -0.1188 | -0.0941 |  |  |  |  |
| 1670.4 | -0.0388 | -0.0171 |  |  |  |  |
| 1670.4 | -0.0688 | -0.0171 |  |  |  |  |
| 1678.2 | 0.0412 | 0.0069 |  |  |  |  |
| 1678.2 | 0.0312 | 0.0069 |  |  |  |  |
| 1678.6 | 0.0112 | -0.0111 |  |  |  |  |
| 1678.6 | 0.0012 | -0.0111 |  |  |  |  |
| 1677 | -0.0888 | -0.0101 |  |  |  |  |
| 1677 | -0.0888 | -0.0101 |  |  |  |  |
| 1674.8 | -0.1288 | -0.0531 |  |  |  |  |
| 1692.1 | 0.0512 | -0.0281 |  |  |  |  |
| 1692.1 | 0.0312 | -0.0281 |  |  |  |  |
| 1683.9 | 0.0112 | -0.0281 |  |  |  |  |
| 1683.9 | 0.0112 | -0.0281 |  |  |  |  |
| 1692 | 0.0412 | -0.0431 |  |  |  |  |
| 1692 | 0.0212 | -0.0431 |  |  |  |  |
| 1694.3 | 0.0312 | 0.0679 |  |  |  |  |
| 1694.3 | 0.0212 | 0.0679 |  |  |  |  |
| 1684.4 | -0.1288 | -0.0511 |  |  |  |  |
| 1693.1 | 0.0312 | -0.0221 |  |  |  |  |
| 1693.1 | 0.0412 | -0.0221 |  |  |  |  |
| 1701.3 | -0.0788 | -0.0031 |  |  |  |  |
| 1701.3 | -0.0688 | -0.0031 |  |  |  |  |
| 1699 | 0.0312 | 0.0239 |  |  |  |  |
| 1699 | -0.0088 | 0.0239 |  |  |  |  |
| 1715.8 | 0.0312 | -0.0371 |  |  |  |  |
| 1715.8 | 0.0112 | -0.0371 |  |  |  |  |
| 1718.2 | -0.0088 | 0.0419 |  |  |  |  |
| 1718.2 | -0.0288 | 0.0419 |  |  |  |  |
| 1721.8 | 0.0112 | 0.0119 |  |  |  |  |
| 1721.8 | -0.0188 | 0.0119 |  |  |  |  |
| 1710.5 | -0.0288 | 0.0239 |  |  |  |  |
| 1710.5 | 0.0012 | 0.0239 |  |  |  |  |
| 1707.2 | 0.2312 | 0.1539 |  |  |  |  |
| 1707.2 | 0.2312 | 0.1539 |  |  |  |  |
| 1709.3 | -0.0288 | 0.0499 |  |  |  |  |
| 1731.8 | 0.0012 | 0.0099 |  |  |  |  |
| 1731.8 | -0.0088 | 0.0099 |  |  |  |  |
| 1757.7 | 0.0012 | -0.0661 |  |  |  |  |
| 1757.7 | 0.0012 | -0.0661 |  |  |  |  |
| 1774 | 0.0412 | 0.0509 |  |  |  |  |
| 1774 | 0.0312 | 0.0509 |  |  |  |  |
| 1764.2 | -0.1288 | -0.1481 |  |  |  |  |
| 1764.2 | -0.1288 | -0.1481 |  |  |  |  |
| 1795.4 | -0.0188 | -0.0701 |  |  |  |  |
| 1782.1 | -0.0088 | -0.0201 |  |  |  |  |
| 1782.1 | 0.0012 | -0.0201 |  |  |  |  |
| 1789.8 | -0.1988 | -0.2361 |  |  |  |  |
| 1789.8 | -0.1788 | -0.2361 |  |  |  |  |
| 1798.1 | -0.1088 | -0.1271 |  |  |  |  |
| 1798.1 | -0.1288 | -0.1271 |  |  |  |  |
| 1796.1 | -0.0788 | -0.1101 |  |  |  |  |
| 1796.1 | -0.0688 | -0.1101 |  |  |  |  |
| 1782.4 | -0.0088 | -0.0371 |  |  |  |  |
| 1782.4 | 0.0012 | -0.0371 |  |  |  |  |
| 1778.2 | 0.0112 | 0.0819 |  |  |  |  |
| 1803.6 | 0.0212 | -0.0341 |  |  |  |  |
| 1804.7 | 0.0412 | -0.0391 |  |  |  |  |
| 1804.7 | 0.0412 | -0.0391 |  |  |  |  |
| 1843.3 | 0.0412 | 0.0259 |  |  |  |  |
| 1838.7 | -0.1488 | -0.1921 |  |  |  |  |
| 1838.7 | -0.1488 | -0.1921 |  |  |  |  |
| 1836.6 | -0.1988 | -0.1961 |  |  |  |  |
| 1844.7 | 0.0612 | 0.0429 |  |  |  |  |
| 1844.7 | 0.0712 | 0.0429 |  |  |  |  |
| 1826.5 | 0.0012 | -0.0241 |  |  |  |  |
| 1844.3 | 0.0112 | 0.0559 |  |  |  |  |
| 1861.9 | -0.0988 | -0.1321 |  |  |  |  |
| 1869.6 | 0.0012 | -0.0051 |  |  |  |  |
| 1869.6 | -0.0188 | -0.0051 |  |  |  |  |
| 1870.1 | 0.0412 | 0.0139 |  |  |  |  |
| 1852.8 | 0.1112 | 0.1719 |  |  |  |  |
| 1852.8 | 0.1312 | 0.1719 |  |  |  |  |
| 1868.6 | -0.0188 | 0.0079 |  |  |  |  |
| 1868.6 | -0.0188 | 0.0079 |  |  |  |  |
| 1852.7 | 0.2412 | 0.1889 |  |  |  |  |
| 1852.7 | 0.1712 | 0.1889 |  |  |  |  |
| 1883.6 | -0.0388 | -0.1211 |  |  |  |  |
| 1875.3 | -0.0288 | -0.0081 |  |  |  |  |
| 1875.3 | 0.0012 | -0.0081 |  |  |  |  |
| 1893.5 | -0.0088 | -0.0001 |  |  |  |  |
| 1893.5 | -0.0188 | -0.0001 |  |  |  |  |
| 1878.1 | 0.0112 | 0.0019 |  |  |  |  |
| 1878.1 | 0.0112 | 0.0019 |  |  |  |  |
| 1890.4 | -0.0388 | -0.0871 |  |  |  |  |
| 1907.3 | -0.0188 | -0.0951 |  |  |  |  |
| 1913.3 | -0.0188 | -0.0926 |  |  |  |  |
| 1925.6 | 0.0812 | 0.0414 |  |  |  |  |
| 1925.6 | 0.0612 | 0.0414 |  |  |  |  |
| 1921.5 | -0.0388 | 0.0169 |  |  |  |  |
| 1921.5 | -0.0488 | 0.0169 |  |  |  |  |
| 1933.6 | -0.0988 | -0.1551 |  |  |  |  |
| 1929.6 | -0.0088 | -0.0136 |  |  |  |  |
| 1929.6 | 0.0212 | -0.0136 |  |  |  |  |
| 1929.3 | -0.0488 | -0.0136 |  |  |  |  |
| 1929.3 | -0.0488 | -0.0136 |  |  |  |  |
| 1923.1 | 0.0512 | 0.0214 |  |  |  |  |
| 1927.8 | -0.0688 | 0.0074 |  |  |  |  |
| 1947.1 | -0.0688 | -0.0236 |  |  |  |  |
| 1955.2 | -0.0388 | -0.0371 |  |  |  |  |
| 1955.2 | -0.0288 | -0.0371 |  |  |  |  |
| 1949.1 | -0.0888 | -0.0236 |  |  |  |  |
| 1965.4 | 0.0312 | -0.0036 |  |  |  |  |
| 1965.4 | 0.0212 | -0.0036 |  |  |  |  |
| 1955.6 | -0.1088 | -0.0391 |  |  |  |  |
| 1955.6 | -0.0888 | -0.0391 |  |  |  |  |
| 1963.7 | -0.1188 | -0.0786 |  |  |  |  |
| 1963.7 | -0.1088 | -0.0786 |  |  |  |  |
| 1949.8 | 0.0212 | -0.0141 |  |  |  |  |
| 1949.8 | 0.0312 | -0.0141 |  |  |  |  |
| 1956.6 | -0.0688 | -0.0626 |  |  |  |  |
| 1956.6 | -0.0688 | -0.0626 |  |  |  |  |
| 1979.1 | -0.0288 | 0.0429 |  |  |  |  |
| 1979.1 | -0.0388 | 0.0429 |  |  |  |  |
| 1973.5 | -0.0288 | -0.0301 |  |  |  |  |
| 1973.5 | -0.0088 | -0.0301 |  |  |  |  |
| 1989.7 | -0.0088 | 0.0154 |  |  |  |  |
| 1989.7 | 0.0112 | 0.0154 |  |  |  |  |
| 1987.5 | -0.1188 | -0.0736 |  |  |  |  |
| 1987.5 | -0.1288 | -0.0736 |  |  |  |  |
| 1973.8 | 0.0212 | -0.0236 |  |  |  |  |
| 1973.8 | 0.0012 | -0.0236 |  |  |  |  |
| 1980.2 | -0.0788 | 0.0039 |  |  |  |  |
| 1980.2 | -0.0688 | 0.0039 |  |  |  |  |
| 1979.5 | 0.0212 | 0.0334 |  |  |  |  |
| 1979.5 | 0.0412 | 0.0334 |  |  |  |  |
| 1995.1 | 0.0112 | -0.0071 |  |  |  |  |
| 1995.1 | -0.0288 | -0.0071 |  |  |  |  |
| 2013.7 | 0.1012 | 0.0369 |  |  |  |  |
| 1996.1 | 0.0512 | -0.0136 |  |  |  |  |
| 1996.1 | 0.0412 | -0.0136 |  |  |  |  |
| 2019 | 0.0512 | 0.0534 |  |  |  |  |
| 2019 | 0.0212 | 0.0534 |  |  |  |  |
| 2034.7 | 0.0412 | -0.0101 |  |  |  |  |
| 2023.3 | 0.0512 | -0.0051 |  |  |  |  |
| 2023.3 | 0.0512 | -0.0051 |  |  |  |  |
| 2028 | 0.1412 | 0.0984 |  |  |  |  |
| 2028 | 0.1412 | 0.0984 |  |  |  |  |
| 2045.2 | 0.0512 | -0.0311 |  |  |  |  |
| 2045.2 | 0.0412 | -0.0311 |  |  |  |  |
| 2061.5 | 0.0612 | 0.0879 |  |  |  |  |
| 2061.5 | 0.0612 | 0.0879 |  |  |  |  |
| 2046.3 | -0.1488 | -0.0771 |  |  |  |  |
| 2082.6 | 0.0012 | 0.0569 |  |  |  |  |
| 2082.6 | 0.0012 | 0.0569 |  |  |  |  |
| 2077.2 | 0.0312 | 0.0079 |  |  |  |  |
| 2077.2 | 0.0312 | 0.0079 |  |  |  |  |
| 2084.9 | 0.0012 | 0.0469 |  |  |  |  |
| 2084.9 | -0.0088 | 0.0469 |  |  |  |  |
| 2075.9 | -0.0888 | -0.0571 |  |  |  |  |
| 2075.9 | -0.0488 | -0.0571 |  |  |  |  |
| 2081.9 | 0.0412 | 0.0759 |  |  |  |  |
| 2081.9 | 0.0312 | 0.0759 |  |  |  |  |
| 2106.5 | 0.0212 | -0.0111 |  |  |  |  |
| 2093.1 | 0.0612 | 0.0659 |  |  |  |  |
| 2093.1 | 0.0412 | 0.0659 |  |  |  |  |
| 2093.4 | 0.0112 | 0.0309 |  |  |  |  |
| 2093.4 | 0.0212 | 0.0309 |  |  |  |  |
| 2090.5 | 0.1012 | 0.1219 |  |  |  |  |
| 2090.5 | 0.1112 | 0.1219 |  |  |  |  |
| 2112.9 | -0.0288 | -0.0101 |  |  |  |  |
| 2132.7 | -0.1488 | -0.0821 |  |  |  |  |
| 2119.3 | -0.0988 | -0.1491 |  |  |  |  |
| 2119.3 | -0.0888 | -0.1491 |  |  |  |  |
| 2156.7 | 0.0912 | 0.0119 |  |  |  |  |
| 2141.3 | 0.0412 | -0.0061 |  |  |  |  |
| 2141.3 | 0.0512 | -0.0061 |  |  |  |  |
